# *Carissa spinarum* L. aqueous root extracts ameliorate oxidative stress-induced infertility in adult male Wistar rats

**DOI:** 10.1371/journal.pone.0355380

**Published:** 2026-08-11

**Authors:** Martin Ouma, Albert W. Nyongesa, Gladys B. Bichang’a, Edward K. Muge, Evans N. Nyaboga

**Affiliations:** 1 Department of Biochemistry, University of Nairobi, Nairobi, Kenya; 2 Department of Veterinary Anatomy and Physiology, University of Nairobi, Nairobi, Kenya; University of Toledo College of Medicine: The University of Toledo College of Medicine and Life Sciences, UNITED STATES OF AMERICA

## Abstract

The potential of *Carissa spinarum* in treatment of male infertility arising from oxidative stress remains obscure, despite its traditional use in the management of male infertility. This study evaluated the phytochemical profile and *in vivo* protective activities of *C. spinarum* extracts against oxidative stress-induced infertility in adult male Wistar rats. Root and leaf samples were extracted using solvents with different polarities, and the phytochemicals and antioxidant activities were determined. For *in vivo* studies, male Wistar rats were divided into six groups (n = 5). Group 1 (control), group 2 (metronidazole), group 3 (metronidazole and 250 mg/kg aqueous root extract, group 4 (metronidazole and 500 mg/kg aqueous root extract), group 5 (metronidazole and 1000 mg/kg aqueous root extract) and group 6 (metronidazole + vitamin E + Sustanon). The epididymis was harvested for sperm characteristics, and the testes for histology and biochemical assays. There were significantly higher (p < 0.05) phytochemicals and antioxidant activities in the leaves than the roots for all the solvents tested, except hexane. For *in vivo* studies, rats fed on aqueous root extracts at 500 mg/kg body weight (BW) showed a significant (p < 0.05) increase in testicular weight index (0.98%), sperm count (37.28 × 10^6/mL), normal sperm morphology (60.50%), catalase (14.56 × 10^−2^ nmol g^-1^ testis tissue) and reduced lipid peroxidation (1.38 ± 0.14 nmoles/g tissue), malondialdehyde content. Extracts at low and high doses showed a significant increase in viable sperm cells (88.50%) and peroxidase activity, respectively. The extracts at 250 and 500 mg/kg BW ameliorated the effects of metronidazole on testicular histology. The study affirms the potential of *C. spinarum* aqueous root extracts in the management of male infertility caused by oxidative stress-related factors.

## Introduction

According to the World Health Organization (WHO), 48 million couples and 186 million persons globally are infertile. Infertility may arise as a result of male and female factors as well as environmental and genetic factors [1]. Male factors contribute to approximately 50% of all infertility cases, most of them being associated with abnormal sperm production and other reproductive disorders [2]. Exposure to toxic environmental agents such as industrial chemicals, agrochemicals, and drugs is a leading contributor to the impairment of male reproductive physiological processes, including steroidogenesis and spermatogenesis [1]. Exposure to these agents has been reported to impair male fertility by triggering pathways leading to the production of excess reactive oxygen species (ROS) and reactive nitrogen species (RNS). The generated free radicals react with various biomolecules, including proteins, lipid membranes and DNA, resulting in protein oxidation, lipid peroxidation and DNA fragmentation that ultimately impairs spermatogenesis and related processes in the male gonads [2,3].

The use of medicinal plants in the treatment of various diseases and conditions, including infertility, has tremendously increased globally [4]. This is due to their natural abundance and minimal side effects on the body’s functional systems compared to some chemically synthesized drugs [4]. Various medicinal plants are known for their rich phytochemicals, which can prevent oxidative stress-induced ailments, supporting their use by traditional herbalists for healing and as sources of active compounds for drug design and development [5]. *Carissa spinarum* L. is one of the medicinal plants that grows in several regions of Kenya and has been reported to have male fertility-promoting properties in traditional medicine [6–8]. Phytochemical evaluation of *C. spinarum* revealed the presence of different classes of therapeutic secondary metabolites including phenols, flavonoids, tannins, alkaloids, steroids, and fatty acid esters [9] as well as characterized compounds such as (+)-isolariciresinol 3a-*O*-*β*-D-glucopyranoside, erythro-1-(3-methoxy-4-hydroxy-phenyl)-propan-1,2-diol, threo-1-(3-methoxy-4-hydroxy-phenyl)-propan-1,2-diol and (6*R*,7*S*,8*S*)-7a-[(*β*-D-glucopyranosyl)-oxy]-1-methoxyisolariciresinol from the root back of *C. spinarum* [8,10].

Despite *C. spinarum* having potential traditional medicinal applications in the treatment of oxidative stress-related conditions, including infertility, the scientific support in revealing the mechanism of the action of its aqueous root extracts on male infertility management cases, as claimed by herbalists and ethnopharmacological surveys, remains obscure and has not been explored. Its potential protective and curative effects towards infertility arising from testicular oxidative damage are equally unclear. This study aimed to evaluate the phytochemical profiles of *C. spinarum* and its testicular protective potential of aqueous root extracts of *C. spinarum* against oxidative stress-induced infertility in adult male Wistar rats, with induction being achieved by oral administration of 400 mg/kg body weight of metronidazole as reported by [11,12]. We hypothesized that the aqueous extracts of *C. spinarum* roots possess phytochemical compounds with antioxidant potential that suppress oxidative damage on testicular tissue cells that arise from oxidative stress and which can impair spermatogenesis and steroidogenesis. The study used male Wistar rats since rats have been widely used as models for fertility studies [13].

## Materials and methods

### Plant material collection, molecular identification, and sample extraction process

*Carissa spinarum* L. leaf and root samples were collected from Eor-Ewuaso village, Narok County, Kenya (coordinates −0.9690953596611817, 35.6458956346891). The samples were packed in airtight containers and transported to the University of Nairobi within 12 h of collection. Identification was made by a plant taxonomist, and samples were deposited in the herbarium at the Department of Biology, University of Nairobi (code of voucher specimen MOUNBI 2023/001). The leaf samples were stored at −80 °C for molecular authentication of the plant’s identity. The leaves and root bark of (woody roots) were peeled off and dried for two weeks, then ground into fine powder and stored at 4 °C.

Molecular identification of the plant sample was performed using DNA barcoding, as described by Pere *et al*. [14]. Genomic DNA extraction was performed using the Qiagen DNeasy® Plant Maxi Kit (Catalogue No. 68163, Qiagen, Germany), which was used to extract genomic DNA from *C. spinarum L.* leaves according to the manufacturer’s instructions. This was followed by PCR amplification targeting *trnH-PsbA* and *rbcl* regions, with the resulting sequences subjected to sequencing using Sanger sequencing chemistry (Macrogen, Netherlands)

The resulting forward and reverse sequences were used to construct contigs in BioEdit software version 5.0.9 [15], followed by manual trimming of low-quality end sequences and correction of miscalled bases based on chromatogram peaks. The cleaned sequences were BLASTed on GenBank database (https://blast.ncbi.nlm.nih.gov/Blast.cgi (accessed on 4^th^ May 2024), suitable sequences were selected based on the following features: at least 80% identity, ≥ 95% query coverage, and zero expected (E) value. The sequences were then used for phylogenetic analysis using the Bayesian phylogenetic approach of MrBayes, version 3.1.2 [16]. A total of 12,000,000 phylogenetic trees were created, and from every 1000 generations, a tree was sampled. The ‘sum p’ and ‘sum t’ in MrBayer were used in constructing the consensus tree, with the branching confidence being calculated as the posterior probabilities and later visualized in Figtree, version 1.4.3 (http://tree.bio.ed.ac.uk/).

### Chemicals

Solvents used in the experiment, including methanol, ethanol, acetone, and hexane, were HPLC grade and purchased from Sigma-Aldrich, St. Louis, MO, USA. Gallic acid, ascorbic acid, tannic acid, rutin, atropine, thiobarbituric acid (TBA), Folin-Ciocalteau reagent (Folin C), trichloroacetic acid (TCA), and poly(vinylpolypyrrolidone) (PVPP) were analytical grade and purchased from Merck Life Science UK Limited, Gillingham, United Kingdom. Distilled water was prepared in the lab and used throughout the study.

### Drugs

Drugs used in this study, including vitamin E (EvitTM400), Sustanon 250 mg, and Metronidazole (Megyl-400®), were purchased from Almond Pharmacy in Nairobi, Kenya.

### Preparation of crude extracts

To assess solvent efficacy, extraction for phytochemical analysis was performed using varying polarity solvent systems, including ethanol, methanol, hydromethanol (70%), hydroethanol (70%), n-hexane, acetone, and cold and hot distilled water. Extraction was done using the cold maceration method as described by Altemimi *et al* [17]. Additionally, hot maceration with distilled water (for *in vivo* studies) was incorporated into the extraction, as reported by Njau et al. [18]. Spectrophotometric assays were done using a UV-Vis spectrophotometer (UVmini-1240, Shimadzu, Duisburg, Germany).

### Quantitative phytochemical analysis

Phenolic content was determined in all extracts following the Folin-Ciocalteu (FC) method described by Siddiqui *et al*. [19], Briefly, 200 µL of crude extracts were transferred into clean test tubes and 1.8 mL of distilled water added. Thereafter, 200 µL of 10% Folin–Ciocalteu reagent was added to each tube, which was shaken before the addition of 7.5% sodium carbonate. The mixture was incubated in the dark at room temperature for 60 min. The absorbance was then read at 725 nm against the blank (containing all reagents except the extract). The Gallic acid standard was prepared from a 1 mg/mL stock with concentrations of 0, 20, 40, 60, 80, 100, 120, 140, 160, 180, and 200 µg/mL, respectively. The concentration of phenols was expressed as µg Gallic acid equivalent/gram of dry plant material (GAE/g).

Total flavonoid content was determined based on aluminium chloride reaction as described by Aryal *et al* [20]. Briefly, 0.25 mL of crude extracts was transferred into clean test tubes, followed by the addition of 1.5 mL of absolute Methanol. A volume of 0.1 mL of 10% Aluminium chloride solution was added to the mixture and shaken, followed by the addition of 0.1 mL of 1M sodium Acetate and 2.5 mL of distilled water, which were also shaken, and then incubated at room temperature for 30 min. The absorbance was measured at 415 nm against a blank (all other reagents except the extract). The standard rutin was prepared from a 10 mg/mL stock at concentrations of 0.0, 0.5, 1.0, 1.5, 2.0, 2.5, 3, 3.5, and 4.0 mg/mL. Concentrations of flavonoids were expressed in mg Rutin equivalent/gram of dry plant material (RE/g).

The total tannin content of each extract was measured using the Folin-Ciocalteu method as described by Makkar [21]. The concentrations of tannins were determined by first measuring the Total phenolic content using the Folin-Ciocalteu method [19], and expressed as Tannic Acid equivalents. Tannins were then removed from the tannin-containing extracts by mixing 1 mL of it with 1 mL of 1% (w/v) insoluble polyvinyl polypyrrolidone. The mixture was then incubated t at 4°C for 15 min, vortexed, and centrifuged at 3000 × g for 10 min. The supernatant containing simple phenolics was transferred into a clean Eppendorf tube and used to assay TPC, expressed as Tannic Acid equivalents. The pellets containing PVPP-bound tannins were discarded and the difference in concentration due to reduction in absorbance at 725 nm for the two assays was considered as TTC. The tannic acid standard was prepared from a 1 mg/mL stock and used to determine concentrations.

Alkaloid concentration was determined as described by Ajanal *et al*. [22]. Briefly, previously extracted samples were obtained, and 1 mL of each extract was mixed with 1 mL of 2 N HCl to dissolve the alkaloids. The mixture was shaken vigorously and centrifuged at 1000 xg for 5 *min* to sediment the unwanted debris. The supernatant (1 mL) from each was transferred to the separating funnel and washed 3 times with 10 mL of chloroform before adding 0.2 N sodium hydroxide dropwise until a neutral pH was attained. Volumes of 5 mL of each 0.1 M phosphate buffer (pH 4.7) and BCG were then added to the mixture, which was shaken to allow the formation of the yellow complex. The mixture was then extracted with 1, 2, 3, and 4 mL of chloroform by vigorous shaking, and the extracts were collected in a 10 mL volumetric flask. The standard was prepared in the same manner, except that the extract was replaced with different concentrations of Atropine from a 1 mg/mL stock.

### Evaluation of *in vitro* antioxidant activity

Ferric Reducing Antioxidant Power (FRAP) was assessed as described by Wang *et al*. [23], with ascorbic acid (200 mg) used as the standard, where 1mL of each extract was added to clean test tubes, mixed with 2 mL of 0.1M Phosphate Buffered Saline (PBS) (pH 6.6) and 2 mL of potassium ferricyanide (K3[Fe (CN)6]) (1% w/v). The mixture was placed in a water bath maintained at 50 °C for 30 min. The samples were removed and allowed to cool, then 2 mL of 10% Tetrachloroacetic acid (TCA) was added, and the mixture was centrifuged at 1000 xg at room temperature for 10 min. The supernatant (2 mL) was transferred and mixed with equal volumes of ultrapure water and 0.5 mL of 0.1% (w/v) Ferric chloride solution (FeCl3). The mixture was allowed to stand for 10 min, after which the absorbance was read at 700 nm.

Hydrogen peroxide radical scavenging activity was performed according to the method described by Ruch *et al*. [24], with ascorbic acid (200 mg) used as the standard. Briefly, 0.1 mL of crude extracts (leaves and roots) was separately added into clean test-tubes. This was followed by the addition of 0.3 mL of 1X PBS, pH 7.4, and shaking to mix. A volume of 0.6 mL of 40 mM H2O2 was added to the mixture, and the mixture was shaken to mix. After 10 min of reaction time, the absorbance of the samples, control, and Standard (Vitamin C) was read at 230 nm.

Hydrogen peroxide radical scavenging was calculated according to equation (1):


$$\begin{array}{c} {H_{\text{2}}O}_{\text{2}}\;Scavenging\;activity\;(\%)=\frac{Ao-A\text{1}}{Ao}\times \text{1}\text{0}\text{0} \end{array}$$ (1)

Where: Ao is the absorbance of control (containing Buffer, water, and Hydrogen peroxide), A1 is the absorbance of samples/standard antioxidant (Vitamin C).

### Compound identification by GC-MS analysis

Root and leaf powdered samples, macerated with ethanol and acetone (the most effective solvents of extraction according to principal component analysis), were analyzed for volatile organic compounds using a GC-MS system (GC-MS-QP2010 SE, Shimadzu, Kyoto, Japan). The program parameters were set as follows before running: the column oven temperature was set to 70°C, the injection temperature to 250°C, the injection mode to split, and the sampling time to 1 min. The carrier gas was helium (99.999%) at 8.0 psi, with a total flow rate of 13.2 mL/min, a column flow rate of 0.92 mL/min, and a split ratio of 10.1. The extracts were separated at programmed GC oven temperatures as follows: 70°C for 5 sec, 10°C/min up to 250°C for 8 min, and 5°C/min up to 270°C for 5 min. The MS adjustments included an ion source temperature of 200°C, an interface temperature of 250°C, and a solvent cut time of 3 min. The total program time dedicated to the separation was 40 min. The separated components were translated into mass spectral peaks and identified by comparison with the built-in National Institute of Standards and Technology (NIST) GC-MS mass spectral library.

### Mineral content analysis

Root and leaf samples were first prepared following the standard operating procedure at the Kenya Bureau of Standards (KEBS, TES/ING/TM/05). Briefly, the preparation was initiated by ashing 1 g of each overnight in a muffle furnace. The products were separately mixed with 10 mL of concentrated nitric acid and heated on a hot plate until a clear solution was obtained. The solution was diluted to a total volume of 50 mL using deionized water; this was performed in triplicate. Analysis of each element was performed based on its characteristic maximum absorption wavelength for macro-minerals (Ca, Na, K, Mg, P, S), trace elements (Se, Fe, Zn, Mn, Mo, Cu, Cr, Si), and non-essential elements (Ba and B) were performed using the ICP-OES instrument (5100S, Agilent) following the protocol described by Ngigi and Muraguri [25], and the concentration recorded in parts per million (ppm).

### *In vivo* animal model studies

#### Dose limit test.

To assess the safety of the extract doses for use in animal models, a dose-limiting test of C. spinarum aqueous root extract was conducted, since the root extracts of *C. spinarum* have been used as herbal remedies for other diseases without causing toxicity in humans. This test was performed according to OECD Test Guidelines 423 (limit test) [26], to determine the safety of the experimental doses. Six non-pregnant, nulliparous female Wister rats weighing 158.67 ± 1.49 g, aged 63–70 days, were randomly selected and marked for the study. The animals were fed standard rat pellets and kept at room temperature (22 ± 1.3 °C) with a 12-hour light/dark cycle. Before administering the doses, the animals fasted for 5 hours but had free access to water. A single dose of 2000 mg/kg body weight (bw) of *C. spinarum* aqueous root extract was given to the first animal. After its survival, two additional animals received the same dose and were monitored for 30 *min*, 1 hour, and 4 hours. During this period, the animals were not fed for 3 hours. Once no animal was confirmed dead, three more animals were given 1 mL of distilled water as the control group. The animals in both treatment and control groups were then observed for signs of toxicity over the next 6 hours and daily for 14 days, with body weights recorded regularly. At the end of the study (day 15), the animals were sacrificed by cervical dislocation.

### Animal handling and experimental design

All animals used in this study were handled in accordance with ethical regulations for the use of laboratory animals. Adult male Wistar rats (aged 70–90 days, weighing 130–180 g) were obtained from the Department of Biology, University of Nairobi. They were housed in a clean environment with a 12-hour light/dark cycle, relative humidity of 56–65%, and at room temperature (23 ± 1°C). The animals had free access to water and were fed standard rat pellets. They were acclimatized to the animal house for one week before the start of the study, during which they underwent 5 *min* of daily handling to familiarize them with human contact. A qualified veterinarian regularly monitored their health, and only healthy animals were used for the research. The study followed the Animal Research: Reporting of in vivo Experiments (ARRIVE) along with European Community guidelines (EEC Directive of 1986; 86/609/EEC). Ethical approval was obtained from the Biosafety, Animal Use, and Ethics Committee of the Faculty of Veterinary Medicine (Reference number FVM BAUEC/2023/528).

Thirty (30) rats were randomly assigned to six experimental groups, with 5 animals per group [27]. They received treatments with different aqueous extract doses, confirmed to be safe based on the dose limit test, conducted following OECD 423 guidelines, with no animals dying or showing signs of toxicity after a single 2000 mg/kg body weight dose, as previously reported by Dossou-Yovo et al. [28] Group 1 (Normal control) received 1 mL/200 g body weight of distilled water, Group 2 (Negative control) received 400 mg/kg metronidazole alone, Group 3 (Low extract dose group) received co-administration of 400 mg/kg body weight of metronidazole and 250 mg/kg body weight of *C. spinarum* aqueous root extract, Group 4 (Medium extract dose group) received co-administration of 400 mg/kg body weight of metronidazole and 500 mg/kg body weight of *C. spinarum* aqueous root extract, Group 5 (High extract dose group) received co-administration of 400 mg/kg body weight of metronidazole and 1000 mg/kg body weight of *C. spinarum* aqueous root extract, while Group 6 (Positive control) received 400 mg/kg body weight of metronidazole, 400 mg/kg body weight of vitamin E, and 0.36 mg/kg body weight of Sustanon. Sustanon (testosterone agonist) for the positive control treatment was administered once a week via intramuscular injection; all other treatments were given orally daily for 56 days.

### Sample collection and processing

At the end of the treatment period, the animals were deeply anaesthetized through intraperitoneal injection of 45 mg/kg sodium pentobarbital. The testis was dissected, fat trimmed off, washed in 1 × phosphate-buffered saline (PBS) (pH 7.4), separated from the epididymis, and then weighed. The left testis was immediately fixed in 10% phosphate-buffered formaldehyde (pH 7.4) for histological examination, while the right testis was stored at −20 °C for biochemical assays [11]. The left and right cauda epididymis were separated from the rest of the epididymis, weighed, and dissected for sperm retrieval and analysis. Sperm were collected according to the procedure described by Al-Alami *et al*. [11]. Briefly, the left and right cauda epididymis were placed in Petri dishes containing 10 mL of PBS buffer (pH 7.4) maintained at 37 °C and minced using a scalpel blade. The spermatozoa were allowed to diffuse into the buffer for 2 min and swirled to obtain a homogeneous suspension for sperm characteristic analysis. The sperm cell suspension was maintained in a pre-warmed water bath at 37 °C throughout the analysis.

### Evaluation of sperm characteristics

Sperm count was done according to the method described by Al-Alami *et al*. [11] and *WHO Laboratory Manual for the Examination and Processing of Human Semen* [29]. Briefly, an aliquot of 10 µL of the homogenous sperm suspension was loaded on each of the chambers of the haemocytometer covered with a coverslip and allowed to settle for 1 min before counting the sperm cells in the five large squares (four at the corners and the middle one) at ×100 total magnification under a light microscope (Olympus Microscope BH-2, USA). Sperm cells with their heads completely in the 5 medium squares or those lying on the top or left side of the square, but on the line, were counted (excluding those lying on the right or bottom sides of the middle large square). The counts were performed for the two counting chambers and averaged. The concentration of the spermatozoa in 1 mL of the suspension was calculated according to equation (2).


$$\begin{array}{c} Concentration\;(\frac{Count}{mL})=(Dilution\;factor)\times (Count\;in\;\text{5}\;squares)\times (\text{0}.\text{0}\text{5}\times {\text{1}\text{0}}^{\text{6}}) \end{array}$$ (2)

Sperm viability was assessed based on staining of the sperm cells with eosin-nigrosine stain, according to the *WHO Laboratory Manual for the Examination and Processing of Human Semen* [29], whereby 50 µL of properly mixed sperm suspension was added to equal volumes of eosin Y-nigrosin stain (prepared according to the WHO manual), mixed, and allowed to stand for 30 seconds on a microscope slide. A cover slip was immediately placed, and 200 spermatozoa were evaluated for each sample at 1000X magnification under oil immersion. Deep pink, red, and dark-stained heads (dead) and unstained/bright heads (live) were counted and recorded.

Sperm morphology was assessed according to the protocol described by Kondracki *et al*. [30], where thin smears of the spermatozoa were made on a microscope slide, stained with 1% eosin-Y, and counterstained with 5% nigrosin. Spermatozoa (200 for each sample) were examined at 400X light microscope magnification. Head and tail defects were recorded and expressed as a percentage of the total count.

### Testicular tissue processing for histology

Testicular tissues were prepared for histological examination according to the protocol described by Slaoui et al. [31]. The formaldehyde-fixed testes samples were washed overnight in running water, followed by dehydration in increasing ethanol concentrations (50%, 70%, 80%, 90%, 95%, and 100%) at 1-h intervals. The tissues were then cleared with methyl benzoate for 1 h, followed by infiltration with molten paraffin wax at 65 °C for 30 min, and finally embedded in paraffin wax and solidified overnight. The wax-containing samples were then placed on a wooden block for blocking, after which 5 µM-thick tissue sections were cut using a Leica Weitzlar® rotary microtome (Leica Microsystems, Germany). The sections were mounted on a glass slide, deparaffinized in xylene, and rehydrated by treatment with decreasing ethanol concentrations (100%, 90%, 70%, 50%) and, finally, water. The rehydrated sections were then stained using Haematoxylin and counterstained with eosin, dried, and examined at 1000 × total magnification using Leica® DM 500 (Leica Microsystems, Germany) light microscope.

### Malondialdehyde levels as an oxidative stress marker in the testis

Lipid peroxidation was assessed by measuring malondialdehyde levels in the testis according to the protocol described by Chen & Gallie [32]. Briefly, 0.05 g of testis was weighed and homogenized in 1 mL of ice-cold 0.1% (w/v) Trichloroacetic acid (TCA) using a mortar and pestle. The mixture was transferred to an Eppendorf tube, 0.25 mL of 0.1% (w/v) TCA was added, and the mixture was centrifuged at 000 xg for 5 *min* at 4°C. Next, 0.25 mL of the supernatant was transferred into clean Eppendorf tubes and mixed with 0.5 mL of 20% (w/v) TCA containing 0.5% (w/v) Thiobarbituric Acid (TBA). The mixture was incubated in a water bath at 95 °C for 30 *min*, cooled on ice, and then centrifuged again at 4,000 x g for 10 min at 4 °C. Absorbance was measured at 532 nm and 600 nm using a UV-visible spectrophotometer (UVmini-1240, Shimadzu, Duisburg, Germany). Non-specific turbidity was corrected by subtracting the absorbance at 600 nm from that at 532 nm. Malondialdehyde concentration was calculated using its molar extinction coefficient of 155 mM^-1 cm^-1. The results were expressed in nmol of Malondialdehyde per gram of fresh tissue, using the appropriate equation (3)


$$\begin{array}{c} MDA\;Content\;=Vol.\;of\;Extraction\;Buffer\;x\;Vol.\;of\;Supernatant\;(ml)\frac{\left[\frac{Abs\;\text{5}\text{3}\text{2}-Abs\;\text{6}\text{0}\text{0}}{\text{1}\text{5}\text{5}}\right]\text{1}\text{0}\text{0}\text{0}}{\left(Quantity\;of\;Sample\right)} \end{array}$$ (3)

Where 600 nm is the wavelength for correcting non-specific turbidity, while 532 nm is the maximum absorbance wavelength of the TBA-MDA complex.

All measurements from the testis tissue homogenate were performed in triplicate.

### Measurement of enzymatic antioxidants in the testis

Each testis sample (0.2 g) was homogenized on ice in 1.2 mL of 100 mM potassium phosphate buffer (pH 6.8), 0.2 mM EDTA, and polyvinylpyrrolidone (PVP) using a mortar and pestle. The mixture was then centrifuged at 5,000 xg for 15 min at 4°C. The supernatant was used for assaying catalase and peroxidase enzyme activities.

Catalase (CAT) activity was measured following the protocol by Lall *et al*. [33]. In a reaction tube, 1 mL of 50 mM PBS (pH 7.2) and 1 mL of 20 mM hydrogen peroxide (H2O2) were combined. The reaction was initiated by adding 50 µL of crude enzyme extract, and the reduction in absorbance due to H2O2 hydrolysis was monitored for 2 min at 240 nm. CAT activity was calculated using equation (4).


$$\begin{array}{c} Enzyme\;activity\;(Units/L)=\frac{\left(\Delta Abs\;\times \;Total\;assay\;volume\right)}{\left(\Delta t\;x\;\varepsilon \;x\;l\;x\;Enzyme\;sample\;volume\right)} \end{array}$$ (4)

Where: Δt is the time of incubation (min), ΔAbs is the change in absorbance, and ε is the

Molar extinction coefficient of substrates in units of M^-1^ cm^-1^, and l is the cuvette diameter (1 cm).

Enzyme activity (Unit) was defined as the amount of enzyme that oxidized 1μmol of substrate/min.

Peroxidase (POD) activity was conducted following the protocol described by Lall *et al* [33]. The assay was started by mixing 50 µL of tissue homogenate with 2 mL of a solution containing 15 mM guaiacol, 20 mM hydrogen peroxide, and 50 mM sodium acetate buffer (pH 7.0). The activity was measured by monitoring the increase in absorbance at 470 nm due to the formation of tetra guaiacol. The molar extinction coefficient of tetra guaiacol (26.6 mM–1 cm–1) was used to calculate the activity. Peroxidase enzyme activity was computed using equation (iv).

Enzyme activity (Unit) was defined as the amount of enzyme that oxidized 1 μmol of substrate/min.

### Statistical analysis

Data analysis was performed using R version 4.3.1 [34] and SigmaPlot version 15 [35]. Numeric data obtained from physiological and biochemical assays were subjected to one-way ANOVA, and multiple mean comparisons were done using Tukey’s Honest Significant Difference (HSD) post hoc test. Data is presented as Mean ± Standard Error of Mean (SEM). The results on mean difference were considered significant at P < 0.05.

## Results

### Molecular identification of the plant sample

The obtained PCR amplicon sizes following PCR amplification were approximately 600 base pairs (bp) with *rbcL* and 500 bp with *trnH-psbA* barcode (S1 Fig). A BLASTn search with the *rbcL* sequences in the database identified our query sequence with *Carissa haematocarpa* (JQ014161.1), *Carissa bispinosa* (AJ419738.1), and *Carissa bispinosa* (JQ014160.1), with percent identities of 99.7%, 99.7%, and 99.56%, respectively. While *trnH-psbA* identified a query sequence from the *Carissa spinarum* species, with percent identities ranging from 95.15% to 99.23% (S1 Fig).

A Bayesian phylogenetic inference approach was employed to further understand genetic identity, considering clustering based on sequence similarities. For the *rbcl* sequences (indicated by blue colour), the query sequence clustered with *Carissa bispinosa* (AJ419738.1), supported by a low posterior probability value of 69%. Based on the *trnH-psbA* barcode (indicated by red colour), the query sequence clustered with the three reference *C. spinarum* species (KR735499.1, KR735438.1, KR735402.1) with a posterior probability value of 99%, thus being identified as *Carissa spinarum* (S2 Fig).

### Phytochemical analysis of *C. spinarum* leaf and root extracts

Total Phenolic Content (TPC) in *C. spinarum* root extracts was significantly highest in acetone (130.39 ± 4.45 µg GAE/g), with other polar solvents (ethanol, methanol, Hydromethanol (H.M), Hydroethanol (H.E), distilled water, and hot distilled water) also extracting high phenolics. Hexane extracted a significantly lower concentration of phenolics (36.95 ± 0.16 µg GAE/g) (p < 0.05). TPC in the leaves showed a similar solvent pattern as for the root, with acetone yielding the highest (174.86 ± 1.73 µg GAE/g) and hexane the lowest (19.03 ± 0.31 µg GAE/g). Acetone also produced the highest amounts of Total Flavonoid Content (TFC) in root samples (0.98 ± 0.01 mg RE/g), while Hydromethanol (70%) showed the lowest TFC (0.02 ± 0.00 mg RE/g) (p < 0.05). In leaf samples, TFC was significantly higher in all polar solvents (Table 1).

**Table 1:** Summary of TPC, TFC, TTC, and TAC in *C. spinarum* roots and leaves in different extraction solvents.

Solvent of extraction	Total Phenol Concentration (µg Gallic Acid Equivalents/g Dry plant material)		Total Alkaloid Content (mg atropine equivalents/g of dry plant material)		Total Flavonoid Content (mg Rutin Equivalents/g Dry plant material)		Total Tannin Content (mg Tannic Acid Equivalents/g of Dry Plant Material)	
	*Roots*	*Leaves*	*Root extracts*	*Leaf Extracts*	*Roots*	*Leaves*	*Roots*	*Leaves*
Ethanol	109.24 ± 0.93^**b**^	157.57 ± 1.29^**b**^	0.147 ± 0.002^**b**^	0.235 ± 0.002^**a**^	0.51 ± 0.01^**b**^	2.84 ± 0.02^**ab**^	0.177 ± 0.01^**ab**^	0.254 ± 0.02^**b**^
Methanol	114.45 ± 1.17^**b**^	165.91 ± 1.27^**ab**^	0.128 ± 0.000^**c**^	0.220 ± 0.000^**b**^	0.90 ± 0.01^**a**^	2.87 ± 0.01^a^	0.191 ± 0.01^**ab**^	0.502 ± 0.01^**a**^
H.M (70% v/v)	107.99 ± 1.03^**b**^	147.16 ± 0.55^**c**^	0.024 ± 0.000^**d**^	0.034 ± 0.000^**d**^	0.02 ± 0.00^**d**^	2.71 ± 0.02^**ab**^	0.073 ± 0.01 cd	0.056 ± 0.02^**d**^
H.E (70% v/v)	109.86 ± 0.30^**b**^	147.78 ± 1.88^**c**^	0.013 ± 0.000^**e**^	0.020 ± 0.000^**e**^	0.08 ± 0.01^**d**^	2.49 ± 0.05^**ab**^	0.145 ± 0.00^**ab**^	0.202 ± 0.00^**bc**^
n-Hexane	36.95 ± 0.16^**c**^	19.03 ± 0.31^**e**^	0.011 ± 0.000^**e**^	0.014 ± 0.000^**e**^	0.24 ± 0.04^**c**^	1.19 ± 0.07^**c**^	0.012 ± 0.00^**d**^	0.027 ± 0.00^**d**^
D.W	113.82 ± 0.26^**b**^	141.74 ± 0.39^**c**^	0.009 ± 0.000^**e**^	0.015 ± 0.000^**e**^	0.37 ± 0.01^**bc**^	2.47 ± 0.02^**b**^	0.130 ± 0.01^**bc**^	0.209 ± 0.00^**bc**^
Acetone	130.39 ± 4.45^**a**^	174.86 ± 1.73^**a**^	0.026 ± 0.000^**d**^	0.231 ± 0.001^**a**^	0.98 ± 0.01^**a**^	2.59 ± 0.08^**ab**^	0.128 ± 0.02^**bc**^	0.174 ± 0.00^**c**^
D.W^*^	114.14 ± 0.33^**b**^	114.24 ± 0.48^**d**^	0.172 ± 0.000^**a**^	0.151 ± 0.001^**c**^	0.50 ± 0.02^**b**^	2.86 ± 0.01^**a**^	0.205 ± 0.01^**a**^	0.218 ± 0.00^**bc**^

Data are presented as mean ± Standard Error of the Mean (SEM). Statistical differences are indicated with the superscript letters (a, b, c, d, and e), where values sharing the same superscript letters within a column are not significantly different (p > 0.05). D.W = Distilled water; D.W* = distilled water (hot maceration method used); H.E = Hydroethanol (70% volume/volume); H.M = Hydromethanol (70% volume/volume). Note that ethanol, acetone, methanol, and distilled water generally extract high concentrations of phenols, flavonoids, tannins, and alkaloids from both roots and leaves.

The aqueous (hot maceration) extracts showed the highest tannin concentration in *C. spinarum* roots (0.205 ± 0.01 mg TAE/g), with hexane recording significantly lower tannin level in roots (0.012 ± 0.00 mg TAE/g) (p < 0.05). In the leaves, methanolic extracts had the highest tannin content (0.502 ± 0.01 mg TAE/g), while hexane had the lowest Total Tannin Content (TTC) (0.027 ± 0.00 mg TAE/g) (p < 0.05). The alkaloid concentration in root samples was highest in distilled water (hot) (0.172 ± 0.00 mg AE/g), while roots macerated with cold distilled water had the lowest alkaloid levels (0.009 ± 0.00 mg AE/g). On the leaves, ethanol extracted significantly higher alkaloids (0.235 ± 0.00 mg AE/g) (p < 0.05), with hexane showing the lowest alkaloid content in the leaves (0.014 ± 0.00 mg AE/g) (Table 1, S5 and S6 Fig).

### Comparison of phytochemical concentrations in *C. spinarum* leaf and root extracts

The results showed that total phenolic content, flavonoids, tannins, and alkaloids were significantly higher in the leaves compared to the roots in the following solvents: acetone, cold distilled water, Hydroethanol, Hydromethanol, ethanol, and methanol (p < 0.05) (S3 Fig).

### *In vitro* antioxidant activity of *C. spinarum* root and leaf extracts

In the root samples, ferric reducing power was significantly higher in Hydroethanol (6.66 ± 0.02), with hexane showing the lowest ferric reducing power (3.15 ± 0.02), Table 2. For the leaf extracts, Hydromethanol had the highest ferric-reducing capacity (10.88 ± 0.07), while hexane showed the lowest ferric-reducing capacity (p < 0.05), Table 2. The results for hydrogen peroxide (H_2_O_2_) scavenging activity in the root samples indicated that the acetone extract had significantly higher scavenging activity (34.18 ± 0.26), and hexane had the lowest H_2_O_2_ scavenging activity (9.70 ± 0.35) (p < 0.05). On the leaf extracts, ethanol demonstrated the highest H_2_O_2_ scavenging activity (43.53 ± 0.50), whereas hexane exhibited the lowest (18.54 ± 0.28), (p < 0.05) (Table 2, S5 Fig). Pairwise comparison of ferric reducing power between the leaf and root extracts showed that ethanol, methanol, Hydromethanol, Hydroethanol, acetone, cold distilled water, and hot distilled water had significantly higher ferric antioxidant reducing capacity in the leaf samples compared to root samples (p < 0.05). However, there was no significant difference in ferric reducing power in hexane extracts (S4 Fig). H_2_O_2_ scavenging activity was significantly higher in the leaf samples among the following solvents of extraction: ethanol, methanol, Hydroethanol, Hydromethanol, and acetone. The roots in hot distilled water showed significantly higher H2O2 scavenging activity compared to the leaves (p < 0.05) (S4 Fig).

**Table 2:** Effects of *C. spinarum* extracts on FRAP and HPRS activities.

Ferric Reducing Antioxidant Power (FRAP) (×10^−1^)			Hydrogen Peroxide Scavenging Activity (%)	
*Solvent of Extraction*	*Roots*	*Leaves*	*Roots*	*Leaves*
Ethanol	5.02 ± 0.01^**de**^	9.82 ± 0.09^**c**^	27.02 ± 0.21^**d**^	43.53 ± 0.50^**b**^
Methanol	5.08 ± 0.02^**de**^	9.82 ± 0.09^**c**^	30.44 ± 0.14^**c**^	45.78 ± 0.17^**b**^
Hydro-methanol	5.65 ± 0.03^**c**^	10.88 ± 0.07^**b**^	15.17 ± 0.27^**f**^	27.78 ± 0.38^**d**^
Hydro-ethanol	6.66 ± 0.02^**b**^	10.13 ± 0.16^**bc**^	15.70 ± 0.26^**f**^	19.79 ± 0.49^**e**^
Hexane	3.15 ± 0.02^**f**^	3.13 ± 0.03^**f**^	9.70 ± 0.35^**g**^	18.54 ± 0.28^**e**^
Distilled Water	5.47 ± 0.10 cd	6.81 ± 0.07^**de**^	22.35 ± 0.41^**e**^	19.59 ± 0.57^**e**^
Acetone	4.78 ± 0.13^**e**^	7.14 ± 0.09^**d**^	34.18 ± 0.26^**b**^	36.74 ± 0.30^**c**^
Distilled water^*^	5.40 ± 0.05 cd	6.12 ± 0.06^**e**^	30.80 ± 0.20^**c**^	30.90 ± 0.81^**d**^
Vitamin C	11.85 ± 0.08^**a**^	11.85 ± 0.08^**a**^	77.11 ± 0.46^**c**^	77.11 ± 0.46^**a**^

Data shown as mean ± standard error of mean (SEM), Statistical differences are indicated with the superscript letters (a, b, c, d, and e), where values sharing the same superscript letters within a column are not significantly different (p > 0.05). Cold maceration was used except for (Distilled water*), where hot maceration was the method employed for extraction.

### Correlation analysis and principle component analysis to determine effective solvents

In the root samples, TPC, TFC, TAC, and TTC had a strong positive correlation with HPRS (p < 0.05), while only TPC and TTC showed a strong positive correlation with FRAP. Alkaloid concentration showed a weak positive correlation with FRAP (P > 0.05). Leaf extracts showed that all metabolite concentrations were positively correlated with both FRAP and HPRS (S3 Fig). For the root and leaf extracts, a similar pattern was observed, with hot distilled water, ethanol, methanol, and acetone clustering together with at least one of the variable. H.E and H.M showed a positive correlation; however, they were not closely associated with any of the variables, whereas hexane was an outlier (S7 Fig).

### GC-MS results of *C. spinarum* leaf and roots, extracted with acetone and ethanol

**Fig 1:**
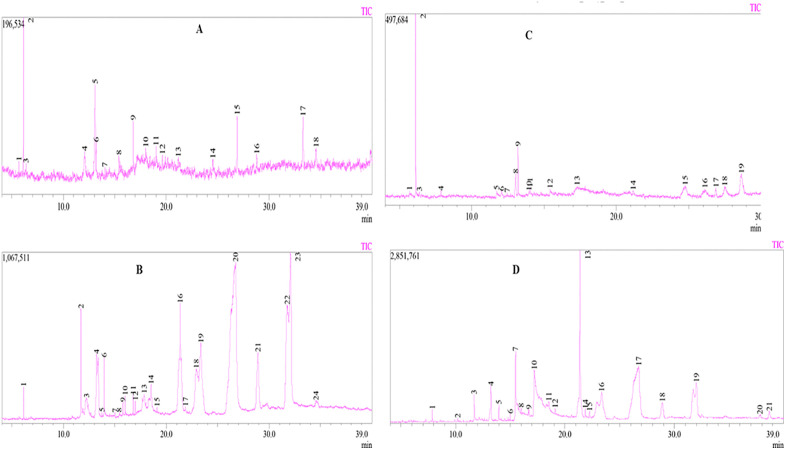
GC – MS Spectrograms, showing peaks of A (acetone leaf extract), B (ethanol leaf extract), C (acetone root extract), and D (ethanol root extract). The peaks are annotated with their respective numbers.

**Fig 2:**
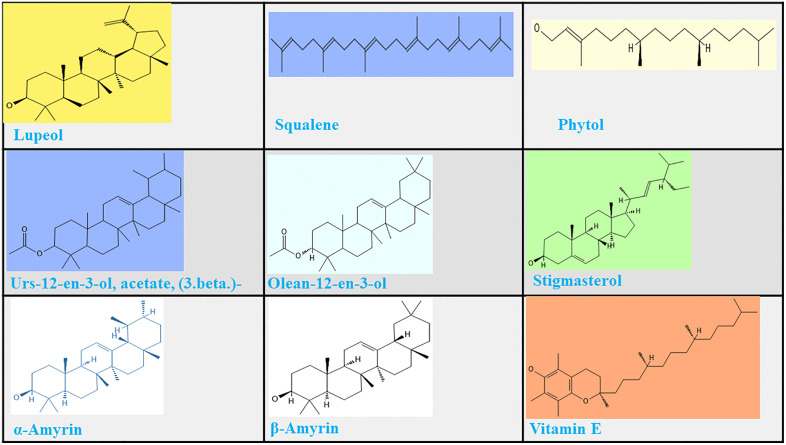
Sketches of pharmacologically relevant GC-MS-detected compounds. These compounds have been reported to reduce oxidative stress and improve male reproductive function. The sketches were made using PubChem Sketcher V2.4.

A total of 24 and 18 volatile compounds were detected in ethanolic and acetonic leaf extracts, respectively (Fig 1A and B). In the root extracts, the ethanolic and acetonic extracts contained 21 and 19 compounds, respectively (Fig 1C and D). Compounds with previous reports of antioxidant activity and male fertility modulation included, squalene [36], lupeol [37], phytol [38], Urs-12-en-3-ol, acetate, (3.beta.)- [39], Olean-12-en-3-ol [40], stigmasterol [41], α-Amyrin/ β-Amyrin [42] and Vitamin E [43,44] (S1-S3 Table and Fig 2).

### Mineral content analysis in *C. spinarum* leaf and roots

Among the analyzed macrominerals, calcium had the highest abundance in only the roots (57.08 ± 0.146), while potassium reported significantly high concentrations (ppm) in both leaf and root samples (50.01 ± 0.003 and 217.25 ± 0.536, respectively) (p < 0.05). Sodium had the lowest concentrations (ppm) in both root and leaf samples (2.02 ± 0.019 and 1.89 ± 0.049, respectively) (p < 0.05), as shown in Table 3. For trace elements, silicon recorded the highest concentrations in both leaf and root samples (57.45 ± 0.322 and 23.95 ± 3.481, respectively). Conversely, chromium (0.02 ± 0.003) had the lowest concentrations in root and leaf samples (p < 0.05), Table 3. Boron, a non-essential mineral, predominantly exhibited high concentrations in both leaf and root samples, while barium showed the lowest in both leaf and root samples (p < 0.05). A pairwise comparison of each analyzed mineral revealed that the leaves contained significantly higher concentrations of K, Mg, Cu, Zn, P, S, Si, Mn, Fe, B, and Ba, as shown in Table 3.

**Table 3:** Mineral content of *C. spinarum* leaf and root samples.

Mineral	Concentration in *C. spinarum* roots (ppm)	Concentration in *C. spinarum* leaves (ppm)	Root and leaf pairwise comparison (p-values)
**Macrominerals**			
Ca	57.08 ± 0.146^**a**^	18.93 ± 0.493^**d**^	0.00064
K	50.01 ± 0.003^**a**^	217.25 ± 0.536^**a**^	0.00029
Mg	32.53 ± 2.234^**b**^	75.37 ± 1.410^**b**^	0.00289
S	8.28 ± 0.271^**c**^	13.03 ± 0.443^**d**^	0.00710
P	5.40 ± 0.224^**d**^	26.77 ± 0.378^**c**^	0.00079
Na	2.02 ± 0.019^**d**^	1.89 ± 0.049^e^	0.14277
**Trace elements**			
Se	16.74 ± 0.148^**a**^	9.86 ± 0.150^**b**^	0.00249
Si	23.95 ± 3.481^**a**^	57.45 ± 0.322^**a**^	0.02569
Fe	3.58 ± 0.395^**b**^	9.46 ± 0.099^**b**^	0.01492
Zn	1.0 ± 0.139^**c**^	1.15 ± 0.152^**d**^	0.71568
Mn	0.50 ± 0.026^**c**^	5.71 ± 0.322^**c**^	0.01197
Cu	0.13 ± 0.008^**c**^	0.19 ± 0.014^**d**^	0.03100
Mo	0.03 ± 0.002^**c**^	0.04 ± 0.011^**d**^	0.78918
Cr	0.02 ± 0.003^**c**^	0.13 ± 0.017^**d**^	0.05336
**Non-Essential Minerals**			
B	0.18 ± 0.01^**a**^	1.64 ± 0.013^**a**^	0.00033
Ba	0.05 ± 0.01^**b**^	0.35 ± 0.017^**b**^	0.00729

Data are presented as mean ± standard error of mean (SEM). Statistical differences are indicated with the superscript letters (a, b, c, d, and e). For each mineral category (macrominerals, trace elements, and non-essential minerals), the concentration values on leaves and roots with the same superscripts are not significantly different at the 5% significance level. Mineral concentration was measured in parts per million (ppm); n = 3.

### Effects of *C. spinarum* aqueous root extract on reproductive organ and body weights

The testicular weight index assessment showed a significant decrease in the negative control group receiving metronidazole alone (p < 0.05). In contrast, the extract-treated groups, especially those given low and medium doses, showed a significant increase in relative testis weight index (0.95% and 0.98%, respectively) compared to the negative control (0.28%; p < 0.05). The relative testicular weights in the low and medium extract-treated groups were not significantly different from those in the normal control group (1.015%; p > 0.05). Similarly, cauda epididymis weights were significantly reduced among the negative control group (0.28g; p < 0.05). Conversely, the low and high-dose extract-treated groups exhibited increased epididymal weights (0.45g and 0.45 g, respectively), which were not significantly different from the positive and normal control groups (0.52g and 0.55 g, respectively) (p > 0.05) (Table 4, S9 Fig).

**Table 4:** Summary table indicating the effects of *C. spinarum* aqueous root extract on total sperm count, viability, morphology, testis weight index, and epididymis weight.

Groups	n	Sperm Count (×10^6^) per mL of the suspension.	Viable Sperm cells (%)	Spermatozoa with normal Morphology (%)	Testis Weight Index (%)	Total epididymal Weight (g)	Malondialdehyde (MDA) (nmol/g of tissue sample	Catalase enzyme Activity (nmol g^-1^ testis tissue) × 10^−2^	Peroxidase Enzyme Activity (nmol g^-1^ testis tissue) × 10^−2^
1	5	55.00 ± 1.21^**ab**^	86.60 ± 1.43^**a**^	85.50 ± 1.23^**a**^	1.015 ± 0.01^**a**^	0.55 ± 0.02^**a**^	0.88 ± 0.08^**c**^	15.19 ± 0.09^**a**^	19.87 ± 0.13^**b**^
2	5	3.49 ± 0.46^**d**^	68.60 ± 0.54^b^	4.30 ± 0.92^**d**^	0.45 ± 0.01^**b**^	0.28 ± 0.01^**c**^	5.35 ± 0.06^**a**^	8.45 ± 0.09^**b**^	17.26 ± 0.11^**b**^
3	5	35.03 ± 2.91^**bc**^	88.50 ± 1.62^**a**^	41.90 ± 4.77^**bc**^	0.95 ± 0.10^a^	0.45 ± 0.02^**ab**^	3.89 ± 0.50^**ab**^	13.88 ± 0.21^**a**^	28.07 ± 0.24^**ab**^
4	5	37.20 ± 3.86^**abc**^	86.70 ± 1.42^**a**^	60.50 ± 3.09^**ab**^	0.98 ± 0.02^**a**^	0.39 ± 0.01^**bc**^	1.38 ± 0.14^**c**^	14.56 ± 0.19^**a**^	30.95 ± 0.11^**ab**^
5	5	11.66 ± 1.62^cd^	70.50 ± 2.90^**b**^	32.60 ± 3.83^**c**^	0.78 ± 0.04^**ab**^	0.43 ± 0.02^**ab**^	2.77 ± 0.23^**bc**^	10.79 ± 0.22^**ab**^	33.24 ± 0.14^**ab**^
6	5	64.43 ± 4.82^**a**^	88.20 ± 1.18^**a**^	83.20 ± 1.49^**a**^	0.70 ± 0.02^**ab**^	0.52 ± 0.01^**ab**^	1.03 ± 0.19^**c**^	13.30 ± 0.18^**a**^	38.52 ± 0.10**a**

Effect of *C. spinarum* root extract on sperm parameters and biochemical antioxidant markers in rats after 58 days of treatment. Note that at low and medium extract doses, there was an improvement in sperm characteristics, increased testicular weight index, cauda epididymis weights, and selected biochemical antioxidant markers compared to the negative control. (G1 = Distilled water, G2 = Metronidazole 400 mg/kg bw, G3 = 400 mg/kg bw Metronidazole + 250 mg/kg bw extracts, G4 = 400 mg/kg bw Metronidazole + 500 mg/kg bw extracts, G5 = 400 mg/kg bw Metronidazole + 1000 mg/kg bw extracts, G6 = 400 mg/kg bw Metronidazole + 400 mg/kg bw Vitamin E + 0.36 mg/kg bw Sustanon weekly injection). Data are presented as Mean ± (Standard Error of Mean) SEM; Statistical differences are indicated with the superscript letters (a, b, c, d, and e), where values sharing the same superscript letters within a column are not significantly different (p > 0.05). n = number of animals, nmol represents nanomoles

### Effects of *C. spinarum* aqueous root extract on sperm qualities

The administration of metronidazole alone in the negative control (group 2) led to a significant decrease in sperm concentration 3.49 × 10^6^/mL) and viability 68.6%, and consequently lowered the levels of morphologically normal sperm cells to only 4.3%. However, treatment with escalating doses of the root extracts significantly (p < 0.05) improved these parameters especially on the low and medium doses of the extracts where sperm concentration was 35.03 × 10^6^/mL and 37.2 × 10^6^/mL respectively, viability 88.5% and 86.7% respectively, while the amounts of sperms with normal morphology were high in these groups (41.90% and 60.5%, respectively). The medium extract dose showed sperm parameters that were not significantly (p > 0.05) different from those of the normal and positive controls. At a high dose of the root extracts, there was a significant reduction (p < 0.05) in sperm parameters compared to all other treatment groups, including the positive and normal controls, but not significantly different from the negative control (Table 4, S10 Fig, and S11 Fig).

### Testicular histology

**Fig 3:**
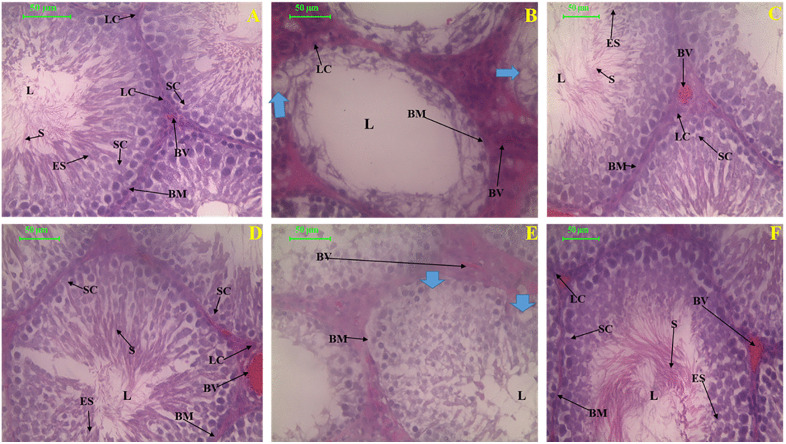
Photomicrographs of rat seminiferous tubules. **A**) Normal control with normally aligned spermatogenic cells at different stages of development interspersed among Sertoli cells (SC) lying on intact basement membrane (BM) and whose apical villi attach the elongate spermatids (ES) and appear to project into the lumen (L). **B**) Negative control subjected to metronidazole alone showing significantly disorganized seminiferous epithelial alignment within the seminiferous tubules with accompanying increased luminal diameter (L) that appears empty. Vacuolations can also be seen (thick blue arrow), **C** and **D**) represent low and medium doses of *C. spinarum root* extracts, respectively. The seminiferous tubular integrity in both treatment groups appears intact, with the cellular ultrastructure resembling that of the normal control. **E**) represents the high dose extract-treated group. Note the loss of seminiferous epithelial integrity characterised by disintegration and general swelling of the seminiferous epithelial diameter. The basement membrane (BM) appears irregular, and the lumen (L) is empty. **F**) represents the positive control, its seminiferous tubular ultrastructure resembles that of the normal control with intact BM and spermatogenic cells at different stages of development interspersed among the Sertoli cells (SC) with spermatozoa (S) in the lumen. The interstitium of the positive control group also shows a cellular outline comprising Leydig cells (LC) among other interstitial cells and blood vessels (BV). Haematoxylin and eosin stain used and the magnification bar represents 50 µm.

In the normal control group, the testicular parenchyma consisted of regularly shaped seminiferous tubules, which appeared bounded by an intact basement membrane supporting spermatogonia and Sertoli cells. The Sertoli cells are tall and seem to span the entire diameter of the seminiferous epithelium, with apical villi extending into the lumen, while lateral recesses support and contain dividing and differentiating germ cells. The tubular lumen contained spermatozoa with tails pointing toward the center of the lumen and heads attached to the apical villi of the Sertoli cells. The spermatogenic cells appeared to develop in groups at the same stage toward the lumen, indicating a normal spermatogenic cycle and wave. The interstitial tissue of the normal control displayed interstitial cells, blood vessels, and lymphatic vessels. Among these interstitial cells are Leydig cells, which appear polyhedral (Fig 3A).

The negative control, which was treated with metronidazole 400 mg/kg/bw alone, showed significant structural changes in the seminiferous tubules. The seminiferous epithelium generally appeared thinner with prominent vacuolation in spermatogenic cells and a lack of Sertoli cells. There was evidence of complete seminiferous tubular degeneration, with dissociation from the basement membrane, which appeared irregular and showed cell exfoliation in some areas. The interstitial tissue appeared abnormally enlarged, with signs of edema and irregular distribution of interstitial cells (Fig 3B).

Seminiferous tubules in animals treated with low and medium doses of extract exhibited normal structural features, including an intact germinal epithelium and basement membrane. The germinal epithelium within the seminiferous tubules was characterized by well-organized spermatogenic cells at various stages of development, intermingled with Sertoli cells and spermatozoa (Fig 3C and D).

At high extract dose treatment, the basement membrane of seminiferous tubules appeared irregularly shaped with folds in certain areas (Fig 3C and D), and the seminiferous epithelium seemed dissociated from the basement membrane and from each other. There was evidence of cellular degeneration, as indicated by pyknotic nuclei in the affected cells. The seminiferous epithelial cells were also exfoliated from the basement membrane (Fig 3E).

The seminiferous tubules in the positive control group exhibited features of seminiferous epithelium resting on an intact basement membrane, with a lumen filled with spermatozoa, indicative of a normal seminiferous tubule (Fig 3F).

### Effects of *C. spinarum* aqueous root extract on oxidative stress markers and enzymatic antioxidants in the testis

To understand the *in vivo* antioxidant properties of aqueous root extracts of this plant in relation to male infertility treatment, testicular levels of malondialdehyde (MDA) and the activity of peroxidase and catalase were measured in right testis homogenates. The negative control group, which was treated with metronidazole alone, showed the highest concentration of MDA (5.35 ± 0.69 nanomoles/g of testicular tissue) (p < 0.05). The treatment groups, especially those given medium (500 mg/kg) and high (1000 mg/kg/bw) extract doses, exhibited significantly lower MDA levels of 1.38 ± 0.14 and 2.77 ± 0.23 nanomoles/g of testicular tissue (p < 0.05). The animals treated with medium and high extract doses showed MDA levels that were not significantly different from those in the positive and normal control groups (p > 0.05) (Table 4).

The results for catalase enzyme activity showed a significant reduction among the negative control group (8.45 ± 0.09 nmol g^-1^ testis tissue × 10^−2^) treated with metronidazole alone. However, treatment with low and medium doses led to a significant (p < 0.05) increase in the levels of catalase enzyme activity of 13.88 ± 0.21 and 14.56 ± 0.19 nmol g^-1^ testis tissue × 10^−2^, respectively. The results also indicated that those MDA levels from the low and medium extract-treated animals were not significantly different from the normal and positive control group animals (p > 0.05) (Table 4).

The test for peroxidase enzyme activity reported normal levels of the enzyme activity in the negative control animals (17.26 ± 0.11 nmol g^-1^ testis tissue × 10^−2^) with reference to the normal control group (p > 0.05). However, there was an increase in the activity of peroxidase enzyme activity among the low (28.07 ± 0.24 nmol g^-1^ testis tissue × 10^−2^), medium (30.95 ± 0.11 nmol g^-1^ testis tissue × 10^−2^), and high (33.24 ± 0.14 nmol g^-1^ testis tissue × 10^−2^) extract-treated groups. This increase in peroxidase enzyme activity among the extract-treated groups was not significantly different with reference to the positive control animals (p > 0.05) (Table 4).

## Discussion

Plants within the same family exhibit similar morphological features, calling for DNA barcoding in discrimination due to its robustness and high level of accuracy. In this study, trnH*-psbA* authenticated the identity of the plant species as *Carissa spinarum* L. following BLASTn search and phylogenetic analysis. The *trnH-psbA* barcode exhibited clustering of the query sequence with other database sequences belonging to the *Carissa spinarum* species, supported with a high posterior probability of 99%, leading to the agreement on the plant species to be *C. spinarum*. Similar results supporting the accuracy and efficiency of *trnH-psbA* in species discrimination were also reported by Kress *et al*. [45].

It was evident that acetone, hot distilled water, methanol, and ethanol generally extracted higher amounts of phenolics, flavonoids, alkaloids, and tannins in both leaf and root samples, confirming their presence in *C. spinarum* leaf and root samples in high amounts. The present study, therefore, gives insights into the availability of highly soluble phenols, flavonoids, alkaloids, and tannins in *C. spinarum* root and leaf samples. These compounds have been shown to exhibit various pharmacological actions and therefore support the previous findings on the use of *C. spinarum* in the treatment of various diseases such as chest pains, viral infections, male infertility, malaria, and inflammatory diseases [46]. A comparison of the leaf and roots revealed generally high phenol, flavonoid, tannins, and alkaloids content in the leaf extracts in most solvents compared to the roots. In most vegetative plants, phenols, flavonoids, alkaloids and tannins are normally synthesized and concentrated in the leaves, suiting their roles in protection against ultraviolet radiations, defence against pathogens, parasites, and herbivorous animals [47]. The ferric-reducing antioxidant power and hydrogen radical-scavenging capacity of *C. spinarum* root and leaf extracts were high in methanol, ethanol, acetone, and hot distilled water, suggesting high solubility of the antioxidant compounds present in *C. spinarum* in polar organic solvents and in hot distilled water. The potent antioxidant activity and diverse phytocompounds exhibited by *C. spinarum* support its effectiveness in treating oxidative stress-related pathologies, inflammatory diseases, and microbial infections [46].

Pearson’s correlational analysis and principle component analysis (PCA) were utilized to scrutinize the influence of phytocompound concentration on antioxidant activity and the most effective extraction solvents, respectively. In the root extracts, phenols, tannins, and alkaloids contributed positively and were key determinants of FRAP and HPRS. Antioxidant activities in the leaf extracts were contributed to by all the secondary metabolites assayed. This is consistent with previous findings on the positive contribution of different classes of SM to radical scavenging [14,47]. PCA revealed acetone, methanol, ethanol, and hot distilled water as the most effective solvents, with a similar extraction pattern observed through clustering in both the roots and leaf samples of *C. spinarum*. This information is pivotal for selecting an appropriate solvent for extracting targeted groups of compounds and for assessing antioxidant activities.

Volatile compound detection indicated that the highest number of volatile compounds were present in ethanol, for both the leaf and root samples, suggesting the suitability of ethanol in dissolving most of the compounds in *C. spinarum.* Among the compounds detected previous studies have shown the bioactivity of squalene [36], lupeol [37], phytol [38], Urs-12-en-3-ol, acetate, (3.beta.)- [39], Olean-12-en-3-ol [40], stigmasterol [41], α-Amyrin/ β-Amyrin [42] and Vitamin E [43,44] as antioxidant and male reproductive function modulators, where they enhance spermatogenesis and protects the testicular tissues against oxidative stress based injuries. The findings from GC-MS are relevant in supporting the effects of *C. spinarum* plants in the inhibition of oxidative stress-mediated pathologies and management of male infertility.

Minerals play pivotal roles in health and are thus recommended for intake through dietary sources or direct supplementation. In the current study, results showed that both the leaves and roots were rich in Calcium, potassium, magnesium, sulfur, phosphorus, and sodium as macrominerals, and selenium, which are required by the body in milligram quantities per day. Potassium and sodium ions are essential for maintaining electrolyte balance, and magnesium is also required as a cofactor in more than 300 metabolic reactions. Calcium, phosphorus, and sulfur are needed for bone mineralization, Components of genetic information (DNA and RNA), and in cellular signalling, respectively [48]. The findings also showed that both leaf and root samples were rich in trace elements, including selenium, silicon, iron, zinc, manganese, molybdenum, and chromium. The body requires selenium, copper, iron, and zinc as antioxidants, components of cytochrome, oxygen transport, and enzymatic cofactors, respectively [48,49]. These minerals are therefore crucial for maintaining growth and development, boosting immunity, and supporting reproductive function in both males and females [48]. The leaves were found to contain a generally higher concentration of minerals than the roots; this may be linked to several biochemical and metabolic activities that are more likely to occur in the leaves than in the roots [48]. The availability of these minerals, along with other phytochemicals, can therefore support the plant’s previously demonstrated therapeutic effects in traditional and modern applications, including the treatment of oxidative damage-based pathologies [49,50].

*Carissa spinarum* has had a long history in ethnomedicine, with studies reporting its application in the management of male infertility [6–8]. In the present study, the counteractive effects of *C. spinarum* aqueous root extracts on the oxidative stress-induced infertility of metronidazole on the testis of adult male Wistar rats were investigated. The findings on reproductive organ weight revealed a remarkable reduction in testis weight index and cauda epididymis weight in the negative control groups treated with metronidazole alone, possibly due to previously reported necrotic and degenerative effects of Metronidazole-induced oxidative stress [5]. We also found that the co-administration of *C. spinarum* aqueous root extract ameliorated the effect of Metronidazole, and the tail epididymis and relative testis body weight were significantly increased and were similar to the positive and normal controls. These effects can be attributed to the protective capacity of the secondary metabolites present in the aqueous root extracts. Similar results were reported by Al-Alami et al. [11], who demonstrated the use of plant extracts to counter Metronidazole-induced changes in the testis of male Wistar rats.

Evaluation of sperm parameters showed a remarkable reduction in sperm concentration, viability, and morphological alterations in the negative control, which received metronidazole alone, might have been due to increased testicular cytotoxic activities of metronidazole [51]. This could either interfere with gene expression of testicular steroidogenic enzymes, synergize spermatogenesis, downregulate pituitary gonadotropin receptor binding on Sertoli cells, or directly affect sperm DNA via lipid peroxidation, or a combination of some or all of these factors, thereby impairing spermatogenesis. However, reversal of this measure was observed in the low- and medium-dose extract-treated groups, suggesting the potential to ameliorate testicular tissue damage owing to metronidazole-induced oxidative stress, mediated by the extract. Previous studies on phytochemical analysis of *C. spinarum* root extracts, its therapeutic benefits were ascribed to high antioxidant activity as a result of high concentration of phenols, tannins, flavonoids, and alkaloids [46]. These plant phytocompounds are also known to improve sperm characteristics, as well as to strike the balance between ROS production and scavenging potential, during oxidative stress [52]. Some reports have shown improvement of antioxidant, spermatogenic, and steroidogenic enzyme activities [11]. From the foregoing, it is possible that these compounds acted synergistically or individually to restore sperm count, morphology, and viability. In a similar study on fertile and infertile men, it was reported that secondary metabolites in medicinal plants improve fertility by boosting reproductive hormone action and improving various sperm characteristics, including morphology, vitality, concentration, and motility [53,54].

Testicular histology revealed seminiferous tubules with normal structural features at low and medium-dose treatment groups that were similar to the normal and positive controls. This was probably due to the ameliorative effects of *C. spinarum* on oxidative stress-mediated testicular tissue damage induced by metronidazole. Spermatogenesis depends heavily on the integrity of the testicular parenchyma, where key cells, such as interstitial endocrine cells, germ cells, and sustentacular cells within the seminiferous epithelium, play important roles [55]. Alteration of the germinal epithelium as well as the interstitial endocrine cells are known to impair spermatogenesis [56], as observed in the negative control and high dose extract group. Vacuoles on seminiferous tubular epithelium have been ascribed to impaired spermatogenesis as was reported by in rabbits treated with high doses of khat extracts [57] and vervet monkeys treated with high doses of (-)-cathinone [58].

The histological findings support the results from sperm characteristics and reproductive organ weights in this study. In low and medium-dose treatment groups, normal spermatogenesis was observed, and this explains the high number of spermatozoa, viability, and normal morphology as compared to the negative control. This also translated to higher cauda epididymal weights, since 50% of the tail epididymis weight is attributed to spermatozoa [11]. The observations from testicular histology also showed that the low- and medium-dose extract-treated groups had normal testicular contents. This might have accounted for the higher absolute testicular weights compared to the negative control, whose seminiferous tubules were largely void of cells that are part of the testicular content. The variance in measured sperm parameters among treatment groups and the positive control, despite similar morphological features, remains an area for further research. Perhaps more detailed ultrastructural studies may offer more information on cytological effects and this is an area for future studies.

Test on malondialdehyde concentration revealed high MDA level in negative control, confirming the contribution of metronidazole in increasing oxidative stress by enhancing lipid peroxidation. Reactive oxygen species enable the abstraction of hydrogen, replacing it with oxygen atoms and forming lipid peroxyl radicals and hydroperoxides. These molecules disrupt the membrane’s integrity and interact with other biomolecules, such as proteins, thereby disrupting physiological and biochemical processes essential to fertility [52], as seen in the negative control group of the present study. However, the reduction of radicals was observed in the low- and medium-dose extract-treated groups, confirming the extract’s *in vivo* antioxidant potential.

A test on antioxidant enzymes essential for the maintenance of the redox cycle revealed a significant reduction in catalase activity in the negative control group that received metronidazole alone. The cumulative deleterious effects of metronidazole on the testicular tissues might have resulted to this as previously reported by [51]. The increase in catalase enzyme activity in extract-treated groups, similar to the normal and positive controls, demonstrates the effects of *C. spinarum* aqueous root extract in alleviating catalase activity inhibition induced by metronidazole administration. However, the reversal groups with the effects of bioactive compounds in the extract may have mediated the restoration of catalase activity by augmenting the extract’s radical-scavenging activities. Activity of peroxidase enzyme was not altered upon administration of metronidazole alone with reference to the normal control animals. This suggests that metronidazole-induced oxidative damage does not have inhibitory effects on peroxidase enzyme activity. However, administration of the extract enhanced peroxidase activity among the treatment groups, similar to the positive control. This confirms the *in vivo* antioxidant contribution of extract’s bioactive compounds. This could also reveal possible similarity in mechanism, between *C. spinarum* aqueous root extracts and vitamin E, an antioxidant on increasing catalase and peroxidase activities, in restoring testicular tissue redox balance.

## Conclusions

The findings from this study confirmed that oxidative stress-mediated infertility in adult male Wister rats can be reversed using aqueous root extracts from *C. spinarum*. This was supported by improved sperm qualities and alleviation of oxidative stress in the animal groups treated with aqueous root extracts of *C. spinarum*. Additionally, the improvement of sperm characteristics and normal testicular morphology in the low and medium-dose extract-treated groups suggests activation of spermatogenic pathways, potentially due to enhanced testosterone hormone action. The finding therefore substantiates the potential of extrapolation of this plant’s herbal preparation in restoring male infertility attributed to ROS damage in humans, pending further investigations. From our knowledge, this is the first report demonstrating the ameliorative effects of *Carissa spinarum* on oxidative stress-induced male infertility. By isolating and screening single compounds, as well as investigating their specific molecular and biochemical pathways, we can understand intricate details of this plant’s extract’s mode of action in restoring male fertility. This will pave the way for the development of targeted therapeutic interventions.

## Supporting information

S1 Table Reference sequences that matched BLASTn Search of the query sequences. For *rbcl*, *Carissa haematocarpa (*JQ014161.1), and *Carissa bispinosa (*AJ419738.1) had the highest percent identity of 99.7% each. For *trnH-psbA*, *Carissa spinarum* of accessions KR735499.1 and KR735438.1 recorded the highest percent identity of 99.23 each.
(DOCX)

S2 Table Volatile compounds detected in *C. spinarum* leaves and roots extracted with acetone and ethanol. The table shows the compounds identified by the GC-MS, including Hexadecanoic acid, methyl ester, Estra-1,3,5(10)-trien-16-one, 3-[(trimethylsilyl)oxy]-, Androst-2-en-17-one, 4,4-dimethyl-, (5alpha.)-, Stigmasterol, Phytol, Vitamin E with previous antioxidant and previously reported male fertility enhancement properties. Column 2 is represented as the Solvent/peak number from the respective chromatogram and the retention time. Each of the rows in column 2 is matched with the respective compound peak area and part of the plant from which it was detected in columns 3 and 4, respectively.
(DOCX)

S3 Table Correlation coefficients of TPC, TFC, TTC, TRAC, and FRAP, HPRS. The strength and direction of the relationships between the three polyphenolic groups, alkaloids across all solvents and FRAP, HPRS activity based on Pearson’s correlation analysis. Correlations that were not statistically significant (p > 0.05) are noted with an asterisk.
(DOCX)

S1 Fig Agarose gel Electrophoresis of PCR amplicons DNA barcodes. Electrophoresis (1% (w/v) agarose) gel image of the PCR amplicons of rbcl, and trnH-psbA barcoded for identification. L is the 1kb plus DNA ladder Thermo Scientific™ GeneRuler, neg (negative control well).
(TIF)

S2 Fig Phylogram based on the rbcL and trnH-psbA barcode, showing the evolutionary relationship between *C. spinarum* (MOUONBI 2023/001) and other species retrieved from the database. The trnH-psbA barcode enabled species identification through clustering with similar species, supported by a 99% posterior probability. The tree was constructed using the Markov Chain Monte Carlo (MCMC) algorithm in Mr Bayes. The numbers at the nodes show the posterior probability, expressed as a percentage, and indicate the topological robustness of the phylogenetic tree.
(TIF)

S3 Fig Comparison of total phenolics (A), total flavonoids (B), total alkaloids (C), and total tannin content (D) in *C. spinarum* root and leaf extracts. Met (methanol), D.W = Distilled water, D.W* = distilled water (hot maceration method used), H.E = Hydroethanol, H.M = Hydromethanol. In most solvents, the leaves showed significantly higher concentrations of the phytochemicals assayed than the roots. Bars with asterisks are not significantly different (P > 0.05), following paired two-tailed t. test analysis. Met (methanol), D.W = Distilled water, D.W* = distilled water (hot maceration method used), H.E = Hydroethanol, H.M = Hydromethanol.
(TIF)

S4 Fig Comparison of Hydrogen peroxide radical scavenging activity, (HPRS) (A), and ferric reducing antioxidant power (FRAP) (B) and in *C. spinarum* root and leaf extracts. Met (methanol), D.W = Distilled water, D.W^*^ = distilled water (hot maceration method used), H.E = Hydroethanol, H.M = Hydromethanol. Bars with asterisks are not significantly different (P > 0.05), following paired two-tailed T. Test analysis.
(TIF)

S5 Fig Bar graphs showing the phenolics and flavonoids contents in *C. spinarum* root and leaf extracts, expressed as Gallic Acid Equivalents per gram of sample (GAE/g) and Rutin Equivalents per gram of sample (RE/g), respectively. Total phenolic content in root extracts (A) and leaf extracts (B), total flavonoid content in root extracts (C) and leaf extracts (D) by use of polar solvents. Note that ethanol, acetone, methanol and distilled water generally extracted high concentrations of flavonoids and phenols in both the roots and the leaves. Statistical differences are indicated with the letters (a, b, c, d, and e) on top of each bar, where those with a similar letter are not significantly different (p > 0.05). D.W = Distilled water, D.W* = distilled water (hot maceration), H.E = Hydroethanol, H.M = Hydromethanol, Met (Methanol).
(TIF)

S6 Fig Bar plots showing the concentration of Tannins and Alkaloids in *C. spinarum* leaf and root extracts expressed as Tannic Acid Equivalents per gram of sample (TAE/g) and Atropine equivalents per gram of sample (AE/g), respectively. Total tannin content in the root (A), and leaf extracts (B), total alkaloid in the root (C), and leaf extracts (D). Note that polar solvents, including ethanol, acetone, methanol and distilled water, generally extract high concentrations of flavonoids and phenols in both the roots and the leaves. Statistical differences are indicated with the letters (a, b, c, d, and e) on top of each bar, where those with a similar letter are not significantly different (p > 0.05). D.W = Distilled water, D.W^*^ = distilled water (hot maceration), H.E = Hydroethanol, H.M = Hydromethanol, Met (methanol).
(TIF)

S7 Fig Shows PCA biplots and scree plots for leaf (A & B, respectively) and root (C & D, respectively) extracts. The PCA biplot displays Dimensions 1 (Dim 1) and 2 (Dim 2), which represent the percentages of variation in the original data explained by PCA 1 and PCA 2, respectively. The dependent variables are shown by blue arrows based on eigenvectors and eigenvalues, and include TPC, TAC, TFC, TTC, FRAP, and HPRS. Individual observations are represented by the extraction solvents, shown as different-coloured shapes grouped accordingly. MeOH is methanol; D.W (Distilled Water used in cold maceration); Hot D.W (Distilled water used in Hot maceration); EtOH (ethanol); H.M (Hydromethanol, 70%); and H.M (Hydroethanol, 70%). The scree plots show the principal components and the percentage of variation each accounts for. Dim 1 and Dim 2 are principal components 1 and 2, respectively. The length of the arrows indicates the magnitude of variation contributed to the original data. The direction of the arrows indicates the type of correlation among variables: arrows pointing in the same direction, with an angle difference less than 90°, indicate a positive correlation and suggest similar trends.
(TIF)

S8 Fig Graphical representation of the increase in body weights of animals during the study. 2000 mg/kg bw was the group that administered the high dose of C. spinarum aqueous root extract, and the control received 1 mL of distilled water. Body weights were measured in grams from the initial day D0 to the 14^th^ day (D14). No treatment was done at day 0 (D0).
(TIF)

S9 Fig Effect of root extract of C. spinarum on relative testicular weights (A), epididymis (B), and body weight changes (C). All extract-treated groups showed a significant increase in relative testicular weights, epididymis weights, and percent body weight gain compared to the negative control group. Each bar graph represents different treatment groups of experimental animals (G1 = Distilled water, G2 = Metronidazole (400 mg/kg bw^-1^), G3 = 400 mg/kg bw Metronidazole + 250 mg/kg body weight extracts, G4 = 400 mg/kg bw Metronidazole + 500 mg/kg bw extracts, G5 = 400 mg/kg bw Metronidazole + 1000 mg/kg bw extracts, G6 = 400 mg/kg bw Metronidazole + 400 mg/kg bw Vitamin E + 0.36 mg/kg bw Sustanon weekly injection) which contained 5 animals. Body weight changes are the differences between day 0 and day 56, expressed as a percentage. The weight of the animals, testis and epididymis were measured in grams. Statistical differences are indicated with the letters (a, b,c, d, and e) on top of each bar, where those with a similar letter are not significantly different (p > 0.05).
(TIF)

S10 Fig micrographs showing different sperm characteristics assessed, following eosin-nigrosin staining, and examination under a light microscope at 400 × total magnification A represents viability status of the sperm cells, where the black arrows indicates dead sperm cells with dark stained heads, the blue arrows shows viable sperm cells with intact unstained heads B shows sperm cell with neck region defects, C represents sperm cell with normal morphological features while D shows sperm cell with an abnormal tail bending. (TIF)

S11 Fig Effect of the *C. spinarum* root extract on the sperm parameters in rats following 58 days of treatment. The graphs present total sperm count (**A**), Sperm Viability (**B**), and Morphology (**C**). Note that at low and medium extract doses, sperm characteristics improved compared to the negative control. Each bar graph represents different groups of animals (n = 5), (G1 = Distilled water, G2 = Metronidazole 400 mg/kg bw^-1^, G3 = 400 mg/kg bw Metronidazole + 250 mg/kg bw extracts, G4 = 400 mg/kg bw Metronidazole + 500 mg/kg bw extracts, G5 = 400 mg/kg bw Metronidazole + 1000 mg/kg bw extracts, G6 = 400 mg/kg bw Metronidazole + 400 mg/kg bw Vitamin E + 0.36 mg/kg bw Sustanon weekly injection). Statistical differences are indicated with the letters (a, b,c, d, and e) on top of each bar, where those with a similar letter are not significantly different (p > 0.05).
(TIF)

S1 File Raw Images. (PDF)

S2 File Raw data. (XLSX)
