# Supplementary material for: Carissa spinarum L. aqueous root extracts ameliorate oxidative stress-induced infertility in adult male Wistar rats
Source: PLoS One. 2026 Aug 11;21(8):e0355380. doi: 10.1371/journal.pone.0355380 (PMC13460572; doi:10.1371/journal.pone.0355380)

Gel Image

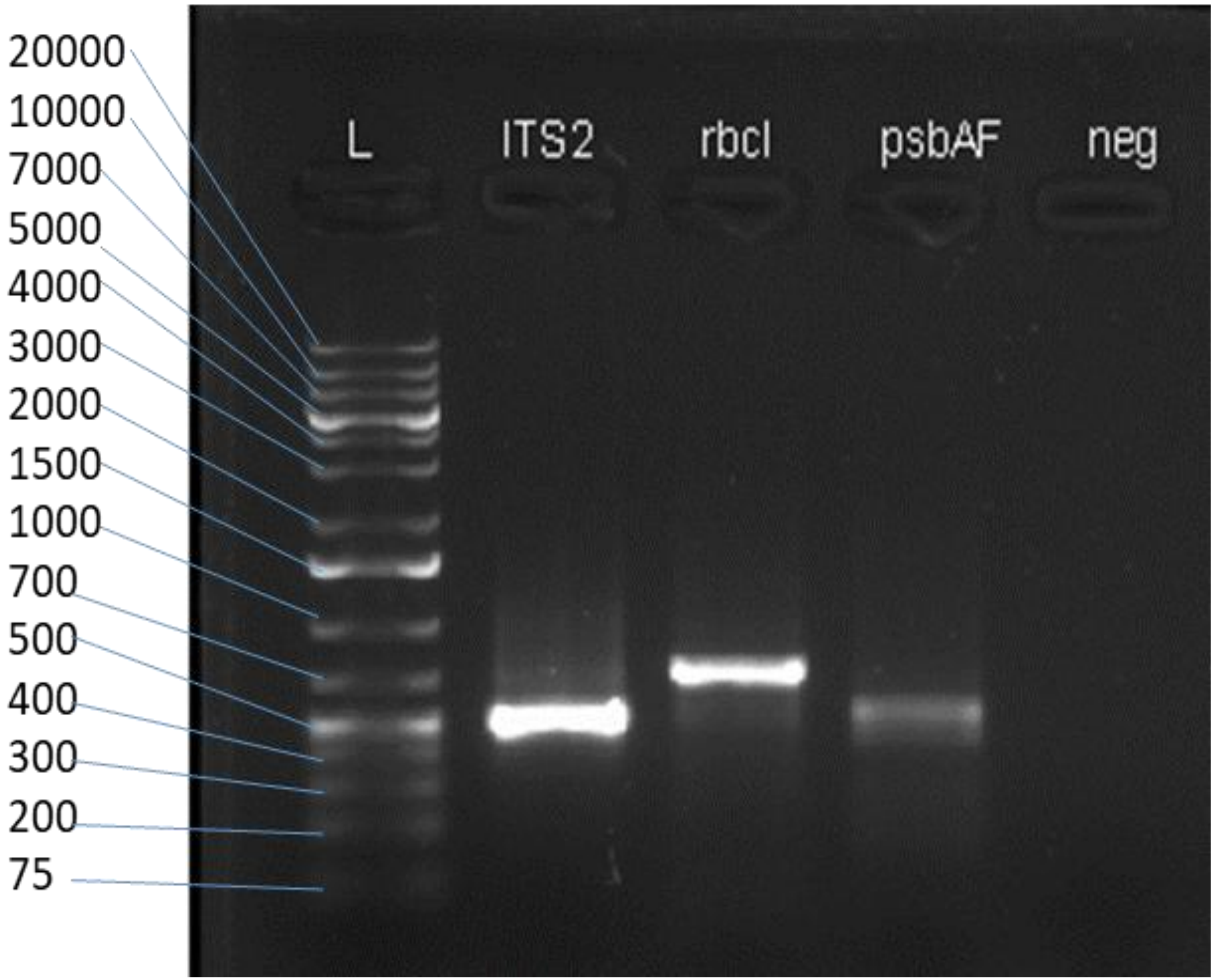

20000
10000
7000
5000
4000
3000
2000
1500
1000
700
500
400
300
200
75
L
ITS2
rbcl
psbAF
neg

Histology raw images

Group 1

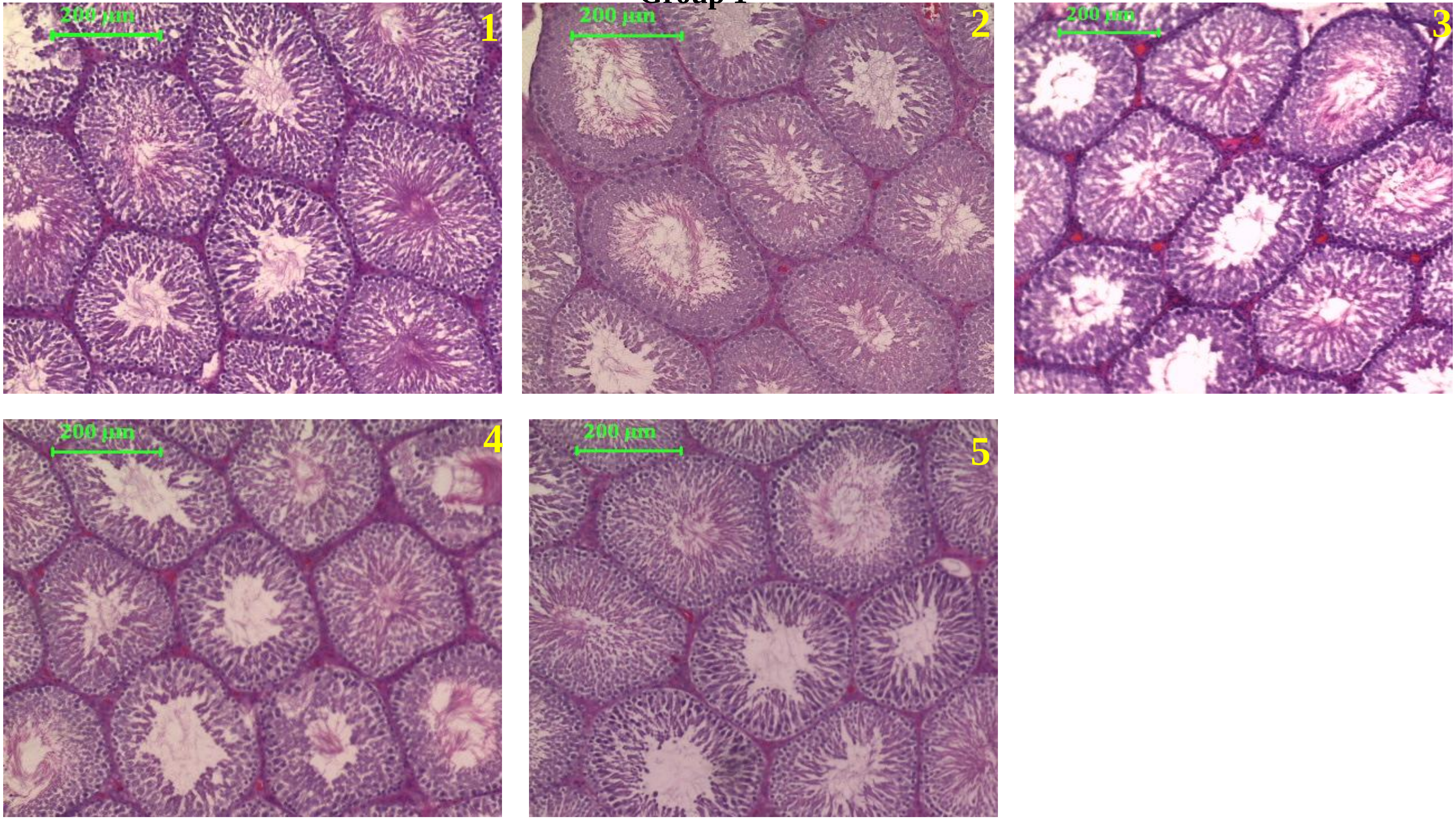

Group 1

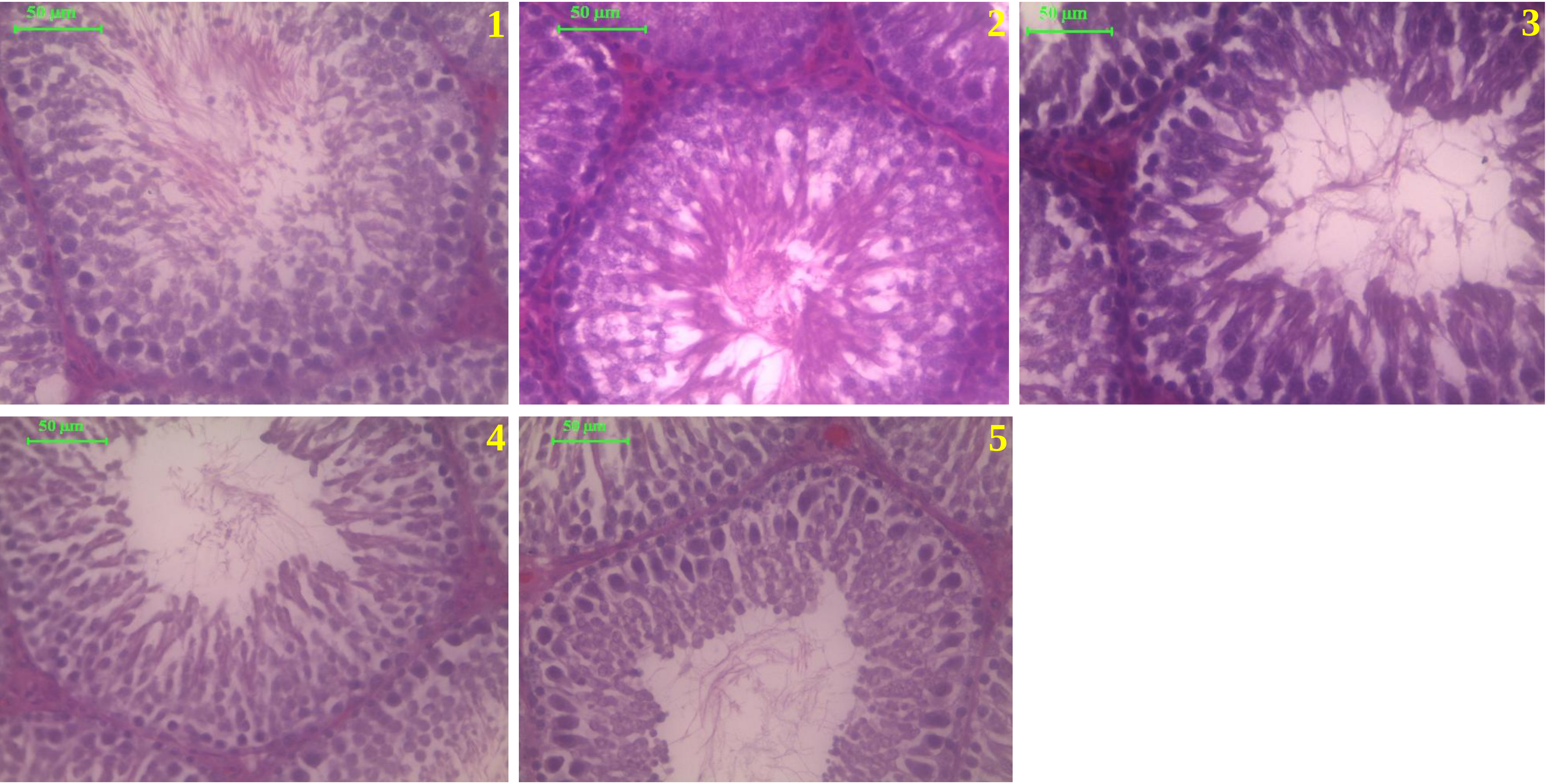

Group 2

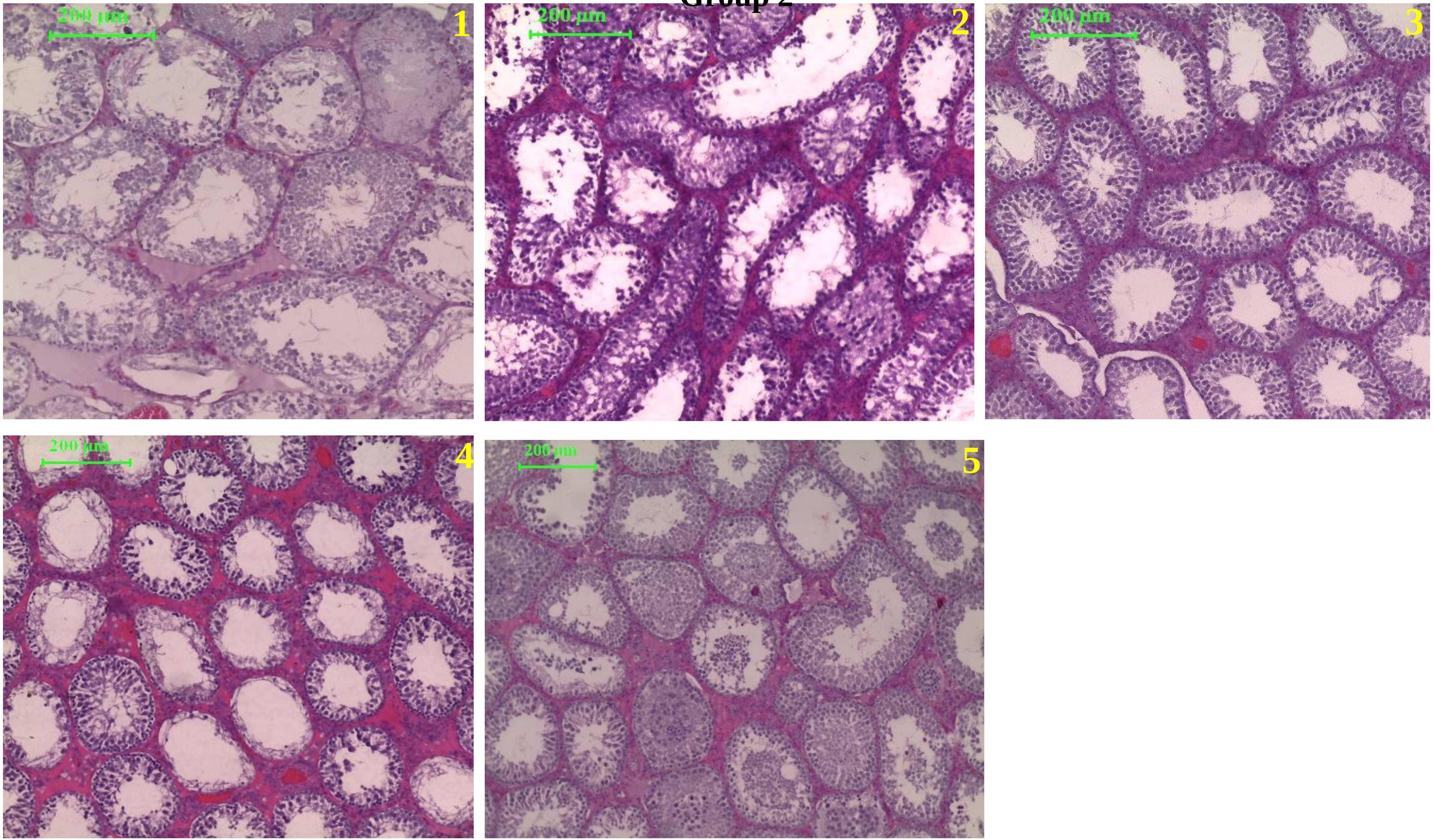

Group 2

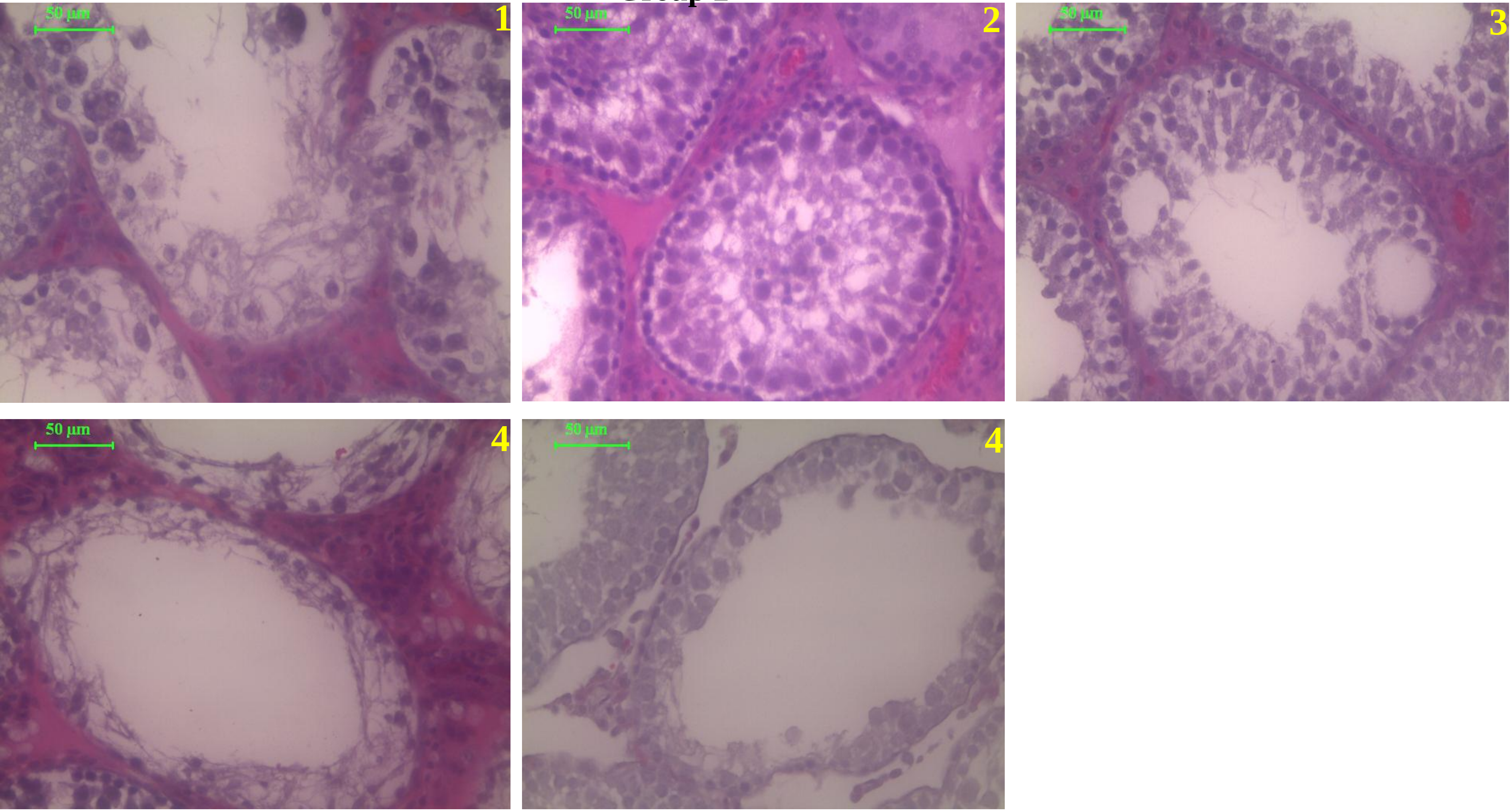

Group 3

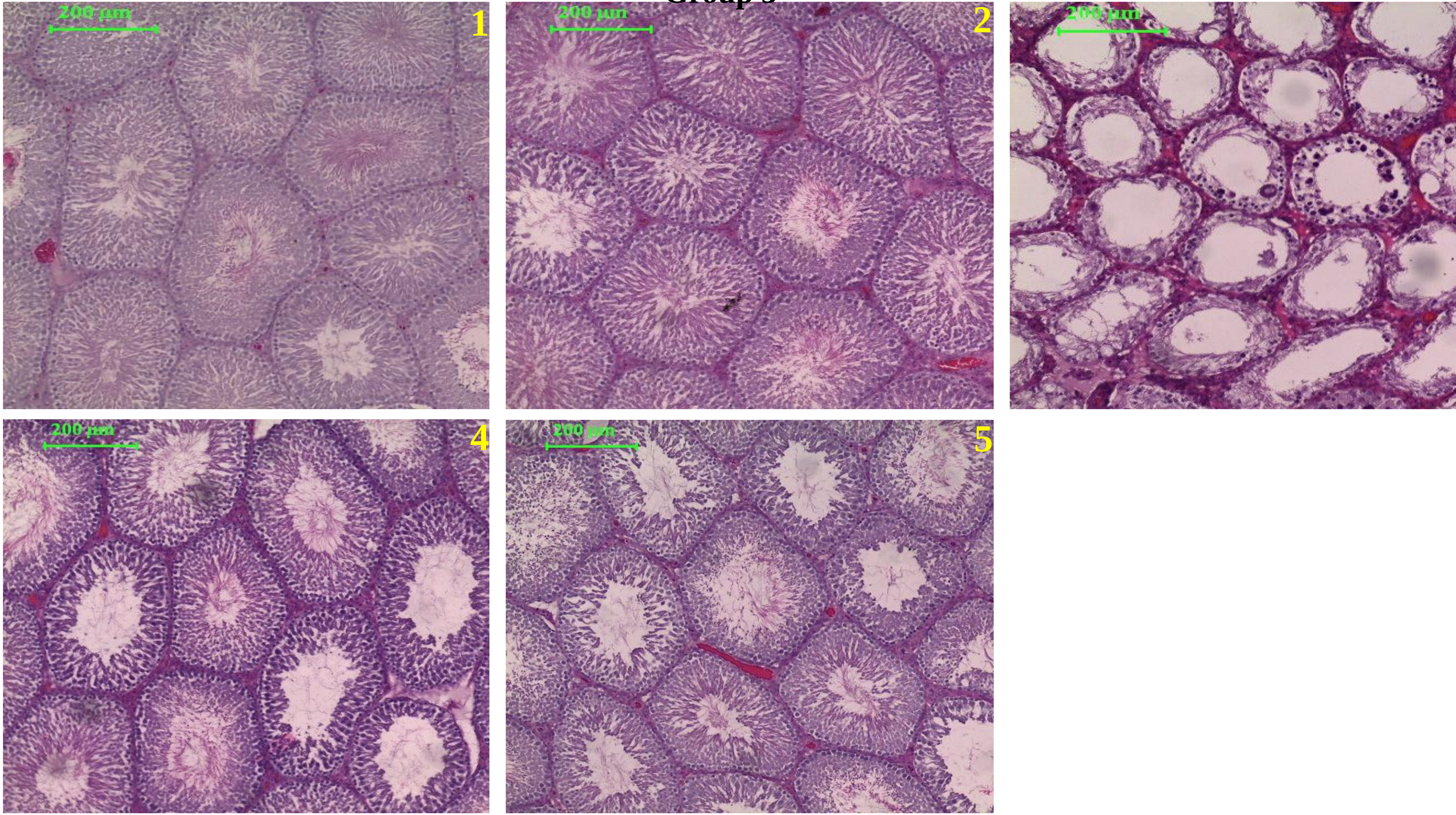

Group 3

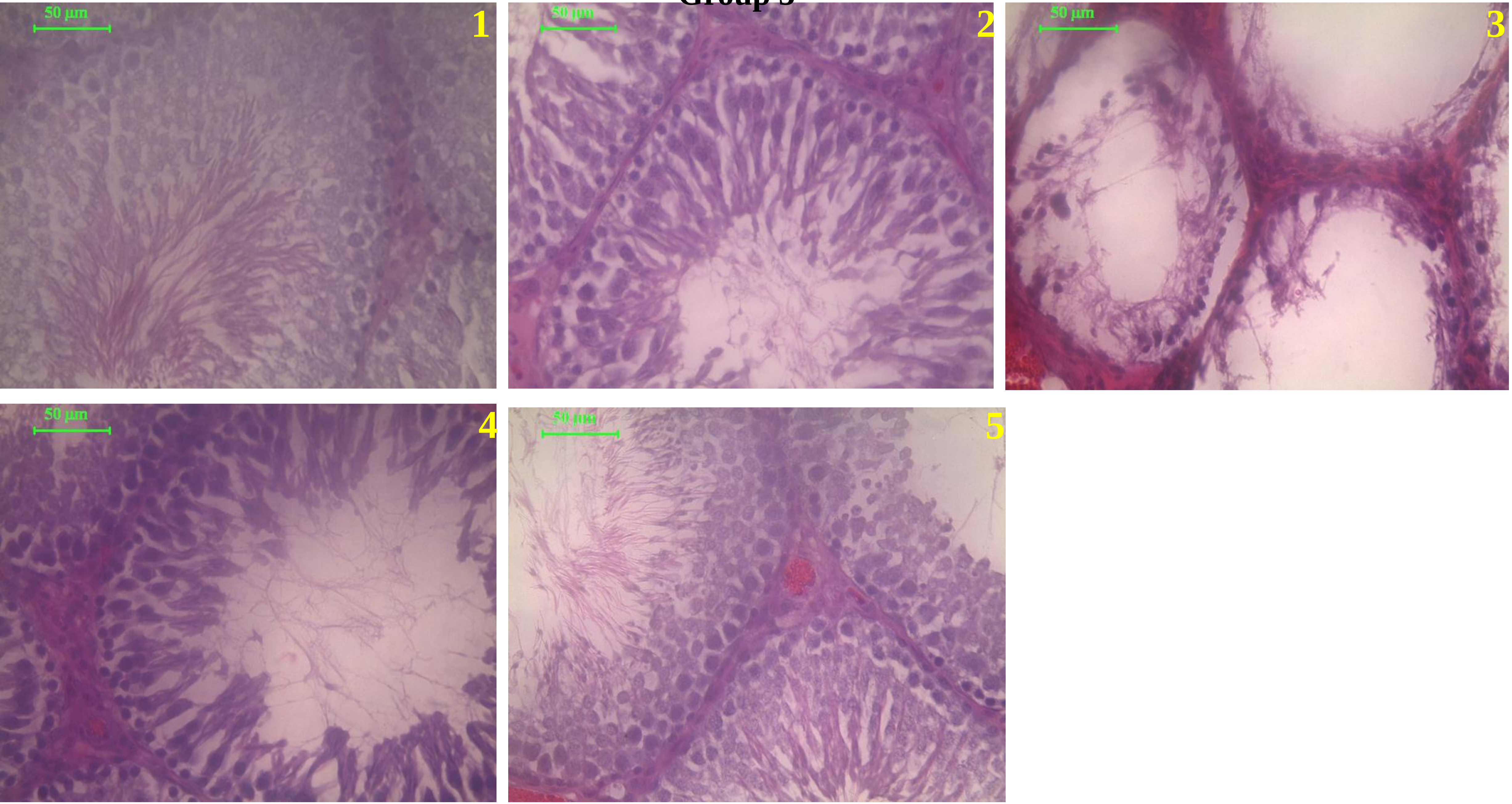

Group 4

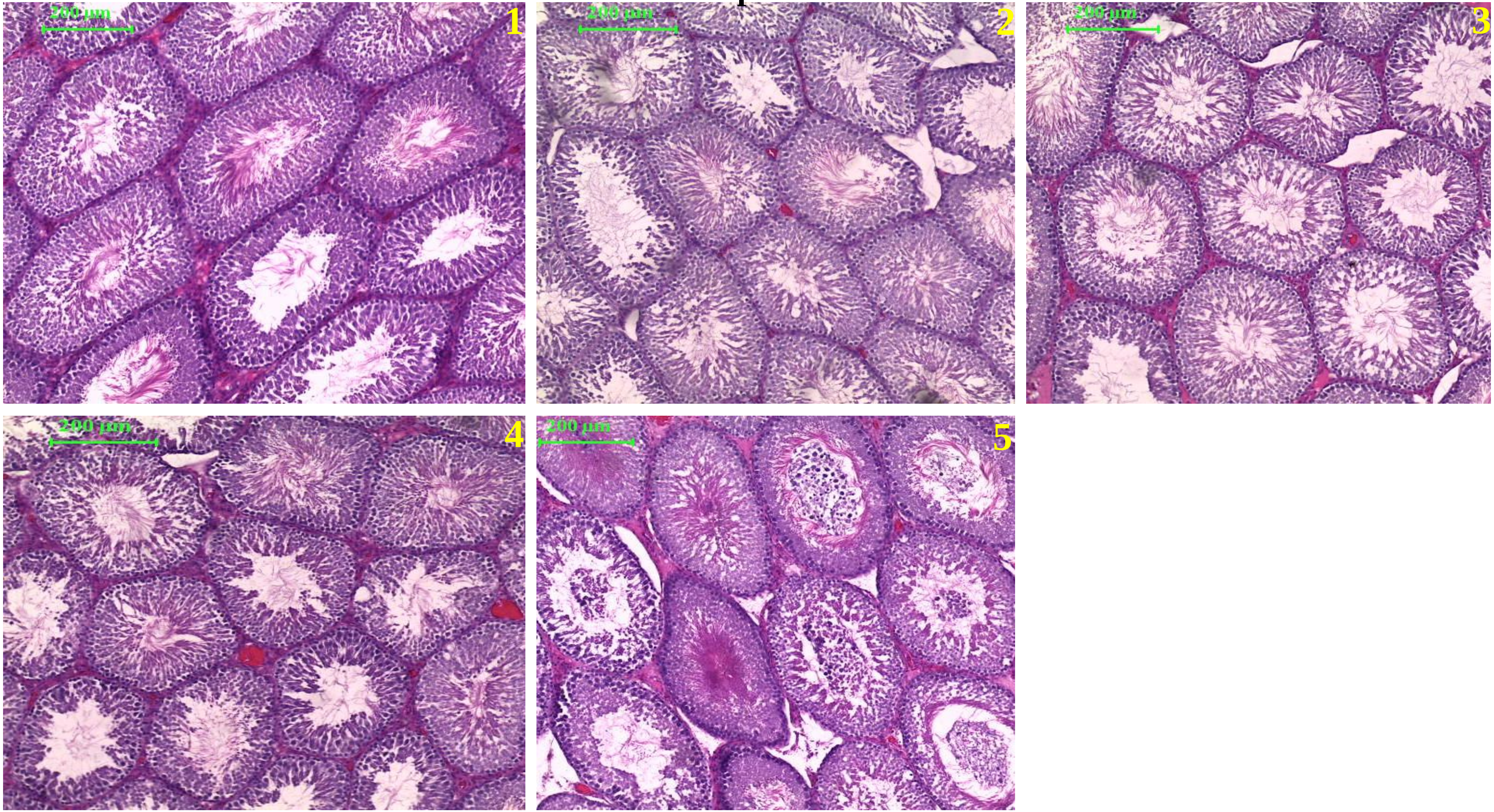

Group 4

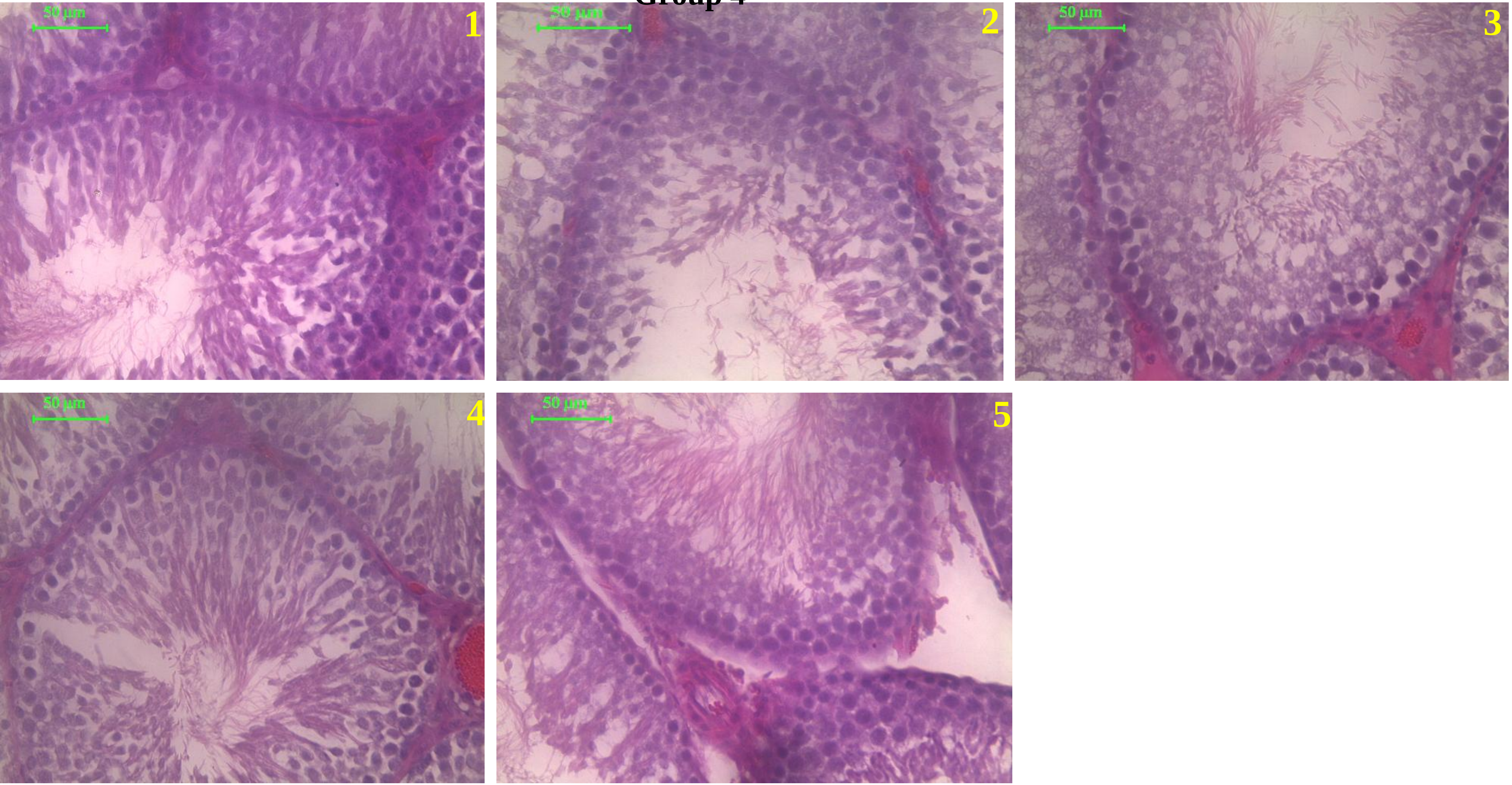

Group 5

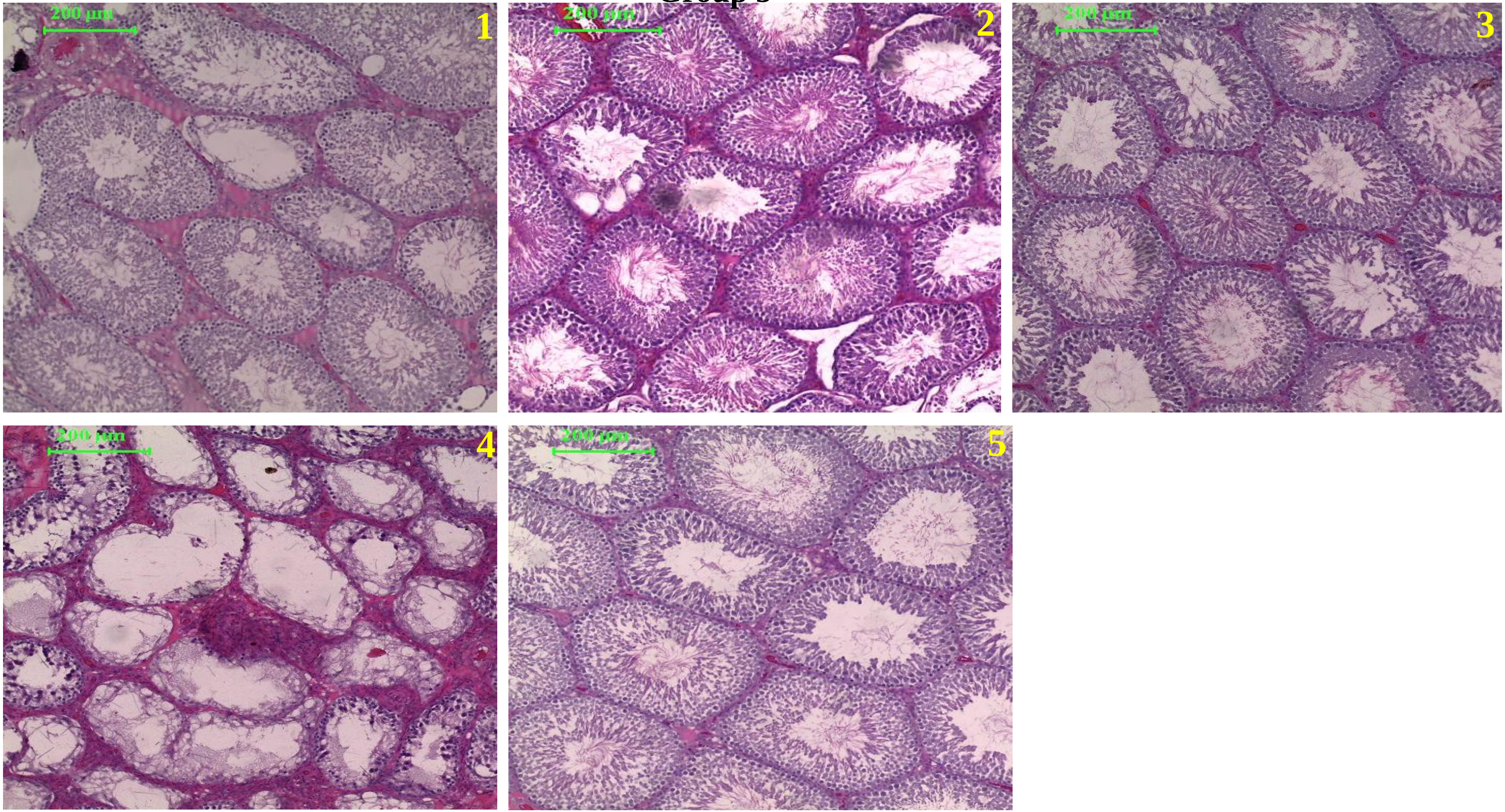

Group 5

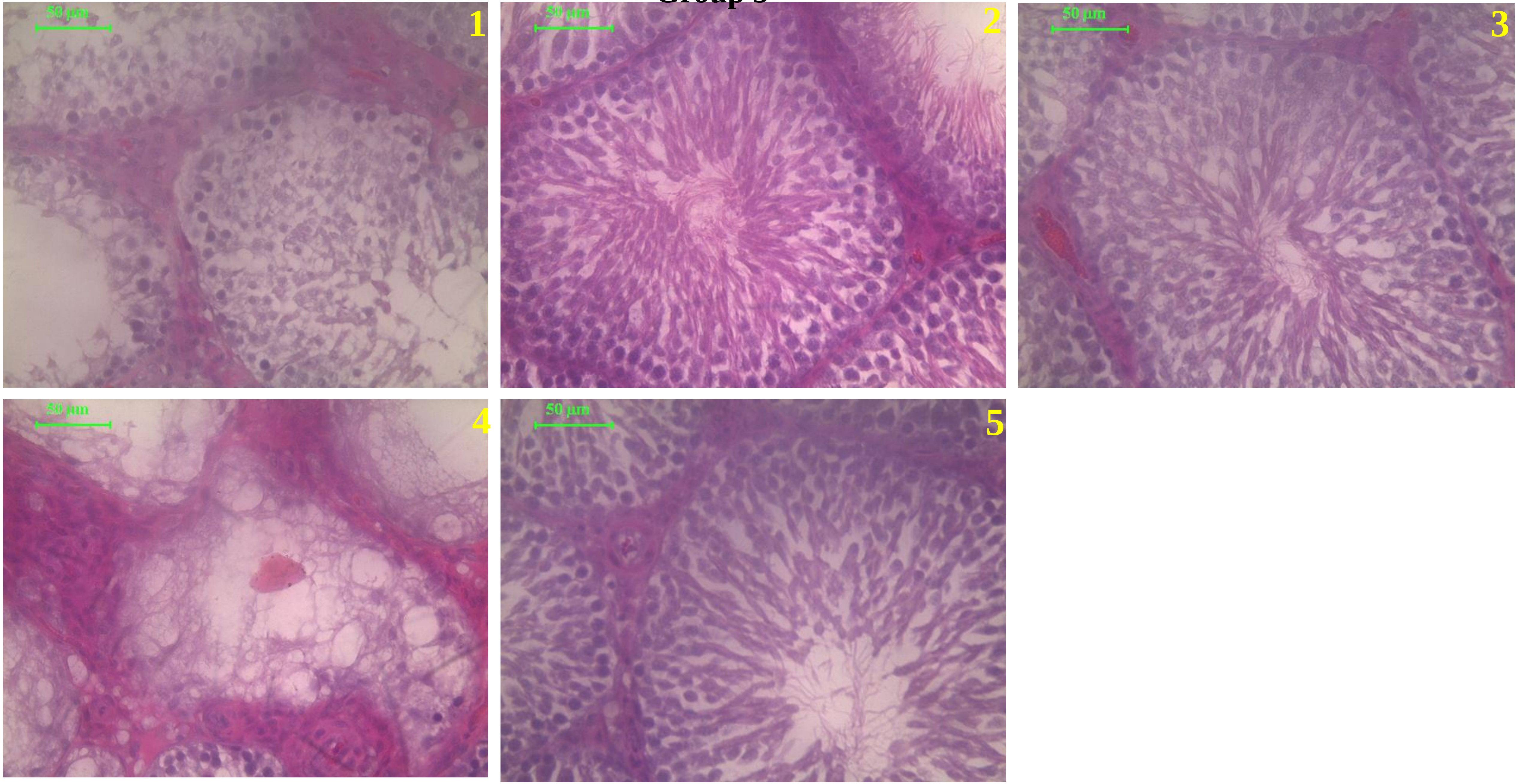

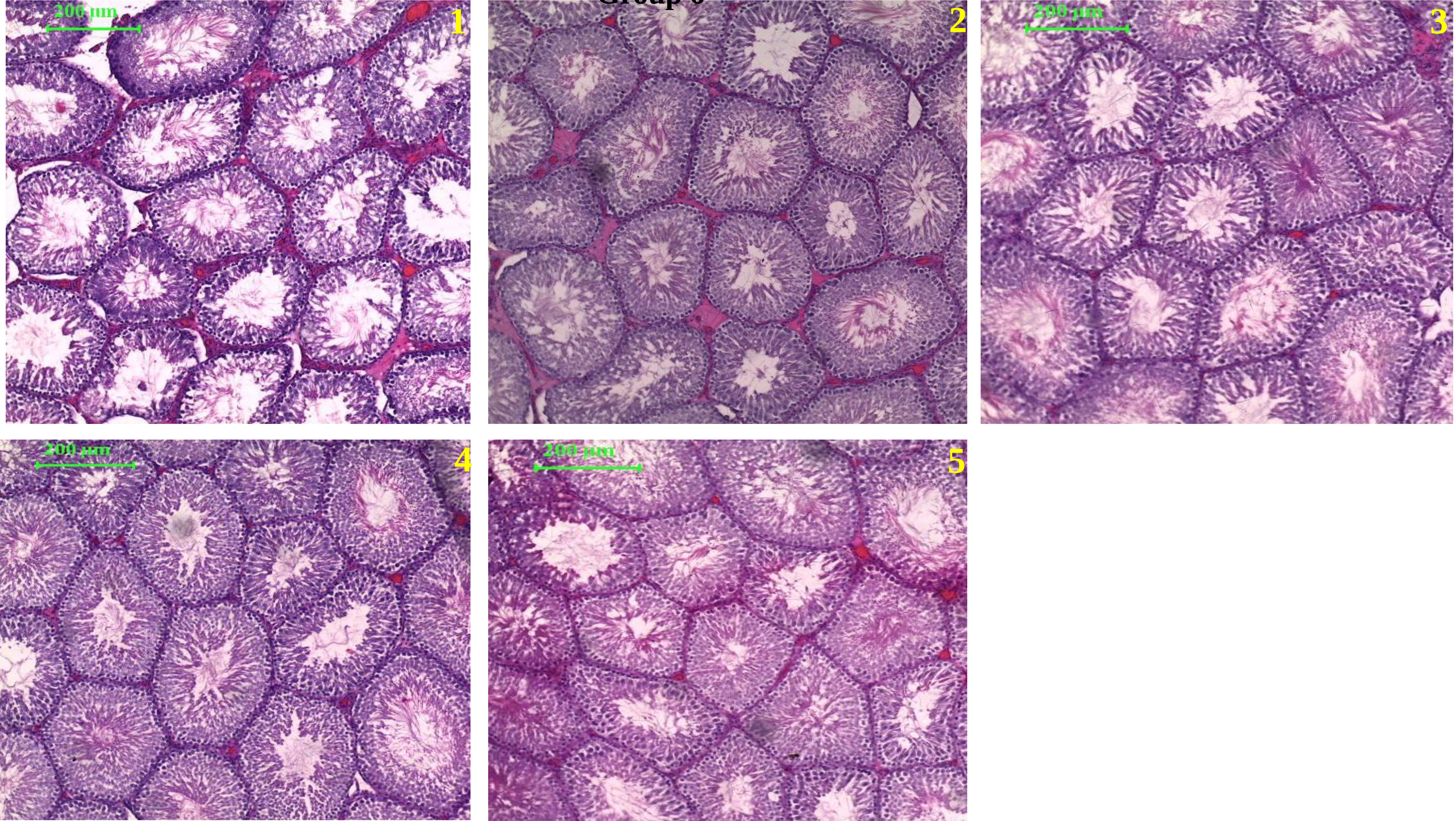
200 µm
1
2
200 µm
3
200 µm
4
200 µm
5

Group 6

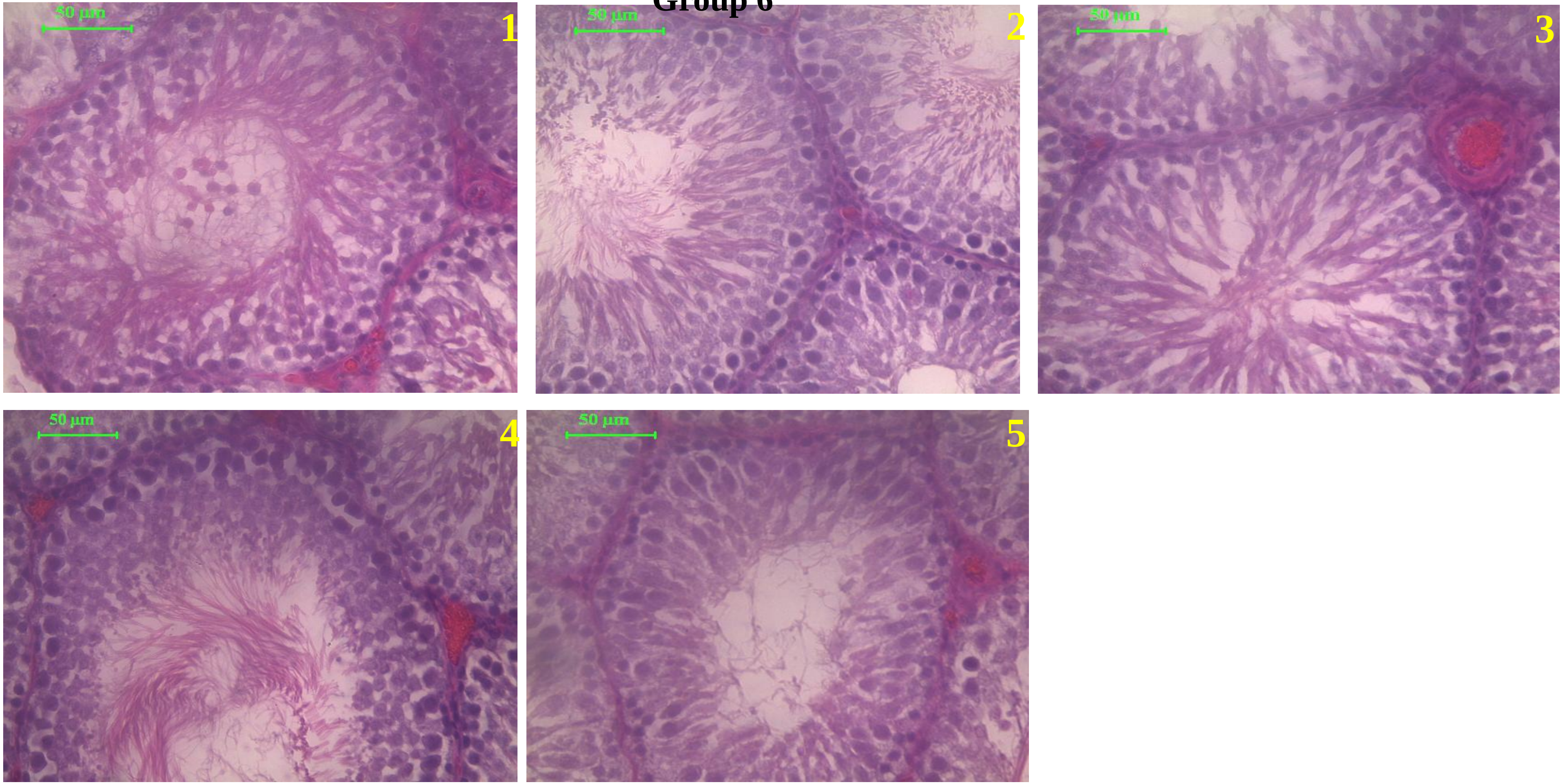

Supplement: Supplementary file 1 [file pone.0355380.s015.pdf]
