# Supplementary material for: Carissa spinarum L. aqueous root extracts ameliorate oxidative stress-induced infertility in adult male Wistar rats
Source: PLoS One. 2026 Aug 11;21(8):e0355380. doi: 10.1371/journal.pone.0355380 (PMC13460572; doi:10.1371/journal.pone.0355380)
Supplement: Supplementary file 2 [file pone.0355380.s017.pdf]

PLOS ONE

Carissa spinarum L. aqueous root extracts ameliorate oxidative stress-induced infertility in adult male wistar rats

--Manuscript Draft--

**Manuscript Number:**	PONE-D-25-63093
**Article Type:**	Research Article
**Full Title:**	Carissa spinarum L. aqueous root extracts ameliorate oxidative stress-induced infertility in adult male wistar rats
**Short Title:**	Carissa spinarum L. root extracts ameliorate oxidative stress-induced infertility in male wistar rats
**Corresponding Author:**	Martin Ouma, MSc.<br>University of Nairobi<br>Nairobi, KENYA
**Keywords:**	infertility, extracts, oxidative stress, phytochemicals, antioxidants, sperm cells
**Abstract:**	The potential of Carissa spinarum in treatment of male infertility arising from oxidative stress remains obscure, despite its traditional use in management of male infertility. This study evaluated the phytochemical profile and in vivo protective activities of C. spinarum extracts against oxidative stress-induced infertility in adult male Wistar rats. Root and leaf samples were extracted using solvents with different polarities and determined the phytochemicals and antioxidant activities. For in vivo studies, male Wistar rats were divided into six groups (n = 5). Group 1 (control), group 2 (metronidazole), group 3 (metronidazole and 250 mg/kg extract, group 4 (metronidazole and 500 mg/kg extract), group 5 (metronidazole and 1000 mg/kg extract) and group 6 (metronidazole + vitamin E + Sustanon). The epididymis was harvested for sperm characteristics and the testes for histology and biochemical assays. There were significantly higher ($p < 0.05$) phytochemicals and antioxidant activities in the leaves than the roots for all the solvents tested, except hexane. For in vivo studies, rats fed on extracts at 500 mg/kg body weight (BW) showed a significant ($p < 0.05$) increase in testicular weight index (0.98%), sperm count (37.28 × 10^6/mL), normal sperm morphology (60.50%), catalase (14.56 × 10-2 nmol g-1 testis tissue) and reduced lipid peroxidation (1.38 ± 0.14 nmoles/g tissue), malondialdehyde content. Extracts at low and high doses showed a significant increase in viable sperm cells (88.50%) and peroxidase, respectively. The extracts at 250 and 500 mg/kg BW ameliorated the effects of metronidazole on testicular histology. The study affirms the potential of C. spinarum extracts in the management of male infertility caused by oxidative stress-related factors.
**Order of Authors:**	Martin Ouma, MSc.
	Albert W. Nyongesa, Ph.D
	Gladys Bichang'a, PhD
	Edward Muge, PhD
	Evans Nyaboga, PhD
**Additional Information:**	
**Question**	**Response**
**Financial Disclosure**<br><br>Enter a financial disclosure statement that describes the sources of funding for the work included in this submission. Review the submission guidelines for detailed requirements. View published research articles from *PLOS ONE* for specific	The author(s) received no specific funding for this work.

examples.<br><br>This statement is required for submission and **will appear in the published article** if the submission is accepted. Please make sure it is accurate.<br><br>**Funded studies**<br>Enter a statement with the following details:<br>• Initials of the authors who received each award<br>• Grant numbers awarded to each author<br>• The full name of each funder<br>• URL of each funder website<br>• Did the sponsors or funders play any role in the study design, data collection and analysis, decision to publish, or preparation of the manuscript?<br><br>Did you receive funding for this work?	
**Competing Interests**<br><br>Use the instructions below to enter a competing interest statement for this submission. On behalf of all authors, disclose any competing interests that could be perceived to bias this work—acknowledging all financial support and any other relevant financial or non-financial competing interests.<br><br>This statement is required for submission and **will appear in the published article** if the submission is accepted. Please make sure it is accurate and that any funding sources listed in your Funding Information later in the submission form are also declared in your Financial Disclosure statement.<br><br>View published research articles from *PLOS ONE* for specific examples.	The authors have declared that no competing interests exist.

**NO authors have competing interests**<br><br>Enter: *The authors have declared that no competing interests exist.*<br><br>**Authors with competing interests**<br><br>Enter competing interest details beginning with this statement:<br><br>*I have read the journal's policy and the authors of this manuscript have the following competing interests: [insert competing interests here]*<br><br>* typeset	
**Ethics Statement**<br><br>Enter an ethics statement for this submission. This statement is required if the study involved:<br><br>• Human participants<br>• Human specimens or tissue<br>• Vertebrate animals or cephalopods<br>• Vertebrate embryos or tissues<br>• Field research<br><br>Write "N/A" if the submission does not require an ethics statement.<br><br>General guidance is provided below. Consult the submission guidelines for detailed instructions. **Make sure that all information entered here is included in the Methods section of the manuscript.**	Ethical approval was obtained from the Biosafety, Animal Use, and Ethics Committee of the Faculty of Veterinary Medicine, University of Nairobi (Reference number FVM BAUEC/2023/528).

**Format for specific study types**<br><br>**Human Subject Research (involving human participants and/or tissue)**<br>• Give the name of the institutional review board or ethics committee that approved the study<br>• Include the approval number and/or a statement indicating approval of this research<br>• Indicate the form of consent obtained (written/oral) or the reason that consent was not obtained (e.g. the data were analyzed anonymously)<br><br>**Animal Research (involving vertebrate animals, embryos or tissues)**<br>• Provide the name of the Institutional Animal Care and Use Committee (IACUC) or other relevant ethics board that reviewed the study protocol, and indicate whether they approved this research or granted a formal waiver of ethical approval<br>• Include an approval number if one was obtained<br>• If the study involved *non-human primates*, add *additional details* about animal welfare and steps taken to ameliorate suffering<br>• If anesthesia, euthanasia, or any kind of animal sacrifice is part of the study, include briefly which substances and/or methods were applied<br><br>**Field Research**<br><br>Include the following details if this study involves the collection of plant, animal, or other materials from a natural setting:<br>• Field permit number<br>• Name of the institution or relevant body that granted permission	
**Data Availability**<br><br>Authors are required to make all data underlying the findings described fully available, without restriction, and from the time of publication. PLOS allows rare exceptions to address legal and ethical concerns. See the PLOS Data Policy and FAQ for detailed information.	Yes - all data are fully available without restriction

A Data Availability Statement describing where the data can be found is required at submission. Your answers to this question constitute the Data Availability Statement and **will be published in the article**, if accepted.<br><br>**Important:** Stating 'data available on request from the author' is not sufficient. If your data are only available upon request, select 'No' for the first question and explain your exceptional situation in the text box.<br><br>Do the authors confirm that all data underlying the findings described in their manuscript are fully available without restriction?	
**Describe where the data may be found in full sentences. If you are copying our sample text, replace any instances of XXX with the appropriate details.**<br><br>• If the data are **held or will be held in a public repository**, include URLs, accession numbers or DOIs. If this information will only be available after acceptance, indicate this by ticking the box below. For example: *All XXX files are available from the XXX database (accession number(s) XXX, XXX.)*.<br>• If the data are all contained **within the manuscript and/or Supporting Information files**, enter the following: *All relevant data are within the manuscript and its Supporting Information files.*<br>• If neither of these applies but you are able to provide **details of access elsewhere**, with or without limitations, please do so. For example:<br><br>*Data cannot be shared publicly because of [XXX]. Data are available from the XXX Institutional Data Access / Ethics Committee (contact via XXX) for researchers who meet the criteria for access to confidential data.*<br><br>*The data underlying the results presented in the study are available from (include the name of the third party*	All relevant data are within the manuscript and its Supporting Information files.

and contact information or URL).<br>• This text is appropriate if the data are owned by a third party and authors do not have permission to share the data.<br><br>* typeset	
Additional data availability information:	

Manuscript
Click here to access/download;Manuscript;Manuscript Text_Ouma et al.docx

Carissa spinarum L. aqueous root extracts ameliorate oxidative stress-induced infertility in adult male wistar rats

Martin Ouma [a,*], Albert W. Nyongesa [b], Gladys B. Bichang'a [a], Edward K. Muge [a], and Evans N. Nyaboga [a]

[a] Department of Biochemistry, University of Nairobi, 30197 - 00100, Nairobi, Kenya

[b] Department of Veterinary Anatomy and Physiology, University of Nairobi, 19676 - 00202, Nairobi, Kenya

***Correspondence:**

Martin Ouma: martinioouma@gmail.com

Department of Biochemistry, University of Nairobi, P.O. Box 30197 - 00100, Nairobi, Kenya

// Abstract

The potential of *Carissa spinarum* in treatment of male infertility arising from oxidative stress remains obscure, despite its traditional use in management of male infertility. This study evaluated the phytochemical profile and *in vivo* protective activities of *C. spinarum* extracts against oxidative stress-induced infertility in adult male Wistar rats. Root and leaf samples were extracted using solvents with different polarities and determined the phytochemicals and antioxidant activities. For *in vivo* studies, male Wistar rats were divided into six groups (n = 5). Group 1 (control), group 2 (metronidazole), group 3 (metronidazole and 250 mg/kg extract, group 4 (metronidazole and 500 mg/kg extract), group 5 (metronidazole and 1000 mg/kg extract) and group 6 (metronidazole + vitamin E + Sustanon). The epididymis was harvested for sperm characteristics and the testes for histology and biochemical assays. There were significantly higher ($p < 0.05$) phytochemicals and antioxidant activities in the leaves than the roots for all the solvents tested, except hexane. For *in vivo* studies, rats fed on extracts at 500 mg/kg body weight (BW) showed a significant ($p < 0.05$) increase in testicular weight index (0.98%), sperm count (37.28 × 10^6/mL), normal sperm morphology (60.50%), catalase (14.56×10^{-2} nmol g^{-1} testis tissue) and reduced lipid peroxidation (1.38 ± 0.14 nmoles/g tissue), malondialdehyde content. Extracts at low and high doses showed a significant increase in viable sperm cells (88.50%) and peroxidase, respectively. The extracts at 250 and 500 mg/kg BW ameliorated the effects of metronidazole on testicular histology. The study affirms the potential of *C. spinarum* extracts in the management of male infertility caused by oxidative stress-related factors.

Key Words: infertility, extracts, oxidative stress, phytochemicals, antioxidants, sperm cells

Introduction

According to the World Health Organization (WHO), 48 million couples and 186 million persons globally are infertile. Infertility may arise as a result of male and female factors as well as environmental and genetic factors [1]. Male factors contribute to approximately 50% of all infertility cases, most of them being associated with abnormal sperm production and other reproductive disorders [2]. Exposure to toxic environmental agents such as industrial chemicals, agrochemicals, and drugs are leading contributors to the impairment of male reproductive physiological processes, including steroidogenesis and spermatogenesis [1]. Exposure to these agents been reported to impair male fertility by triggering pathways leading to the production of excess reactive oxygen species (ROS) and reactive nitrogen species (RNS). The generated free radicals reacts with various biomolecules including proteins, lipid membranes and DNA, resulting in protein oxidation, lipid peroxidation and DNA fragmentation that ultimately impairs spermatogenesis and related processes in the male gonads [2,3].

The use of medicinal plants in the treatment of various diseases and conditions including infertility has tremendously increased globally [4]. This is due to their natural abundance and minimal side effects on the body's functional systems compared to some chemically synthesized drugs [4]. Different medicinal plants are known for their rich phytochemical compounds that are capable of preventing oxidative stress-induced ailments, supporting their use by traditional herbalists in healing and as sources of active compounds used in drug design and development [5]. *Carissa spinarum* L. is one of the medicinal plants that grows in several regions of Kenya and has been reported to have male fertility promoting properties in traditional medicine [6,7]. Phytochemical evaluation of *C. spinarum* revealed the presence of different classes of therapeutic secondary metabolites including phenols, flavonoids, tannins, alkaloids, steroids, and

fatty acid esters as well as characterized compounds such as (+)-isolariciresinol 3a-*O*-*β*-D-glucopyranoside, erythro-1-(3-methoxy-4-hydroxy-phenyl)-propan-1,2-diol, threo-1-(3-methoxy-4-hydroxy-phenyl)-propan-1,2-diol and (6*R*,7*S*,8*S*)-7a-[(*β*-D-glucopyranosyl)-oxy]-1-methoxyisolariciresinol from the root back of *C. spinarum*[8,9].

Despite *C. spinarum* having potential traditional medicinal applications in the treatment of oxidative stress-related conditions, including infertility, the scientific support in revealing the mechanism of its action on infertility cases as claimed by herbalists and ethnopharmacological surveys remains obscure and have not been explored. Its potential protective and curative effects towards infertility arising from testicular oxidative damage are equally unclear. This study aimed to evaluate phytochemical profiles of *C. spinarum* and its testicular protective potential of aqueous root extracts of *C. spinarum* against oxidative stress-induced infertility in adult male Wistar rats, with induction being achieved by oral administration of 400 mg/kg body weight of metronidazole as reported by [10,11]. We hypothesized that the aqueous extracts of *C. spinarum* roots possess phytochemical compounds with antioxidant potential that suppress oxidative damage on testicular tissue cells that arise from oxidative stress and which can impair spermatogenesis and steroidogenesis. The study used male Wistar rats since rats have been widely used as models for fertility studies [12].

Materials and methods

Plant material collection, molecular identification and sample extraction process

Carissa spinarum L. leaf and root samples were collected from Eor-Ewuaso village, Narok County, Kenya, (coordinates -0.9690953596611817, 35.6458956346891). The samples were

packed in airtight containers and transported to the University of Nairobi within 12 h of collection. Identification was made by a plant taxonomist and samples deposited in the herbarium at the Department of Biology, University of Nairobi (code of voucher specimen MOUNBI 2023/001). The leaf samples were stored at -80 °C for molecular authentication of the plants identity. The leaves and root backs of (woody roots) were peeled off and dried for two weeks then ground into fine powder and stored at 4 °C.

Molecular identification of the plant sample was carried out based on DNA barcoding, as described by Pere *et al*. [13]. Genomic DNA extraction was performed using Qiagen DNeasy® Plant Maxi Kit (Catalogue No. 68163, Qiagen, Germany) was used to extract genomic DNA from *C. spinarum L.* leaves according to the manufacturer's instructions. This was followed by PCR amplification targeting *trnH-PsbA* and *rbcl* regions, with the resulting sequences subjected to sequencing using Sanger sequencing chemistry (Macrogen, Netherlands)

The resulting forward and reverse sequences were used in constructing contig sequences on Bioedit software version 5.0.9 [14] followed by trimming low-quality end sequences manually and correction of miscellaneous bases based on the chromatogram peaks. The cleaned sequences were BLASTed on GenBank database (https://blast.ncbi.nlm.nih.gov/Blast.cgi (accessed on 4th May 2024), suitable sequences were selected based on the following features; at least 80% identity, ≥ 95% query coverage, and zero expected (E) value. The sequences were then used for phylogenetic analysis using the Bayesian phylogenetic approach of MrBayes, version 3.1.2 [15]. A total of 12,000,000 phylogenetic trees were created, and from every 1000 generations, a tree was sampled. The 'sum p' and 'sum t' in MrBayer, were used in constructing the consensus tree

with the branching confidence being calculated as the posterior probabilities and later visualized in Figtree, version 1.4.3 (http://tree.bio.ed.ac.uk/)

Preparation of crude extracts

To understand solvent efficacy, extraction for phytochemical analysis was carried out in varying polarity solvent systems, which included: ethanol, methanol, hydromethanol (70%), hydroethanol (70%), n-hexane, acetone, cold and hot distilled water. Extraction was done using the cold maceration method as described by Altemimi *et al* [16]. Additionally, hot maceration with distilled water (for *in vivo* studies) was also incorporated in the extraction as reported by Njau *et al*. [17]. Spectrophotometric assays were done using UV-Vis spectrophotometer (UVmini-1240, Shimadzu, Duisburg, Germany).

Quantitative phytochemical analysis

Phenolic content was determined in all extracts following the Folin-Ciocalteu (FC) method described by Siddiqui *et al*. [18], Briefly, 200 µL of crude extracts were transferred into clean test tubes and 1.8 mL of distilled water added. Thereafter, 200 µL of 10% Folin–Ciocalteu reagent was added to each tube and mixed by shaking before addition of 7.5% sodium carbonate, mixed and incubated in the dark at room temperature for 60 min. The absorbance was then read at 725 nm against the blank (containing all reagents except the extract). The Gallic acid standard was prepared from 1 mg/mL stock with concentrations 0, 20, 40, 60, 80, 100, 120, 140, 160, 180 and 200 µg/mL, respectively. The concentration of phenols was expressed as µg Gallic acid equivalent/gram of dry plant material (GAE/g).

Total flavonoid content was determined based on aluminium chloride reaction as described by Aryal *et al* [19]. Briefly, 0.25 mL of crude extracts were transferred into clean test tubes, followed by the addition of 1.5 mL of absolute Methanol. A volume of 0.1 mL of 10%

Aluminium chloride solution was added to the mixture and mixed by shaking, followed by the addition of 0.1 mL of 1M sodium Acetate and 2.5 mL of distilled water, mixed by shaking, and then incubated at room temperature for 30 min. The absorbance was read at 415 nm using blank (with all other reagents except the extract). Rutin standard was prepared from a 10 mg/mL stock with concentrations 0.0, 0.5, 1.0, 1.5, 2.0, 2.5, 3, 3.5, and 4.0 mg/mL. Concentrations of flavonoids were expressed in mg Rutin equivalent/gram of dry plant material (RE/g).

The total tannin content in each extract was measured based on Folin-Ciocalteu as described by Makkar [20]. The concentrations of tannins were determined by first measuring the Total phenolic content using the Folin-Ciocalteu method [18], and expressed as Tannic Acid equivalents. Tannins were then removed from the tannin-containing extracts by mixing 1 mL of it with 1 mL of 1% (w/v) insoluble polyvinyl polypyrrolidone. The mixture was then kept at 4℃ for 15 min, vortexed, and centrifuged at 3000 ×g for 10 min. The supernatant containing simple phenolics was transferred into a clean Eppendorf tube and used for assaying TPC and expressed in Tannic Acid equivalents. The pellets containing PVPP-bound tannins were discarded and the difference in concentration due to reduction in absorbance at 725 nm for the two assays was considered as TTC. The tannic acid standard was prepared from a 1mg/mL stock and used for the determination of the concentrations.

Alkaloid concentration was determined as described by Ajanal *et al*. [21]. Briefly, samples previously extracted in different solvents were obtained, and 1 mL of each extract was mixed with 1 mL of 2 N HCl to dissolve the alkaloids present in the samples. The mixture was shaken vigorously and centrifuged at 1000 xg for 5 *min* to sediment the unwanted debris. The

supernatant (1 mL) of each was transferred into the separating funnel and washed thrice with 10 mL of chloroform before the addition of 0.2 N sodium hydroxide dropwise until a neutral pH was attained. Volumes of 5 mL of each 0.1 M phosphate buffer (pH 4.7) and BCG were then added to the mixture and shaken to allow the formation of the yellow complex. The mixture was then extracted with 1, 2, 3, and 4 mL of chloroform by shaking vigorously and collected in a 10 mL volumetric flask.

The standard was prepared in the same manner, except the extract was replaced by different concentrations of Atropine, from a 1mg/mL stock.

Evaluation of in vitro antioxidant activity

Ferric Reducing Antioxidant Power (FRAP) was assessed as described by Wang *et al*. [22], with ascorbic acid (200 mg) used as the standard, where 1mL of each extract was added to clean test tubes, mixed with 2 mL of 0.1M Phosphate Buffered Saline (PBS) (pH 6.6) and 2 mL of potassium ferricyanide (K3[Fe(CN)6]) (1% w/v). The mixture was placed in a water bath maintained at 50 ℃ for 30 min. The samples were removed and allowed to cool, then 2 mL of 10% Tetrachloroacetic acid (TCA) was added, and the mixture was centrifuged at 1000 xg at room temperature for 10 min. The supernatant (2 mL) was transferred and mixed with equal volumes of ultrapure water and 0.5 mL of Ferric chloride solution (FeCl3) (0.1%, w/v). The mixture was allowed to stand for 10 min, after which the absorbance was read at 700 nm.

Hydrogen peroxide radical scavenging activity was performed according to the method described Ruch *et al*. [23], with ascorbic acid (200 mg) used as the standard. Briefly, 0.1 mL of crude extracts (leaves and roots) was separately added into clean test-tubes. This was followed by the addition of 0.3 mL of 1X PBS pH 7.4 and mixed by shaking. A volume of 0.6

mL of 40 mM H2O2 was added to the mixture and allowed to mix by shaking. After 10 min of reaction time, the absorbance of the samples, control, and Standard (Vitamin C) was read at 230 nm.

Hydrogen peroxide radical scavenging was calculated according to equation (1):

$$H_2O_2\ Scavenging\ activity\ (\%) = \frac{Ao-A1}{Ao} \times 100 \ldots\ldots\ldots\ldots\ldots\ldots\ldots \text{Equation i}$$

Where: Ao is the absorbance of control (containing Buffer, water and Hydrogen peroxide), A1 is the absorbance of samples/standard antioxidant (Vitamin C).

Compound identification by GC-MS analysis

Root and leaf powdered samples, macerated with ethanol and acetone (the most effective solvents of extraction according to principal component analysis), were analyzed for volatile organic compounds using a GC-MS system (GCMS-QP2010 SE, Shimadzu, Kyoto, Japan). The program parameters were set as follows before running: the column oven temperature was set to 70℃, the injection temperature to 250℃, the injection mode to split, and the sampling time to 1 min. The carrier gas was helium (99.999%) at 8.0 psi, with a total flow rate of 13.2 mL/min, a column flow rate of 0.92 mL/min, and a split ratio of 10.1. The extracts were separated at programmed GC oven temperatures as follows: 70℃ for 5 sec, 10℃/min up to 250℃ for 8 min, and 5℃/min up to 270℃ for 5 min. The MS adjustments included an ion source temperature of 200℃, an interface temperature of 250℃, and a solvent cut time of 3 min. The total program time dedicated to the separation was 40 min. The separated components were translated into mass spectral peaks and identified by comparison with the built-in National Institute of Standards and Technology (NIST) GC-MS mass spectral library.

Mineral content analysis

Root and leaf samples were first prepared following the standard operating procedure at the Kenya Bureau of Standards (KEBS, TES/ING/TM/05). Briefly, the preparation was initiated by ashing 1 g of each overnight in a muffle furnace. The products were separately mixed with 10 mL of concentrated nitric acid and heated on a hot plate until a clear solution was obtained. The solution was diluted to a total volume of 50 mL using deionized water; this was performed in triplicate. Analysis of each element was performed based on its characteristic maximum absorption wavelength for macro-minerals (Ca, Na, K, Mg, P, S), trace elements (Se, Fe, Zn, Mn, Mo, Cu, Cr, Si), and non-essential elements (Ba and B) were performed using the ICP-OES instrument (5100S, Agilent) following the protocol described by Ngigi and Muraguri [24], and the concentration recorded in parts per million (ppm).

In vivo animal model studies

Dose limit test

To assess the safety of the extract doses for use in animal models, a dose-limiting test was conducted, since the root extracts of *C. spinarum* have been used as herbal remedies for other diseases without causing toxicity in humans. This test was performed according to OECD test guidelines 423 (limit test) [25], to determine the safety of the experimental doses. Six non-pregnant, nulliparous female Wister rats weighing 158.67 ± 1.49 g, aged 63-70 days, were randomLy selected and marked for the study. The animals were fed standard rat pellets and kept at room temperature (22 ± 1.3 ℃) with a 12-hour light/dark cycle. Before administering the doses, the animals fasted for 5 hours but had free access to water. A single dose of 2000 mg/kg body weight (bw) of *C. spinarum* aqueous root extract was given to the first animal. After its survival, two additional animals received the same dose and were monitored for 30 *min*, 1 hour, and 4 hours. During this period, the animals were not fed for 3 hours. Once no animal was

confirmed dead, three more animals were given 1 mL of distilled water as the control group. The animals in both treatment and control groups were then observed for signs of toxicity over the next 6 hours and daily for 14 days, with body weights recorded regularly. At the end of the study (day 15), the animals were sacrificed by cervical dislocation.

Animal handling and experimental design

All animals used in this study were handled according to ethical regulations for laboratory animal use. Adult male Wistar rats (aged 70–90 days, weighing 130–180 g) were obtained from the Department of Biology, University of Nairobi. They were housed in a clean environment with a 12-hour light/dark cycle, relative humidity of 56–65%, and at room temperature (23 ± 1°C). The animals had free access to water and were fed standard rat pellets. They were acclimatized to the animal house for one week before the start of the study, during which they underwent 5 *min* of daily handling to familiarize them with human contact. A qualified veterinarian regularly monitored their health, and only healthy animals were used for the research. The study complied with internationally accepted principles for laboratory animal care, as outlined in the European Community guidelines (EEC Directive of 1986; 86/609/EEC). Ethical approval was obtained from the Biosafety, Animal Use, and Ethics Committee of the Faculty of Veterinary Medicine (Reference number FVM BAUEC/2023/528).

Thirty (30) rats were randomLy divided into six experimental groups. They received treatments with different extract doses confirmed to be safe based on the dose limit test, following OECD 423 guidelines, with no animals dying or showing signs of toxicity after a single 2000 mg/kg body weight dose. Group 1 (Normal control) received 1 mL/200 g body weight of distilled water, Group 2 (Negative control) received 400 mg/kg metronidazole alone, Group 3 (Low extract dose

group) received co-administration of 400 mg/kg body weight of metronidazole and 250 mg/kg body weight of *C. spinarum* aqueous root extract, Group 4 (Medium extract dose group) received co-administration of 400 mg/kg body weight of metronidazole and 500 mg/kg body weight of *C. spinarum* aqueous root extract, Group 5 (High extract dose group) received co-administration of 400 mg/kg body weight of metronidazole and 1000 mg/kg body weight of *C. spinarum* aqueous root extract, while Group 6 (Positive control) received 400 mg/kg body weight of metronidazole, 400 mg/kg body weight of vitamin E, and 0.36 mg/kg body weight of Sustanon. Sustanon (testosterone agonist) for the positive control treatment was administered once a week via intramuscular injection; all other treatments were given orally daily for 56 days.

Sample collection and processing

At the end of the treatment period, the animals were deeply anaesthetized through intraperitoneal injection of 45 mg/kg sodium pentobarbital. The testis was dissected, fat trimmed off, washed in 1× phosphate-buffered saline (PBS) (pH 7.4), separated from the epididymis, and then weighed. The left testis was immediately fixed in 10% phosphate-buffered formaldehyde (pH 7.4) for histological examination, while the right testis was stored at -20 ℃ for biochemical assays [10]. The left and right cauda epididymis were separated from the rest of the epididymis, weighed, and dissected for sperm retrieval and analysis.

Sperm were collected according to the procedure described by Al-Alami *et al*. [10]. Briefly, the left and right cauda epididymis were placed in Petri dishes containing 10 mL of PBS buffer (pH 7.4) maintained at 37 ℃ and minced using a scalpel blade. The spermatozoa were allowed to diffuse into the buffer for 2 min and swirled to obtain a homogeneous suspension for sperm characteristic analysis. The sperm cell suspension was maintained in a pre-warmed water bath at 37 °C throughout the analysis.

Evaluation of sperm characteristics

Sperm count was done according to the method described by Al-Alami *et al*. [10] and *WHO Laboratory Manual for the Examination and Processing of Human Semen* [26]. Briefly, an aliquot of 10 µL of the homogenous sperm suspension was loaded on each of the chambers of the haemocytometer covered with a coverslip and allowed to settle for 1 min before counting the sperm cells in the five large squares (four at the corners and the middle one) at ×100 total magnification under a light microscope (Olympus Microscope BH-2, USA). Sperm cells with their heads completely in the 5 medium squares or those lying on the top or left side of the square but on the line were counted (excluding those lying on the right or bottom sides of the middle large square). The counts were performed for the two counting chambers and averaged. The concentration of the spermatozoa in 1 mL of the suspension was calculated according to equation (ii).

$$Concentration\ (\frac{Count}{mL}) = (Dilution\ factor) \times (Count\ in\ 5\ squares) \times (0.05 \times 10^6) \ldots\ldots\ldots Equation\ ii$$

Sperm viability was assessed based on staining of the sperm cells with eosin-nigrosine stain, according to the *WHO Laboratory Manual for the Examination and Processing of Human Semen* [26], whereby, 50 µL of properly mixed sperm suspension was added to equal volumes of eosin Y-nigrosin stain (prepared according to the WHO manual), mixed, and allowed to stand for 30 seconds on a microscope slide. A cover slip was immediately placed, and 200 spermatozoa were evaluated for each sample at 1000X magnification under oil immersion. Deep pink, red, and dark-stained heads (dead) and unstained/bright heads (live) were counted and recorded.

Sperm morphology was assessed according to the protocol described by Kondracki *et al*. [27], where thin smears of the spermatozoa were made on a microscope slide, stained with 1% eosin-Y, and counterstained with 5% nigrosin. Spermatozoa (200 for each sample) were examined at 400X light microscope magnification. Head and tail defects were recorded and expressed as a percentage of the total count.

Testicular tissue processing for histology

Testicular tissues were prepared for histological examination following the protocol described by [28]. The formaldehyde-fixed testes samples were washed overnight in running water, followed by dehydration in increasing ethanol concentrations (50%, 70%, 80%, 90%, 95%, and 100%) at an interval of 1 h. The tissues were then cleared using methyl benzoate for 1 h, followed by infiltration using molten paraffin wax at 65 °C for 30 min, and finally embedded in paraffin wax to solidify overnight. The wax-containing samples were then placed on a wooden block for blocking after which tissue sections of 5 µM thick were cut using Leitz Weitzlar® rotary microtome (Leica Microsystems, Germany). The sections were mounted on a glass slide, deparaffinized in xylene and rehydrated through treatment in a decreasing ethanol concentration (100%, 90%, 70%, 50%) and finally in water. The rehydrated sections were then stained using Haematoxylin and counterstained with eosin, dried and examined at 1000× total magnification using Leica® DM 500 (Leica Microsystems, Germany) light microscope.

Malondialdehyde levels as an oxidative stress marker in the testis

Lipid peroxidation was assessed by measuring malondialdehyde levels in the testis according to the protocol described by Chen & Gallie [29]. Briefly, 0.05 g of testis was weighed and homogenized in 1 mL of ice-cold 0.1% (w/v) Trichloroacetic acid (TCA) using a mortar and pestle. The mixture was transferred to an Eppendorf tube, and 0.25 mL of 0.1% (w/v) TCA was added, then centrifuged at 000 xg for 5 *min* at 4°C. Next, 0.25 mL of the supernatant was transferred into clean Eppendorf tubes and mixed with 0.5 mL of 20% (w/v) TCA containing 0.5% (w/v) Thiobarbituric Acid (TBA). The mixture was incubated in a water bath at 95 °C for 30 *min*, cooled on ice, and then centrifuged again at 4,000 xg for 10 *min* at 4°C. Absorbance was measured at 532 nm and 600 nm using a UV-visible spectrophotometer (UVmini-1240, Shimadzu, Duisburg, Germany). Non-specific turbidity was corrected by subtracting the absorbance at 600 nm from that at 532 nm. Malondialdehyde concentration was calculated using its molar extinction coefficient of 155 mM^-1 cm^-1. The results were expressed in nmol of Malondialdehyde per gram of fresh tissue, using the appropriate equation (iii)

$$MDA\ Content\ = Vol.of\ Extraction\ Buffer\ x\ Vol.of\ Supernatant\ (ml)\frac{\left[\frac{Abs\ 532-Abs\ 600}{155}\right]1000}{(Quantity\ of\ Sample)}$$
……………………………………………………………………………………Equation iii

Where 600 nm is the wavelength for correcting non-specific turbidity while, 532 nm is the maximum absorbance wavelength of the TBA-MDA complex.

All the measurements from the testis tissue homogenate were carried out in triplicate.

Measurement of enzymatic antioxidants in the testis

Each testis sample (0.2 g) was homogenized on ice in 1.2 mL of buffer containing 100 mM potassium phosphate buffer (pH 6.8), 0.2 mM EDTA, and polyvinylpyrrolidone (PVP) using a pestle and mortar. The mixture was then centrifuged at 5,000 xg for 15 min at 4℃. The supernatant was used for assaying catalase and peroxidase enzyme activities.

Catalase (CAT) activity was measured following the protocol by Lall *et al*. [30]. In a reaction tube, 1 mL of 50 mM PBS (pH 7.2) and 1 mL of 20 mM hydrogen peroxide (H2O2) were combined. The reaction was started by adding 50 µL of crude enzyme extract, and the reduction in absorbance from H2O2 hydrolysis was monitored for 2 min at 240 nm. CAT activity was calculated using equation (iv).

$$Enzyme\ activity\ (Units/L) = \frac{(\Delta Abs \times Total\ assay\ volume)}{(\Delta t\ x\ \varepsilon\ x\ l\ x\ Enzyme\ sample\ volume)} \text{...............} \textit{Equation iv}$$

Where: Δt is the time of incubation (min), ΔAbs is the change in absorbance, ε is the Molar extinction coefficient of substrates in units of M^{-1} cm^{-1}), and l is the cuvette diameter (1 cm).

Enzyme activity (Unit) was defined as the amount of enzyme that oxidized 1µmol of substrate/min.

Peroxidase (POD) activity was conducted following the protocol described by Lall *et al* [30]. The assay was started by mixing 50 µL of tissue homogenate with 2 mL of a solution containing 15 mM guaiacol, 20 mM hydrogen peroxide, and 50 mM sodium acetate buffer (pH 7.0). The activity was measured by monitoring the increase in absorbance at 470 nm, caused by the formation of tetra guaiacol. The molar extinction coefficient of tetra guaiacol (26.6 mM–1 cm–1) was used to calculate the activity. Peroxidase enzyme activity was computed using equation (iv).

Enzyme activity (Unit) was defined as the amount of enzyme that oxidized 1 µmol of substrate/min.

Statistical analysis

Data analysis was performed using R version 4.3.1 [31] and Sigma plot version 15 [32]. Numeric data obtained from physiological and biochemical assays were subjected to one-way ANOVA,

and multiple mean comparisons were done using Tukey's Honest Significant Difference (HSD) post hoc test. Data is presented as Mean ± Standard Error of Mean (SEM). The results on mean difference were considered significant at $P < 0.05$.

Results

Molecular identification of plant sample

The obtained PCR amplicon sizes following PCR amplification were approximately 600 base pairs (bp) with *rbcL* and 500 bp with *trnH-psbA* barcode **(Supplementary Fig. S1).** A BLASTn search with the *rbcL* sequences in the database identified our query sequence with *Carissa haematocarpa* (JQ014161.1), *Carissa bispinosa* (AJ419738.1), and *Carissa bispinosa* (JQ014160.1), with percent identities of 99.7%, 99.7%, and 99.56%, respectively. While *trnH-psbA* identified query sequence with *Carissa spinarum* species, with percent identities ranging from 95.15% to 99.23% (**Supplementary Table S1**).

Bayesian phylogenetic inference approach was employed to furthure understand genetic identity considering clustering based on sequence similarities. For the *rbcl* sequences (indicated by blue colour), query sequence, clustered with *Carissa bispinosa, (*AJ419738.1) supported by a low posterior probability value of 69%. Based on the *trnH-psbA* barcode (indicated by red colour), the query sequence clustered with the three reference *C. spinarum* species (KR735499.1, KR735438.1, KR735402.1) with a posterior probability value of 99%, thus being identified as *Carissa spinarum* (**Supplementary Fig. S2**).

Phytochemical analysis of *C. spinarum* leaf and root extracts

Total Phenolic Content (TPC) in *C. spinarum* root extracts was significantly highest in acetone (130.39 ± 4.45 µg GAE/g), with other polar solvents (ethanol, methanol, Hydromethanol (H.M), Hydroethanol (H.E), distilled water, and hot distilled water) also extracting high phenolics. Hexane extracted a significantly lower concentration of phenolics (36.95 ± 0.16 µg GAE/g) ($p < 0.05$). TPC in the leaves showed a similar solvent pattern as for the root, with acetone yielding the highest (174.86 ± 1.73 µg GAE/g) and hexane the lowest (19.03 ± 0.31 µg GAE/g). Acetone also produced the highest amounts of Total Flavonoid Content (TFC) in root samples (0.98 ± 0.01 mg RE/g), while Hydromethanol (70%) showed the lowest TFC (0.02 ± 0.00 mg RE/g) ($p < 0.05$). In leaf samples, TFC was significantly higher in all polar solvents (**Table 1)**.

Table 1. Summary of TPC, TFC, TTC and TAC, in *C. spinarum* roots and leaves in different extraction solvents.

**Solvent of extraction**	**Total Phenol Concentration (µg Gallic Acid Equivalents/g Dry plant material)**		**Total Alkaloid Content (mg atropine equivalents/g of dry plant material)**		**Total Flavonoid Content (mg Rutin Equivalents/g Dry plant material)**		**Total Tannin Content (mg Tannic Acid Equivalents/g of Dry plant material)**	
	Roots	***Leaves***	***Root extracts***	***Leaf Extracts***	***Roots***	***Leaves***	***Roots***	***Leaves***
**Ethanol**	109.24 ± 0.93^{b}	157.57 ± 1.29^{b}	0.147 ± 0.002^{b}	0.235 ± 0.002^{a}	0.51 ± 0.01^{b}	2.84 ± 0.02^{ab}	0.177 ± 0.01^{ab}	0.254 ± 0.02^{b}
**Methanol**	114.45 ± 1.17^{b}	165.91 ± 1.27^{ab}	0.128 ± 0.000^{c}	0.220 ± 0.000^{b}	0.90 ± 0.01^{a}	2.87 ± 0.01^{a}	0.191 ± 0.01^{ab}	0.502 ± 0.01^{a}
**H.M (70% v/v)**	107.99 ± 1.03^{b}	147.16 ± 0.55^{c}	0.024 ± 0.000^{d}	0.034 ± 0.000^{d}	0.02 ± 0.00^{d}	2.71 ± 0.02^{ab}	0.073 ± 0.01^{cd}	0.056 ± 0.02^{d}
**H.E (70% v/v)**	109.86 ± 0.30^{b}	147.78 ± 1.88^{c}	0.013 ± 0.000^{e}	0.020 ± 0.000^{e}	0.08 ± 0.01^{d}	2.49 ± 0.05^{ab}	0.145 ± 0.00^{ab}	0.202 ± 0.00^{bc}
**n-Hexane**	36.95 ± 0.16^{c}	19.03 ± 0.31^{e}	0.011 ± 0.000^{e}	0.014 ± 0.000^{e}	0.24 ± 0.04^{c}	1.19 ± 0.07^{c}	0.012 ± 0.00^{d}	0.027 ± 0.00^{d}
**D.W**	113.82 ± 0.26^{b}	141.74 ± 0.39^{c}	0.009 ± 0.000^{e}	0.015 ± 0.000^{e}	0.37 ± 0.01^{bc}	2.47 ± 0.02^{b}	0.130 ± 0.01^{bc}	0.209 ± 0.00^{bc}
**Acetone**	130.39 ± 4.45^{a}	174.86 ± 1.73^{a}	0.026 ± 0.000^{d}	0.231 ± 0.001^{a}	0.98 ± 0.01^{a}	2.59 ± 0.08^{ab}	0.128 ± 0.02^{bc}	0.174 ± 0.00^{c}
**D.W***	114.14 ± 0.33^{b}	114.24 ± 0.48^{d}	0.172 ± 0.000^{a}	0.151 ± 0.001^{c}	0.50 ± 0.02^{b}	2.86 ± 0.01^{a}	0.205 ± 0.01^{a}	0.218 ± 0.00^{bc}

Data are presented as mean ± Standard Error of the Mean (SEM). Values sharing the same superscript within a column are not significantly different ($p > 0.05$). D.W = Distilled water; D.W* = distilled water (hot maceration method used); H.E = Hydroethanol (70% volume/volume); H.M = Hydromethanol (70% volume/volume). Note that ethanol, acetone, methanol, and distilled water generally extract high concentrations of phenols, flavonoids, tannins, and alkaloids from both roots and leaves.

The aqueous (hot maceration) extracts showed the highest tannin concentration in *C. spinarum* roots (0.205 ± 0.01 mg TAE/g), with hexane recording significantly lower tannin level in roots (0.012 ± 0.00 mg TAE/g) ($p < 0.05$). In the leaves, methanolic extracts had the highest tannin content (0.502 ± 0.01 mg TAE/g), while hexane had the lowest Total Tannin Content (TTC) (0.027 ± 0.00 mg TAE/g) ($p < 0.05$). The alkaloid concentration in root samples was highest in distilled water (hot) (0.172 ± 0.00 mg AE/g), while roots macerated with cold distilled water had the lowest alkaloid levels (0.009 ± 0.00 mg AE/g). On the leaves, ethanol extracted significantly higher alkaloids (0.235 ± 0.00 mg AE/g) ($p <0.05$), with hexane showing the lowest alkaloid content in the leaves (0.014 ± 0.00 mg AE/g) (**Table 1).**

Comparison of phytochemical concentrations in *C. spinarum* leaf and root extracts

The results showed that total phenolic content, flavonoids, tannins and alkaloids were significantly high in the leaves compared to the roots in the following solvents; acetone, cold distilled water, Hydroethanol, Hydromethanol, ethanol, and methanol ($p < 0.05$) (**Supplementary Fig. S3)**.

In vitro antioxidant activity of *C. spinarum* root and leaf extracts

In the root samples, ferric reducing power was significantly higher in Hydroethanol (6.66 ± 0.02), with hexane showing the lowest ferric reducing power (3.15 ± 0.02), **Table 2**. For the leaf extracts, Hydromethanol had the highest ferric-reducing capacity (10.88 ± 0.07), while hexane showed the lowest ferric-reducing capacity ($p < 0.05$), **Table 2**. The results for hydrogen peroxide (H_2O_2) scavenging activity in the root samples indicated that the acetone extract had significantly higher scavenging activity (34.18 ± 0.26), and hexane had the lowest H_2O_2 scavenging activity (9.70 ± 0.35), ($p < 0.05$). On the leaf extracts, ethanol demonstrated the highest H_2O_2 scavenging activity (43.53 ± 0.50), whereas hexane exhibited the lowest (18.54 ±

0.28), ($p < 0.05$) (**Table 2**, **Supplementary Fig. S5**). Pairwise comparison of ferric reducing power between the leaf and root extracts showed that ethanol, methanol, Hydromethanol, Hydroethanol, acetone, cold distilled water, and hot distilled water had significantly higher ferric antioxidant reducing capacity in the leaf samples compared to root samples ($p < 0.05$). However, there was no significant difference in ferric reducing power in hexane extracts (**Supplementary Fig. S3)**. H_2O_2 scavenging activity was significantly higher in the leaf samples among the following solvents of extraction: ethanol, methanol, Hydroethanol, Hydromethanol, and acetone. The roots in hot distilled water showed significantly higher H2O2 scavenging activity compared to the leaves ($p < 0.05$) (**Supplementary Fig. S4)**.

Table 2. Effects of *C. spinarum* extracts on FRAP and HPRS activities

	**Ferric Reducing Antioxidant Power (FRAP) ($\times 10^{-1}$)**		**Hydrogen Peroxide Scavenging Activity (%)**	
Solvent of Extraction	*Roots*	*Leaves*	*Roots*	*Leaves*
**Ethanol**	5.02 ± 0.01^{de}	9.82 ± 0.09^{c}	27.02 ± 0.21^{d}	43.53 ± 0.50^{b}
**Methanol**	5.08 ± 0.02^{de}	9.82 ± 0.09^{c}	30.44 ± 0.14^{c}	45.78 ± 0.17^{b}
**Hydro-methanol**	5.65 ± 0.03^{c}	10.88 ± 0.07^{b}	15.17 ± 0.27^{f}	27.78 ± 0.38^{d}
**Hydro-ethanol**	6.66 ± 0.02^{b}	10.13 ± 0.16^{bc}	15.70 ± 0.26^{f}	19.79 ± 0.49^{e}
**Hexane**	3.15 ± 0.02^{f}	3.13 ± 0.03^{f}	9.70 ± 0.35^{g}	18.54 ± 0.28^{e}
**Distilled Water**	5.47 ± 0.10^{cd}	6.81 ± 0.07^{de}	22.35 ± 0.41^{e}	19.59 ± 0.57^{e}
**Acetone**	4.78 ± 0.13^{e}	7.14 ± 0.09^{d}	34.18 ± 0.26^{b}	36.74 ± 0.30^{c}
**Distilled water***	5.40 ± 0.05^{cd}	6.12 ± 0.06^{e}	30.80 ± 0.20^{c}	30.90 ± 0.81^{d}
**Vitamin C**	11.85 ± 0.08^{a}	11.85 ± 0.08^{a}	77.11 ± 0.46^{c}	77.11 ± 0.46^{a}

Data shown as mean ± SEM, and those with identical superscripts within a column are not significantly different ($P > 0.05$). Cold maceration was used except for (Distilled water*), where hot maceration was the method employed for extraction.

Correlation analysis and principle component analysis to determine effective solvents

In the root samples, TPC, TFC, TAC, and TTC had a strong positive correlation with HPRS ($p < 0.05$), while only TPC and TTC showed a strong positive correlation with FRAP. Alkaloid concentration showed a weak positive correlation with FRAP ($P > 0.05$). Leaf extracts had all metabolite concentrations positively correlated with both FRAP and HPRS **(Supplementary Table S3).** For the root and leaf extracts, a similar pattern was observed where hot distilled water, ethanol, methanol, and acetone exhibited unified clustering with at least one of the variables. H.E and H.M showed positive correlation; however, they were not closely associated with any of the variables, while hexane became an outlier (**Supplementary Fig. S5**).

GC-MS results of *C. spinarum* leaf and roots, extracted with acetone and ethanol

A total of 24 and 18 volatile compounds were detected in ethanolic and acetonic leaf extracts, respectively (**Fig. 1A and B)**. On the root extracts, ethanolic and acetonic extracts had 21 and 19 compounds detected, respectively (**Fig. 1C and D**). Compounds with previous reports of antioxidant activity and male fertility modulation included, squalene [33], lupeol [34], phytol [35], Urs-12-en-3-ol, acetate, (3.beta.)- [36], Olean-12-en-3-ol [37], stigmasterol (38), α-Amyrin / β-Amyrin [39] and Vitamin E [40,41] (**Supplementary Table S2**).

Fig. 1. GC - MS Spectrograms showing peaks of A (acetone leaf extract), B (ethanol leaf extract), C (acetone root extract), and D (ethanol root extracts). The peaks are annotated with their respective numbe

Mineral content analysis in *C. spinarum* leaf and roots

Among the analyzed macrominerals, calcium had the highest abundance in only the roots (57.08 ± 0.146), while potassium reported significantly high concentrations (ppm) in both leaf and root samples (50.01 ± 0.003 and 217.25 ± 0.536, respectively) ($p < 0.05$). Sodium had the lowest concentrations (ppm) in both root and leaf samples (2.02 ± 0.019 and 1.89 ± 0.049, respectively) ($p < 0.05$), as shown in **Table 3.** For trace elements, silicon recorded the highest concentrations in both leaf and root samples (57.45 ± 0.322 and 23.95 ± 3.481, respectively), Conversely, chromium (0.02 ± 0.003) had the lowest concentrations in root and leaf samples, ($p < 0.05$), **Table 3**. Boron, a non-essential mineral, predominantly exhibited high concentrations in both leaf and root samples, while barium showed the lowest in both leaf and root samples ($p < 0.05$). A pairwise comparison of each mineral analyzed revealed that the leaves contained significantly higher concentrations of K, Mg, Cu, Zn, P, S, Si, Mn, Fe, B, and Ba, as presented in **Table 3**.

Table 3. Mineral content of *C. spinarum* leaf and root samples.

Mineral	Concentration in *C. spinarum* roots (ppm)	Concentration in *C. spinarum* leaves (ppm)	Root and leaf pairwise comparison (p-values)
**Macrominerals**			
Ca	57.08 ± 0.146^{a}	18.93 ± 0.493^{d}	0.00064
K	50.01 ± 0.003^{a}	217.25 ± 0.536^{a}	0.00029
Mg	32.53 ± 2.234^{b}	75.37 ± 1.410^{b}	0.00289
S	8.28 ± 0.271^{c}	13.03 ± 0.443^{d}	0.00710
P	5.40 ± 0.224^{d}	26.77 ± 0.378^{c}	0.00079
Na	2.02 ± 0.019^{d}	1.89 ± 0.049^{e}	0.14277
**Trace elements**			
Se	16.74 ± 0.148^{a}	9.86 ± 0.150^{b}	0.00249
Si	23.95 ± 3.481^{a}	57.45 ± 0.322^{a}	0.02569
Fe	3.58 ± 0.395^{b}	9.46 ± 0.099^{b}	0.01492
Zn	± 0.139^{c}	1.15 ± 0.152^{d}	0.71568
Mn	0.50 ± 0.026^{c}	5.71 ± 0.322^{c}	0.01197
Cu	0.13 ± 0.008^{c}	0.19 ± 0.014^{d}	0.03100
Mo	0.03 ± 0.002^{c}	0.04 ± 0.011^{d}	0.78918
Cr	0.02 ± 0.003^{c}	0.13 ± 0.017^{d}	0.05336
**Non-Essential Minerals**			
B	0.18 ± 0.01^{a}	1.64 ± 0.013^{a}	0.00033
Ba	0.05 ± 0.01^{b}	0.35 ± 0.017^{b}	0.00729

Data are presented as mean ± SEM. For each mineral category (macrominerals, trace elements, and non-essential minerals), the concentration values on leaves and roots with the same superscripts are not significantly different at the 5% significance level. Mineral concentration was measured in parts per million (ppm); n = 3.

Effects of *C. spinarum* aqueous root extract on reproductive organ and body weights

The assessment of testicular weight index showed a significant decrease in the negative control group receiving metronidazole alone ($p<0.05$). In contrast, the extract-treated groups, especially those given low and medium doses, showed a significant increase in relative testis weight index (0.95% and 0.98%, respectively) compared to the negative control (0.28%; $p<0.05$). The relative testicular weights in the low and medium extract-treated groups were not significantly different from those in the normal control group (1.015%; $p>0.05$). Similarly, cauda epididymis weights were significantly reduced among the negative control group (0.28g; $p<0.05$). Conversely, the low and high-dose extract-treated groups exhibited increased epididymal weights (0.45g, and 0.45 g, respectively), which were not significantly different from the positive and normal control groups (0.52g and 0.55 g, respectively), ($p>0.05$) (**Table 4, Supplementary Fig. S8)**

Table 4. Summary table indicating the effects of *C. spinarum* aqueous root extract on total sperm count, viability, morphology, testis weight index and epididymis weight

Groups	n	Sperm Count ($\times 10^6$) per mL of the suspension.	Viable Sperm cells (%)	Spermatozoa with normal Morphology (%)	Testis Weight Index (%)	Total epididymal Weight (g)	Malondialdehyde (MDA) (nmoles/g of tissue sample	Catalase enzyme Activity (nmol g^{-1} testis tissue)$\times 10^{-2}$	Peroxidase Enzyme Activity (nmol g^{-1} testis tissue) $\times 10^{-2}$
**1**	5	55.00 ± 1.21^{ab}	86.60 ± 1.43^{a}	85.50 ± 1.23^{a}	1.015 ± 0.01^{a}	0.55 ± 0.02^{a}	0.88 ± 0.08^{c}	15.19 ± 0.09^{a}	19.87 ± 0.13^{b}
**2**	5	3.49 ± 0.46^{d}	68.60 ± 0.54^{b}	4.30 ± 0.92^{d}	0.45 ± 0.01^{b}	0.28 ± 0.01^{c}	5.35 ± 0.06^{a}	8.45 ± 0.09^{b}	17.26 ± 0.11^{b}
**3**	5	35.03 ± 2.91^{bc}	88.50 ± 1.62^{a}	41.90 ± 4.77^{bc}	0.95 ± 0.10^{a}	0.45 ± 0.02^{ab}	3.89 ± 0.50^{ab}	13.88 ± 0.21^{a}	28.07 ± 0.24^{ab}
**4**	5	37.20 ± 3.86^{abc}	86.70 ± 1.42^{a}	60.50 ± 3.09^{ab}	0.98 ± 0.02^{a}	0.39 ± 0.01^{bc}	1.38 ± 0.14^{c}	14.56 ± 0.19^{a}	30.95 ± 0.11^{ab}
**5**	5	11.66 ± 1.62^{cd}	70.50 ± 2.90^{b}	32.60 ± 3.83^{c}	0.78 ± 0.04^{ab}	0.43 ± 0.02^{ab}	2.77 ± 0.23^{bc}	10.79 ± 0.22^{ab}	33.24 ± 0.14^{ab}
**6**	5	64.43 ± 4.82^{a}	88.20 ± 1.18^{a}	83.20 ± 1.49^{a}	0.70 ± 0.02^{ab}	0.52 ± 0.01^{ab}	1.03 ± 0.19^{c}	13.30 ± 0.18^{a}	38.52 ± 0.10**a**

Effect of *C. spinarum* root extract on sperm parameters and biochemical antioxidant markers in rats after 58 days of treatment. Note that at low and medium extract doses, there was an improvement in sperm characteristics, increased testicular weight index, cauda epididymis weights, and selected biochemical antioxidant markers compared to the negative control. (G1 = Distilled water, G2 = Metronidazole 400 mg/kg bw, G3 = 400 mg/kg bw Metronidazole + 250 mg/kg bw extracts, G4 = 400 mg/kg bw Metronidazole + 500 mg/kg bw extracts, G5 = 400 mg/kg bw Metronidazole + 1000 mg/kg bw extracts, G6 = 400 mg/kg bw Metronidazole + 400 mg/kg bw Vitamin E + 0.36 mg/kg bw Sustanon weekly injection). Data are presented as Mean ± SEM; values with the same superscript within a column are not significantly different ($P > 0.05$). n = number of animals.

Effects of *C. spinarum* aqueous root extract on sperm qualities

The administration of metronidazole alone in the negative control (group 2) led to a significant decrease in sperm concentration 3.49 × 10^6/mL) and viability 68.6%, and consequently lowered the levels of morphologically normal sperm cells to only 4.3%. However, treatment with escalating doses of the root extracts significantly ($p < 0.05$) improved these parameters especially on the low and medium doses of the extracts where sperm concentration was 35.03 × 10^6/mL and 37.2 × 10^6/mL respectively, viability 88.5% and 86.7% respectively, while the amounts of sperms with normal morphology were high in these groups (41.90% and 60.5%, respectively). The medium extract dose showed sperm parameters that were not significantly ($p > 0.05$) different from those of the normal and positive controls. At a high dose of the root extracts, there was a significant reduction ($p < 0.05$) in sperm parameters compared to all other treatment groups, including the positive and normal controls, but not significantly different from the negative control (**Table 4, Supplementary Fig. S9, S10**).

Testicular histology

In the normal control group, the testicular parenchyma consisted of regularly shaped seminiferous tubules, which appeared bounded by an intact basement membrane supporting spermatogonia and Sertoli cells. The Sertoli cells are tall and seem to span the entire diameter of the seminiferous epithelium, with apical villi extending into the lumen, while lateral recesses support and contain dividing and differentiating germ cells. The tubular lumen contained spermatozoa with tails pointing toward the center of the lumen and heads attached to the apical villi of the Sertoli cells. The spermatogenic cells appeared to develop in groups at the same stage toward the lumen, indicating a normal spermatogenic cycle and wave. The interstitial tissue of the normal control displayed interstitial cells, blood vessels, and lymphatic vessels. Among these interstitial cells are Leydig cells, which appeared polyhedral in shape (**Fig. 2A**).

The negative control, which was treated with metronidazole 400 mg/kg/bw alone, showed significant structural changes in the seminiferous tubules. The seminiferous epithelium generally appeared thinner with prominent vacuolation in spermatogenic cells and a lack of Sertoli cells. There was evidence of complete seminiferous tubular degeneration, with dissociation from the basement membrane, which appeared irregular and showed cell exfoliation in some areas. The interstitial tissue appeared abnormally enlarged, with signs of edema and irregular distribution of interstitial cells (**Fig. 2B).**

Seminiferous tubules in animals treated with low and medium doses of extract exhibited normal structural features, including an intact germinal epithelium and basement membrane. The germinal epithelium within the seminiferous tubules was characterized by well-organized spermatogenic cells at various stages of development, intermingled with Sertoli cells and spermatozoa (**Fig. 2C and D).**

At high extract dose treatment, the basement membrane of seminiferous tubules appeared irregularly shaped with folds in certain areas (**Fig. 2C and D**), and the seminiferous epithelium seemed dissociated from the basement membrane and from each other. There was evidence of cellular degeneration, as indicated by pyknotic nuclei in the affected cells. The seminiferous epithelial cells were also exfoliated from the basement membrane (**Fig. 2E**).

The seminiferous tubules in the positive control group exhibited features of seminiferous epithelium resting on an intact basement membrane, with a lumen filled with spermatozoa, indicative of a normal seminiferous tubule (**Fig. 2F**).

Fig. 2 Photomicrographs of rat seminiferous tubules. **A**) Normal control with normally aligned spermatogenic cells at different stages of development interspersed among Sertoli cells (SC) lying on intact basement membrane (BM) and whose apical villi attach the elongate spermatids (ES) and appear to project into the lumen (L). **B**) Negative control subjected to metronidazole alone showing significantly disorganized seminiferous epithelial alignment within the seminiferous tubules with accompanying increased luminal diameter (L) that appears empty. Vacuolations can also be seen (thick blue arrow), **C** and **D**) represent low and medium dose of *C. spinarum root* extracts, respectively. The seminiferous tubular integrity in both treatment groups appear intact with the cellular ultrastructure resembling that of the normal control. **E**) represent the high dose extract treated group. Note the loss of seminiferous epithelial integrity characterised by disintegration and general swelling of the seminiferous epithelial diameter. The basement membrane (BM) appears irregular, and the lumen (L) is empty. **F**) represents the positive control, its seminiferous tubular ultrastructure resembles that of normal control with intact BM and spermatogenic cells at different stages of development interspersed among the Sertoli cells (SC) with spermatozoa (S) in the lumen. The interstitium of positive control group also shows cellular outline comprising of Leydig cells (LC) among other interstitial cells and blood vessel (BV). Haematoxylin and eosin stain used and the magnification bar represents 50 µm.

Effects of *C. spinarum* aqueous root extract on oxidative stress markers and enzymatic antioxidants in the testis

To understand the *in vivo* antioxidant properties of aqueous root extracts of this plant in relation to male infertility treatment, testicular levels of malondialdehyde (MDA) and the activity of peroxidase and catalase were measured in right testis homogenates. The negative control group, which was treated with metronidazole alone, showed the highest concentration of MDA (5.35 ± 0.69 nanomoles/g of testicular tissue) ($p<0.05$). The treatment groups, especially those given medium (500 mg/kg) and high (1000 mg/kg/bw) extract doses, exhibited significantly lower MDA levels of 1.38 ± 0.14 and 2.77 ± 0.23 nanomoles/g of testicular tissue ($p<0.05$). The animals treated with medium and high extract doses showed MDA levels that were not significantly different from those in the positive and normal control groups ($p > 0.05$) (**Table 4**).

The results for catalase enzyme activity showed a significant reduction among the negative control group (8.45 ± 0.09 nmol g^{-1} testis tissue × 10^{-2}) treated with metronidazole alone. However, treatment with low, and medium doses lead to a significant ($p <0.05$) increase in the levels of catalase enzyme activity of 13.88 ± 0.21 and 14.56 ± 0.19 nmol g^{-1} testis tissue × 10^{-2}, respectively. The results also indicated that those MDA levels from the low and medium extract treated animals were not significantly different from the normal and positive control group animals ($p>0.05$) (**Table 4**).

The test for peroxidase enzyme activity reported normal levels of the enzyme activity in the negative control animals (17.26 ± 0.11 nmol g^{-1} testis tissue × 10^{-2}) with reference to the normal control group ($p>0.05$). However, there was an increase in the activity of peroxidase enzyme activity among the low (28.07 ± 0.24 nmol g^{-1} testis tissue × 10^{-2}), medium (30.95 ± 0.11 nmol g^{-1}

testis tissue × 10^{-2}) and high (33.24 ± 0.14 nmol g^{-1} testis tissue × 10^{-2}) extract treated groups. This increase in peroxidase enzyme activity among the extract treated groups was not significantly different with reference to the positive control animals ($p>0.05$) (**Table 4**)

Discussion

Plants within the same family exhibits similar morphological features, calling for DNA barcoding in discrimination due to its robustness and high level of accuracy. In this study, *trnH-psbA* authenticated the identity of the plant species as *Carissa spinarum* L. following BLASTn search and phylogenetic analysis. The *trnH-psbA* barcode exhibited clustering of the query sequence with other database sequences belonging to *Carissa spinarum* species supported with a high posterior probability of 99% leading to the agreement on plant species to be *C. spinarum*. Similar results supporting the accuracy and efficiency of *trnH-psbA* in species discrimination were also reported by Kress *et al*. [42].

It was evident that acetone, hot distilled water, methanol, and ethanol, generally extracted higher amounts of phenolics, flavonoids, alkaloids, and tannins in both leaf and root samples, confirming their presence in *C. spinarum* leaf and root samples in high amounts. The present study therefore gives insights on to the availability of highly soluble phenols, flavonoids, alkaloids, and tannins in *C. spinarum* root and leaf samples. These compounds have been shown to exhibit various pharmacological actions and therefore supports the previous findings on the use of *C. spinarum* in the treatment of various diseases such as chest pains, viral infections, male infertility, malaria, and inflammatory diseases [43]. A comparison of the leaf and roots revealed generally high phenol, flavonoid, tannins, and alkaloids content in the leaf extracts in most solvents compared to the roots. In most vegetative plants, phenols, flavonoids, alkaloids and tannins are normally synthesized and concentrated in the leaves, suiting their roles in protection against ultraviolet radiations, defence against pathogens, parasites, and herbivorous animals [44]. The ferric reducing antioxidant power and hydrogen

radical scavenging capacity of *C. spinarum* root and leaf extracts were high in methanol, ethanol, acetone, and hot distilled water, suggesting high solubility of antioxidant compounds present in *C. spinarum* in polar organic solvents and in hot distilled water. The potent antioxidant activity and diverse phytocompounds exhibited by *C. spinarum* support its effectiveness in treating oxidative stress-related pathologies, inflammatory diseases, and microbial infections [43].

Pearson's correlational analysis and principle component analysis (PCA) were utilized to scrutinize the influence of phytocompounds concentration on antioxidant activity and most effective extraction solvents, respectively. On the root extracts, phenols, tannins, alkaloids positively contributed and were key determining factors to FRAP and HPRS. Antioxidant activities in the leaf extracts were contributed to by all the secondary metabolites assayed. This is consistent with previous findings on the positive contribution of different classes of SM to radical scavenging [13,45]. PCA revealed acetone, methanol, ethanol, and hot distilled water as the most effective solvents with a similar extraction pattern, observed through clustering, in both the roots and leaf samples of *C. spinarum*. This information is pivotal for selecting an appropriate solvent for extracting targeted groups of compounds and for assessing antioxidant activities.

Volatile compound detection indicated that the highest number of volatile compounds were present in ethanol, for both the leaf and root samples, indicating the suitability of ethanol in dissolving most of the compounds in *C. spinarum.* Among the compounds detected previous studies have shown the bioactivity of squalene [33], lupeol [34], phytol [35], Urs-12-en-3-ol, acetate, (3.beta.)- [36], Olean-12-en-3-ol [37], stigmasterol [38], α-Amyrin / β-Amyrin [39]

and Vitamin E [40,41] as antioxidant and male reproductive function modulators, where they enhance spermatogenesis and protects the testicular tissues against oxidative stress based injuries. The findings from GC-MS are relevant in supporting the effects of *C. spinarum* plants in inhibition of oxidative stress mediated pathologies and infertility.

Minerals play pivotal roles in health and are thus recommended for intake through dietary sources, or direct supplementation. In the current study, results showed that both the leaves and roots were rich in Calcium, potassium, magnesium, sulphur, phosphorus, and sodium as macrominerals, and selenium which are required by the body in milligram quantities per day. Potassium and sodium ions are essential in the maintenance of electrolyte balance, with magnesium also being required as a cofactor in more than 300 metabolic reactions by the body. Calcium, phosphorus, and sulfur are needed for bone mineralization, Components of genetic information (DNA and RNA), and in cellular signalling, respectively [46]. The findings also showed that both leaf and root samples were rich in trace elements, including selenium, silicon, iron, zinc, manganese, molybdenum, and chromium. The body requires selenium, copper, iron, and zinc as antioxidants, components of cytochrome, oxygen transport, and enzymatic cofactors, respectively [46,47]. These minerals are therefore crucial for maintaining growth and development, boosting immunity, and supporting reproductive function in both males and females [46]. The leaves were found to contain a generally higher concentration of minerals than the roots; this may be linked to several biochemical and metabolic activities that are more likely to occur in the leaves than in the roots [46]. The availability of these minerals, in addition to other phytochemicals, can therefore support the

plant's previous therapeutic effects in traditional and modern applications, including the treatment of oxidative damage-based pathologies [47,48].

In the present study, the counteractive effects of *C. spinarum* aqueous root extracts on the oxidative stress-induced infertility of metronidazole on the testis of adult male Wistar rats was investigated. The findings on reproductive organ weight revealed a remarkable reduction in the testis weight index and cauda epididymis weight among the negative control groups, treated with metronidazole alone, possibly attributed to the previously reported necrotic and degenerative effects of Metronidazole-induced oxidative stress [5]. We also found that the co-administration of *C. spinarum* aqueous root extract ameliorated the effect of Metronidazole, and the tail epididymis and relative testis body weight was significantly increased and were similar to the positive and normal controls. These effects can be attributed to the protective capacity of the secondary metabolites present in the aqueous root extracts. Similar results were reported by Al-Alami et al. [10], who demonstrated the use of plant extracts to counter Metronidazole-induced changes in the testis of male Wistar rats.

Evaluation of sperm parameters showed a remarkable reduction in sperm concentration, viability, and morphological alterations in the negative control, which received metronidazole alone, might have been due to increased testicular cytotoxic activities of metronidazole [49]. This could either interfere with gene expression of testicular steroidogenic enzymes, synergizing spermatogenesis, downregulate receptor binding of pituitary gonadotropins on Sertoli cells, or directly affect sperm DNA via lipid peroxidation or a combination of some or all of these factors, hence impaired spermatogenesis. However, reversal on this measure was

achieved in low and medium-dose extract-treated groups, showing the potential amelioration of testicular tissue damage, owing to oxidative stress effects of metronidazole, by the extract. Previous studies on phytochemical analysis of *C. spinarum* root extracts, its therapeutic benefits were ascribed to high antioxidant activity as a result of high concentration of phenols, tannins, flavonoids and alkaloids [43]. These plant phytocompounds are also known to improve sperm characteristics, as well as to strike the balance between ROS production and scavenging potential, during oxidative stress [50]. Some reports have shown improvement of antioxidant, spermatogenic and steroidogenic enzyme activities [10]. From the foregoing, it is possible that these compounds acted synergistically or individually to restore sperm count, morphology and viability. In a similar study on fertile and infertile men, it was reported that secondary metabolites in medicinal plants improve fertility by boosting reproductive hormone action and improving various sperm characteristics, including morphology, vitality, concentration, and motility [51,52].

Testicular histology revealed seminiferous tubule with normal structural features at low and medium-dose treatment groups that were similar to the normal and positive controls. This was probably due to the ameliorative effects of *C. spinarum* on oxidative stress-mediated testicular tissue damage induced by metronidazole. The successful spermatogenesis greatly depends on the testicular parenchyma integrity, where key cells such as the interstitial endocrine cells, germ cells, and sustentacular cells within the seminiferous epithelium play key roles [53]. Alteration of the germinal epithelium as well as the interstitial endocrine cells are known to impair spermatogenesis [54], as observed in the negative control and high dose extract group. Vacuoles on seminiferous tubular epithelium have been ascribed to impaired

spermatogenesis as was reported by in rabbits treated with high dose of khat extracts [55] and vervet monkeys treated with high dose of (-)-cathinone [56].

The findings on histology support the results obtained on sperm characteristics and reproductive organ weights in this study. In low and medium-dose treatment groups, normal spermatogenesis was observed and this explains the high number of spermatozoa, viability and normal morphology as compared to the negative control. This also translated to higher cauda epididymal weights, since 50% of the tail epididymis weight is attributed to spermatozoa [10]. The observations from testicular histology also showed that the low- and medium-dose extract-treated groups had normal testicular contents. This might have accounted for the higher absolute testicular weights compared to the negative control, whose seminiferous tubules were largely void of cells that are part of the testicular content. The variance in measured sperm parameters among treatment groups and the positive control, despite similar morphological features, remains an area for further research. Perhaps more detailed ultrastructural studies may offer more information on cytological effects and this is an area for future studies.

Test on malondialdehyde concentration revealed high MDA level in negative control confirming the contribution of metronidazole in increasing oxidative stress by enhancing lipid peroxidation. Reactive oxygen species enables the abstraction of hydrogen, replacing it with oxygen atoms and forming lipid peroxyl radicals and hydroperoxides. These molecules interfere with the membrane's integrity and also react with other biomolecules like proteins, thus degenerating physiological and biochemical processes charged with maintaining fertility [50], as seen in the negative control group of the present study. However, reduction of the

radicals was achieved in low and medium-dose extract-treated groups, confirming the extract's *in vivo* antioxidant potential.

Test on antioxidant enzymes essential for maintenance of redox cycle revealed a significant reduction in the activity of catalase enzyme in the negative control group, which received metronidazole alone. The cumulative deleterious effects of metronidazole on the testicular tissues might have resulted to this as previously reported by [49]. The increase in catalase enzyme activity in extract-treated groups, similar to the normal and positive control shows the effects of *C. spinarum* aqueous root extract in alleviating the catalase activity inhibition resulting from metronidazole administration. However, the reversal groups with the effects of bioactive compounds in the extract may have mediated the restoration of catalase activity by adding to the radical scavenging activities. Activity of peroxidase enzyme was not altered upon administration of metronidazole alone with reference to the normal control animals. This suggests that metronidazole-induced oxidative damage does not have inhibition effects on peroxidase enzyme activity. However, administration of the extract enhanced peroxidase activity among the treatment groups, similar to the positive control. This confirms the *in vivo* antioxidant contribution of extract's bioactive compounds. This could also reveal possible similarity in mechanism, between *C. spinarum* aqueous root extracts and vitamin E, an antioxidant on increasing catalase and peroxidase activities, in restoring testicular tissue redox balance.

Conclusions

The findings from this study confirmed that oxidative stress-mediated infertility in adult male Wister rats can be reversed using aqueous root extracts from *C. spinarum*. This was supported by improved sperm qualities and alleviation of oxidative stress in the animal groups treated

with aqueous root extracts of *C. spinarum*. The finding therefore substantiates the potential of extrapolation of this plant's herbal preparation in restoration male infertility attributed to ROS damage in human, pending further investigations. From our knowledge, this is the first report demonstrating the ameliorative effects of *Carissa spinarum* on oxidative stress-induced male infertility. By isolating and screening single compounds, as well as investigating their specific molecular pathways, we can understand intricate details of this plants extract's mode of action in restoring male fertility. This will pave way for the development of targeted therapeutic interventions.

Ethical Statement

Ethical clearance for animal use was obtained from the Biosafety, Animal Use and Ethics Committee of the Faculty of Veterinary Medicine (Reference number FVM BAUEC/2023/528).

Data availability

The data that supports the finding of this study are included in this article and its supplementary material. Further inquiries on raw data can be availed by the corresponding author upon request.

Funding

This research did not receive any specific grant from funding agencies in the public, commercial, or not-for-profit sectors.

Declaration of Competing Interest

The authors declare that they have no known competing financial interests or personal relationships that could have appeared to influence the work reported in this paper.

Acknowledgments

The authors would like to acknowledge the Department of Biochemistry and Department of Veterinary Anatomy and Physiology at the University of Nairobi for providing facilities to carry out this study.

References

1. Naz M, Kamal M. Classification, causes, diagnosis and treatment of male infertility: a review. Orient Pharm Exp Med. 2017 June;17(2):89–109.

2. Kumar N, Singh A. Trends of male factor infertility, an important cause of infertility: A review of literature. J Hum Reprod Sci. 2015;8(4):191.

3. Onochie C, Evi K, O'Flaherty C. Role of Redox-Induced Protein Modifications in Spermatozoa in Health and Disease. Antioxidants. 2025 June 12;14(6):720.

4. Chorosho SH, Malik N, Panesar G, Kumari P, Jangra S, Kaur R, et al. Phytochemicals: Alternative for Infertility Treatment and Associated Conditions. Alves MS, editor. Oxidative Medicine and Cellular Longevity. 2023 May 11;2023:1–21.

5. Kumari M, Singh P. Tribulus terrestris ameliorates metronidazole-induced spermatogenic inhibition and testicular oxidative stress in the laboratory mouse. Indian Journal of Pharmacology. 2015 May 1;47(3):304.

6. Allahverdiyev AM, Bagirova M, Yaman S, Koc RC, Abamor ES, Ates SC, et al. Development of New Antiherpetic Drugs Based on Plant Compounds. In: Fighting Multidrug Resistance with Herbal Extracts, Essential Oils and Their Components [Internet]. Elsevier; 2013 [cited 2022 Nov 14]. p. 245–59. Available from: https://linkinghub.elsevier.com/retrieve/pii/B9780123985392000173

7. Kyarimpa C, Nagawa CB, Omara T, Odongo S, Ssebugere P, Lugasi SO, et al. Medicinal Plants Used in the Management of Sexual Dysfunction, Infertility and Improving Virility in the East African Community: A Systematic Review. Wan C (Craig), editor. Evidence-Based Complementary and Alternative Medicine. 2023 Jan;2023(1):6878852.

8. Liu Y, Zhang Y, Muema FW, Kimutai F, Chen G, Guo M. Phenolic Compounds from *Carissa spinarum* Are Characterized by Their Antioxidant, Anti-Inflammatory and Hepatoprotective Activities. Antioxidants. 2021 Apr 23;10(5):652.

9. Muruthi CW, Ngugi MP, Runo SM, Mwitari PG. *In vitro* antioxidant activities of *Carissa edulis* (Forssk) Vahl) and *Pappea capensis* (Eckyl. & Zeyh) extracts. Heliyon. 2023 Jan;9(1):e12965.

10. Al-Alami ZM, Shraideh ZA, Taha MO. Rosmarinic acid reverses the effects of metronidazole-induced infertility in male albino rats. Reprod Fertil Dev. 2017;29(10):1910.

11. Mcclain R. Effect of metronidazole on fertility and testicular function in male rats. Fundamental and Applied Toxicology. 1989 Apr;12(3):386–96.

12. Auger J, Eustache F, Rouiller-Fabre V, Canivenc-Lavier M, Livera G. Integrative rodent models for assessing male reproductive toxicity of environmental endocrine active substances. Asian J Androl. 2014;16(1):60.

13. Pere K, Mburu K, Muge EK, Wagacha JM, Nyaboga EN. Molecular Authentication and Phytochemical Evaluation of Indigenous Germplasm of Genus Physalis for Sustainable Utilization. IJPB. 2023 Nov 10;14(4):998–1016.

14. Hall, TA. BioEdit: a user-friendly biological sequence alignment editor and analysis program for Windows 95/98/NT. Nucl. Acids. Symp. Ser. 41:95-98. 1999;

15. Huelsenbeck J, Ronquist F. MRBAYES: Bayesian inference of phylogenetic trees. Bioinformatics. 2001;17:754–755. 2001;

16. Altemimi A, Lakhssassi N, Baharlouei A, Watson D, Lightfoot D. Phytochemicals: Extraction, Isolation, and Identification of Bioactive Compounds from Plant Extracts. Plants. 2017 Sept 22;6(4):42.

17. Njau V, Maina E, Anjili C. In vitro antileishmanial activity and phytochemical analysis of Carissa edulis against Leishmania major. African Journal of Pharmacology and Therapeutics [Internet]. 2016; Available from: http://journals.uonbi.ac.ke/ajpt

18. Siddiqui N, Rauf A, Latif A, Mahmood Z. Spectrophotometric determination of the total phenolic content, spectral and fluorescence study of the herbal Unani drug Gul-e-Zoofa (Nepeta bracteata Benth). Journal of Taibah University Medical Sciences. 2017 Aug;12(4):360–3.

19. Aryal S, Baniya MK, Danekhu K, Kunwar P, Gurung R, Koirala N. Total Phenolic Content, Flavonoid Content and Antioxidant Potential of Wild Vegetables from Western Nepal. Plants. 2019 Apr 11;8(4):96.

20. Makkar HPS. Quantification of Tannins in Tree and Shrub Foliage [Internet]. Dordrecht: Springer Netherlands; 2003 [cited 2024 Feb 2]. Available from: http://link.springer.com/10.1007/978-94-017-0273-7

21. Ajanal M, Gundkalle M, Nayak S. Estimation of total alkaloid in Chitrakadivati by UV-Spectrophotometer. Ancient Sci Life. 2012;31(4):198.

22. Wang S, Gan Y, Mao X, Kan H, Li N, Zhang C, et al. Antioxidant Activity Evaluation of Oviductus Ranae Protein Hydrolyzed by Different Proteases. Molecules. 2021 Mar 15;26(6):1625.

23. Ruch RJ, Cheng S jun, Klaunig JE. Prevention of cytotoxicity and inhibition of intercellular communication by antioxidant catechins isolated from Chinese green tea. Carcinogenesis. 1989;10(6):1003–8.

24. Ngigi AN, Muraguri BM. ICP-OES determination of essential and non-essential elements in Moringa oleifera, Salvia hispanica and Linum usitatissimum. Scientific African. 2019 Nov;6:e00165.

25. oecd-ilibrary.org [Internet]. [cited 2024 May 9]. Test No. 423: Acute Oral toxicity - Acute Toxic Class Method | READ online. Available from: https://read.oecd-ilibrary.org/environment/test-no-423-acute-oral-toxicity-acute-toxic-class-method_9789264071001-en

26. WHO laboratory manual for the examination and processing of human semen [Internet]. 2021 [cited 2024 Jan 12]. Available from: https://www.who.int/publications-detail-redirect/9789240030787

27. Kondracki S, Wysokińska A, Kania M, Górski K. Application of two staining methods for sperm morphometric evaluation in domestic pigs. Journal of Veterinary Research. 2017 Sept 26;61(3):345–9.

28. Slaoui M, Fiette L. Histopathology Procedures: From Tissue Sampling to Histopathological Evaluation. In: Gautier JC, editor. Drug Safety Evaluation [Internet]. Totowa, NJ: Humana Press; 2011 [cited 2025 Sept 11]. p. 69–82. (Methods in Molecular Biology; vol. 691). Available from: https://link.springer.com/10.1007/978-1-60761-849-2_4

29. Chen Z, Gallie DR. Dehydroascorbate Reductase Affects Leaf Growth, Development, and Function. Plant Physiology. 2006 Oct 10;142(2):775–87.

30. Lall N, Nikolova RV, Bosa AJN. Changes in activities of superoxide dismutase, peroxidase and catalase from leaves of Impatiens flanaganiae in response to light intensity. South African Journal of Botany. 1999 Aug;65(4):255–9.

31. R Core Team. A language and environment for statistical computing. R Foundation for Statistical Computing, Vienna, Austria. 2023; Available from: https://www.R-project.org/

32. Systat Software Inc. SigmaPlot for Windows (Version 15) [Software]. San Jose, CA. 2023;

33. Huang ZR, Lin YK, Fang JY. Biological and Pharmacological Activities of Squalene and Related Compounds: Potential Uses in Cosmetic Dermatology. Molecules. 2009 Jan 23;14(1):540–54.

34. Arabkarami P, Mortazavi P, Hesaraki S. Effect of lupeol on testicular lesions induced by cadmium chloride in rats. Avicenna Journal of Phytomedicine [Internet]. 2025 Jan [cited 2025 Nov 21];15(1). Available from: https://doi.org/10.22038/ajp.2024.24716

35. Santos CCDMP, Salvadori MS, Mota VG, Costa LM, De Almeida AAC, De Oliveira GAL, et al. Antinociceptive and Antioxidant Activities of Phytol *In Vivo* and *In Vitro* Models. Neuroscience Journal. 2013 June 11;2013:1–9.

36. Dong J, Ye T, Dong Y, Hui J, Wang X. Ursolic acid attenuates oligospermia in busulfan-induced mice by promoting motor proteins. PeerJ. 2024 July 5;12:e17691.

37. Zhao H, Liu J, Song L, Liu Z, Han G, Yuan D, et al. Oleanolic acid rejuvenates testicular function through attenuating germ cell DNA damage and apoptosis via deactivation of NF-κB, p53 and p38 signalling pathways. Journal of Pharmacy and Pharmacology. 2017 Mar 1;69(3):295–304.

38. Nieminen P, Pölönen I, Ikonen K, Määttänen M, Mustonen AM. Evaluation of reproductive safety of β-sitosterol on the American mink (Neovison vison). Chemosphere. 2008 Mar;71(3):493–9.

39. Watcho P, Zelefack F, Ngouela S, Nguelefack TB, Kamtchouing P, Tsamo E, et al. Enhancement of erectile function of sexually naïve rats by β–sitosterol and α–β–amyrin acetate isolated from the hexane extract of Mondia whitei. Asian Pacific Journal of Tropical Biomedicine. 2012 Jan;2(3):S1266–9.

40. Majzoub A, Agarwal A. Systematic review of antioxidant types and doses in male infertility: Benefits on semen parameters, advanced sperm function, assisted reproduction and live-birth rate. Arab Journal of Urology. 2018 Mar;16(1):113–24.

41. Sabetian S, Jahromi BN, Vakili S, Forouhari S, Alipour S. The Effect of Oral Vitamin E on Semen Parameters and IVF Outcome: A Double-Blinded Randomized Placebo-Controlled Clinical Trial. Taysi S, editor. BioMed Research International. 2021 Jan;2021(1):5588275.

42. Kress WJ, Wurdack KJ, Zimmer EA, Weigt LA, Janzen DH. Use of DNA barcodes to identify flowering plants. Proc Natl Acad Sci USA. 2005 June 7;102(23):8369–74.

43. Sharma N, Kumar V, Gupta N, Shekhar P, Kaur PS. Traditional Importance, Phytochemistry, Pharmacology, and Toxicological Attributes of the Promising Medicinal Herb Carissa spinarum L. Separations. 2023 Feb 24;10(3):158.

44. Ramaroson ML, Koutouan C, Helesbeux JJ, Le Clerc V, Hamama L, Geoffriau E, et al. Role of Phenylpropanoids and Flavonoids in Plant Resistance to Pests and Diseases. Molecules. 2022 Nov 30;27(23):8371.

45. Nakitto AMS, Byaruhanga YB, Wagner AE, Muyonga JH. Influence of ripeness stage on the bioactive compounds' contents and antioxidant activities of Solanum anguivi Lam. fruits accessions. Heliyon. 2023 Nov;9(11):e21614.

46. Morris AL, Mohiuddin SS. Biochemistry, Nutrients. In: StatPearls [Internet]. Treasure Island (FL): StatPearls Publishing; 2025 [cited 2025 Sept 12]. Available from: http://www.ncbi.nlm.nih.gov/books/NBK554545/

47. Bettger WJ. Zinc and selenium, site-specific versus general antioxidation. Can J Physiol Pharmacol. 1993 Sept 1;71(9):721–4.

48. Muscolo A, Mariateresa O, Giulio T, Mariateresa R. Oxidative Stress: The Role of Antioxidant Phytochemicals in the Prevention and Treatment of Diseases. IJMS. 2024 Mar 13;25(6):3264.

49. Raji Y, T. Kunle-A O, Olaleye SB, Gbadegesin MA, Awobajo FO, Osonuga OA, et al. Impact of α-tocopherol on Metronidazole and Tetracycline-induced Alterations in Reproductive Activities of Male Albino Rats. J of Biological Sciences. 2006 Dec 15;7(1):41–6.

50. Kaltsas A. Oxidative Stress and Male Infertility: The Protective Role of Antioxidants. Medicina. 2023 Oct 4;59(10):1769.

51. Chen DL, Li N, Lin L, Long H ming, Lin H, Chen J, et al. Confocal mirco-Raman spectroscopic analysis of the antioxidant protection mechanism of the oligosaccharides extracted from Morinda officinalis on human sperm DNA. Journal of Ethnopharmacology. 2014 Apr;153(1):119–24.

52. Shukla KK, Mahdi AA, Ahmad MK, Shankhwar SN, Rajender S, Jaiswar SP. Mucuna pruriens improves male fertility by its action on the hypothalamus–pituitary–gonadal axis. Fertility and Sterility. 2009 Dec;92(6):1934–40.

53. Brinster RL, Zimmermann JW. Spermatogenesis following male germ-cell transplantation. Proc Natl Acad Sci USA. 1994 Nov 22;91(24):11298–302.

54. Santi D, Crépieux P, Reiter E, Spaggiari G, Brigante G, Casarini L, et al. Follicle-Stimulating Hormone (FSH) Action on Spermatogenesis: A Focus on Physiological and Therapeutic Roles. JCM. 2020 Apr 3;9(4):1014.

55. Nyongesa A, Patel N, Wango E, Onyango D. High khat dose and long-term exposure impairs spermatogenesis: experimental study using rabbit model. J Morphol Sci. 2017 July;34(03):156–67.

56. Nyongesa A, Oduma J, Oduor D. Khat (Catha Edulis) Addiction, Effects on General Body Health and Interventional Remedial Measures Drug Abuse: Addiction and Recovery. 2019.

Figure 1

Click here to access/download;Figure;Fig 1_Ouma et al 2025.tiff

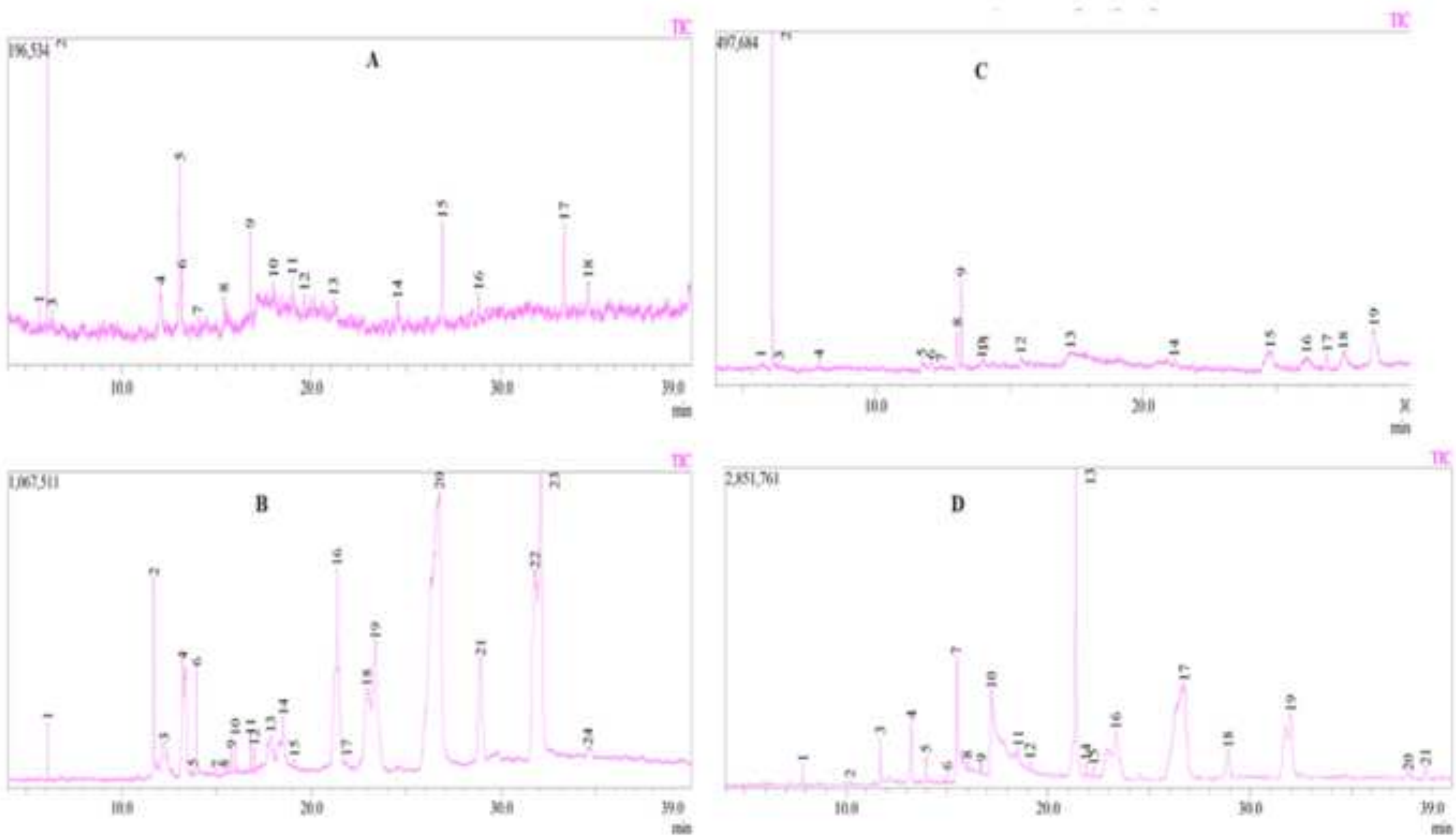

Figure 2

Click here to access/download;Figure;Fig 2_Ouma et al 2025.tiff

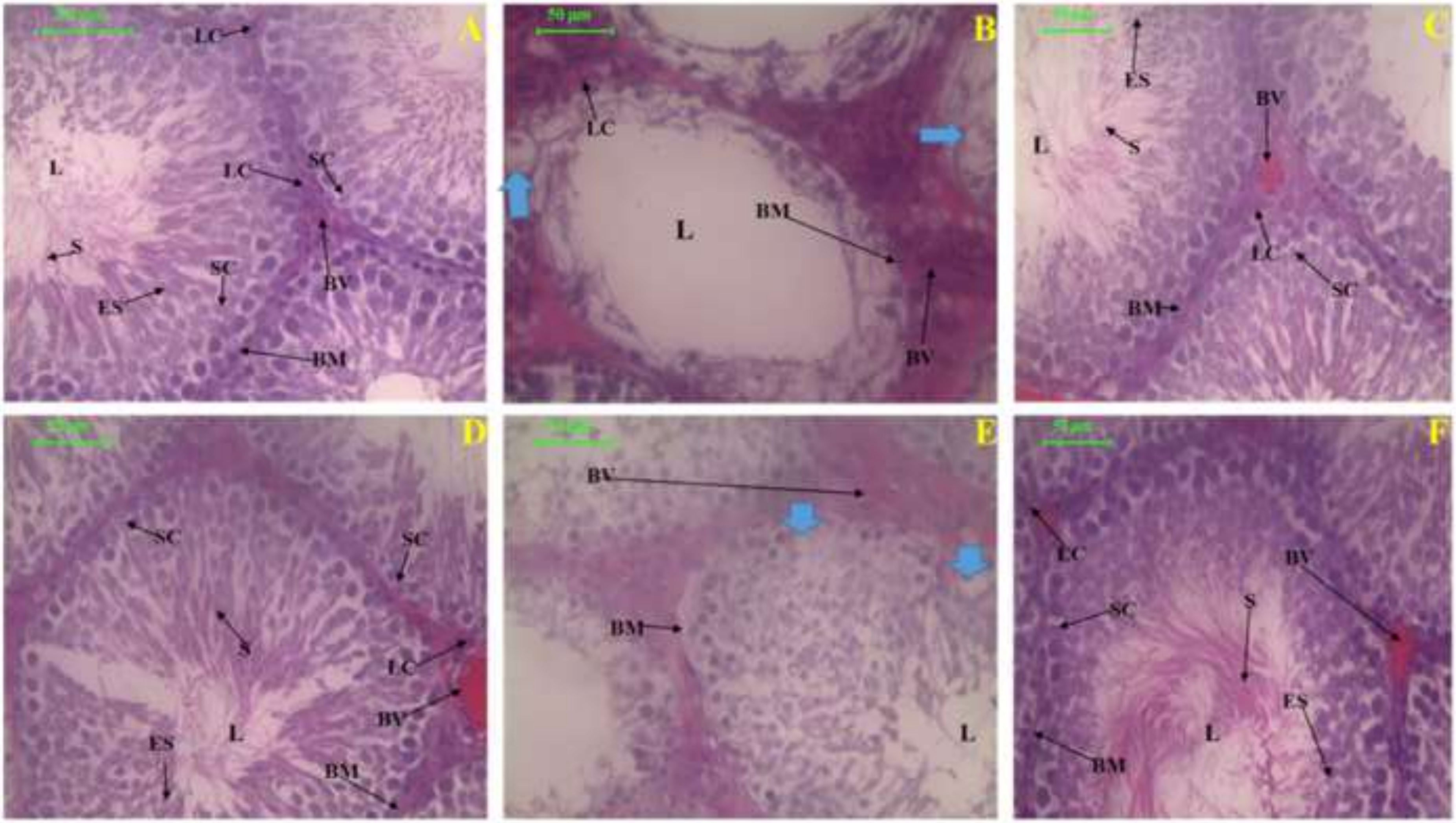

Click here to access/download
Supporting Information
Supplementary Material_Ouma et al.docx
