# Supplementary material for: Carissa spinarum L. aqueous root extracts ameliorate oxidative stress-induced infertility in adult male Wistar rats
Source: PLoS One. 2026 Aug 11;21(8):e0355380. doi: 10.1371/journal.pone.0355380 (PMC13460572; doi:10.1371/journal.pone.0355380)
Supplement: Supplementary file 3 [file pone.0355380.s001.docx]

**S1 Table**. **Reference sequences that matched BLASTn Search of the query sequences**. For *rbcl*, *Carissa haematocarpa (*JQ014161.1)*,* and *Carissa bispinosa (*AJ419738.1) had the highest percent identity of 99.7% each. For *trnH-psbA*, Carissa spinarum of accessions KR735499.1 and KR735438.1 recorded the highest percent identity of 99.23 each.

| ***rbcl*** | | | ***trnH-psbA*** | | | |
| --- | --- | --- | --- | --- | --- | --- |
| Reference species based on BLASTn | GenBank Accession Number | Percent identity (%) | Reference species based on BLASTn | GenBank Accession Number | Percent identity (%) |  |
| *Carissa haematocarpa* | JQ014161.1 | 99.70 | *Carissa spinarum* | KR735499.1 | 99.23 |  |
| *Carissa bispinosa* | AJ419738.1 | 99.70 | *Carissa spinarum* | KR735438.1 | 99.23 |  |
| *Carissa bispinosa* | JQ014160.1 | 99.56 | *Carissa spinarum* | KR735402.1 | 99.23 |  |
| *A.oblongifolia* | X91758.1 | 99.41 | *Carissa spinarum* | MK477715.1 | 96.15 |  |
| *Acokanthera oppositifolia* | AM234828.1 | 99.26 | *Carissa spinarum* | MK477714.1 | 95.15 |  |
| *Melodinus fusiformis* | EU916738.1 | 99.11 | *Rauvolfia manni* | KC667663.1 | 81.56 |  |
| *Melodinus suaveolens* | EU916737.1 | 99.11 | *Rauvolfia mannii* | KC667607.1 | 81.56 |  |
| *Amsonia tomentosa* | MF963214.1 | 98.82 | *Rauvolfia mannii* | KC667757.1 | 81.47 |  |
| *Gonioma kamassi* | AM234836.1 | 98.82 | *Rauvolfia vomitoria* | KC667822.1 | 80.47 |  |
| *Rhazya stricta* | AM295086.1 | 98.82 | *Rauvolfia vomitoria* | KC667792.1 | 80.47 |  |
| *Amsonia tabernaemontana* | AM295078.1 | 98.82 | *Rauvolfia verticillata* | KP095425.1 | 80.33 |  |
| *Pleiocarpa mutica* | AJ419757.1 | 98.82 | *Cerbera manghas* | OK636628.1 | 88.85 |  |
| *Craspidospermum verticillatum* | AJ419742.1 | 98.67 | *Cerbera manghas* | OK636629.1 | 88.85 |  |
| *Pleiocarpa rostrata* | KC628152.1 | 98.67 | *Cerbera manghas* | OK636630.1 | 88.85 |  |
