# Supplementary material for: Carissa spinarum L. aqueous root extracts ameliorate oxidative stress-induced infertility in adult male Wistar rats
Source: PLoS One. 2026 Aug 11;21(8):e0355380. doi: 10.1371/journal.pone.0355380 (PMC13460572; doi:10.1371/journal.pone.0355380)
Supplement: Supplementary file 4 [file pone.0355380.s002.docx]

**S2 Table. Volatile compounds detected in *C. spinarum* leaves and roots extracted with acetone and ethanol.**

| Peak name | Solvents/peak number/retention time | Peak Area (%) | Plant part | Molecular weight (g/mol) | | Reported Pharmacological activities |
| --- | --- | --- | --- | --- | --- | --- |
| 2,6-Dimethyl-6-nitro-2-hepten-4-one | Acetone/1/5.694 | 0.65 | Leaves | | 185.22 | Antifungal  [1] |
|  | Acetone/1/5.696 | 0.30 | Roots | |  |  |
| Ethanone,1-(2-hydroxyphenyl)- | Ethanol/1/6.125 | 0.45 | leaves | | 136.15 | No reported pharmacologic activity |
|  | Acetone/2/6.121 | 12.10 | leaves | |  |  |
|  | Acetone/2/6.120 | 21.97 | Roots | |  |  |
| Benzenemethanol,2-hydroxy-5-methyl- | Ethanol/1/7.873 | 0.38 | Roots | | 138.16 | No reported pharmacologic activity |
| Tetradecanal | Ethanol/2/11.696 | 2.31 | Leaves | | 212.37 | Derivatives exhibit antimicrobial, antifungal, and anti-cancerous properties [2]. |
|  | Ethanol/3/11.696 | 1.46 | Roots | |  |  |
| 9-Oxononanoic acid | Ethanol/2/10.200 | 0.14 | Roots | | 172.22 | Inhibitor of acetyl-CoA carboxylase (anti-obesity, anti –diabetes, and anti-tumor) [3], [4] |
| 3-Heptanol, 6-methyl- | Acetone/3/6.376 | 0.45 | Leaves | | 130.23 | Fungicidal activities [5] |
|  | Acetone/3/6.381 | 0.51 | Roots | |  |  |
| 3-Methylmannoside | Acetone/4/12.074 | 7.31 | leaves | | 194.18 | Antioxidant, anti-inflammatory, anti-diabetic, anti-bacterial, antifungal [6] |
| alpha.-Methyl mannofuranoside | Ethanol/3/12.249 | 1.49 | Leaves | | 194.18 | Antioxidant, antibacterial, and anticancer activities [7] |
|  | Acetone/6/12.248 | 1.18 | Roots | |  |  |
| 3-O-Methyl-d-glucose | Ethanol/4/13.235 | 3.66 | Roots | | 194.18 | No significant reports |
|  | Ethanol/4/13.220 | 6.78 | leaves | |  |  |
|  | Acetone/8/13.110 | 7.01 | Roots | |  |  |
| 4-O-Methylmannose | Acetone/5/13.092 | 26.64 | Leaves | | 194.18 | Antibacterial activities [8].anti-alopecic, anti-cirrhotic,anti-neuropathic, cholesterolytic [9] |
| Cyclopentaneundecanoic acid, methyl ester | Ethanol/7/15.013 | 0.05 | Leaves | | 268.4 | Antioxidant, and antibacterial [10]. |
| Pentadecanoic acid | Ethanol/7/15.843 | 6.22 | Roots | | 242.40 | Protects cardiometabolism, immunity and liver. It also has anticancer activities [11], [12] |
| n-Hexadecanoic acid | Acetone/8/15.407 | 1.86 | Leaves | | 256.42 | Analgesic, neuroprotective, and anti-neuroinflammatory. Prostaglandin-E29-reductase inhibitor  [13]. |
|  | Ethanol/8/15.413 | 0.05 | Leaves | |  |  |
|  | Acetone/12/15.416 | 1.44 | Roots | |  |  |
| 9-Octadecenal, (Z)- | Ethanol/9/15.782 | 0.05 | Leaves | | 266.5 | Antioxidans and Antibacterial [14] |
| 7-Hexadecenal, (Z)- | Ethanol/5/13.744 | 0.07 | Leaves | | 238.41 | No reported pharmacologic activity |
| 9-Octadecenoic acid (Z)-, methyl ester | Ethanol/9/16.706 | 0.30 | Roots | | 296.5 | Hepato-protective, antioxidant, anti-arthritis, antimicrobial, antibacterial, anti-cancerous and nematicide [15]. |
| Isophytol, acetate | Acetone/9/16.788 | 5.58 | Leaves | | 338.0 | No pharmacologic activity reported |
| 13-Oxabicyclo[10.1.0]tridecane | Acetone/13/17.287 | 12.03 | Roots | | 182.30 | Antihyperlipidemia, antioxidant [16]. |
| Vitamin E | Acetone/18/34.551 | 2.68 | Leaves | | 430.7 | Antioxidant, anti-inflammatory [17]. |
| Urs-12-en-3-ol, acetate, (3.beta.)- | Ethanol/22/31.755 | 5.43 | Leaves | | 468.8 | Antioxidant, anti-inflammatory [18]. |
| alpha.-Tocopherol-.beta.-D-mannoside | Ethanol/24/34.457 | 0.60 | Leaves | | 592.8 | Antidiabetic, anti-inflammatory [19] |
| Stigmasterol | Ethanol/21/38.670 | 0.92 | Roots | | 412.7 | Antioxidant, anticancer, antidiabetic, anti-osteoarthritic, anti-hyper-cholesterolemia [20]. |
| Tributyl acetylcitrate | Acetone/10/18.005 | 0.67 | Leaves | | 402.5 | No pharmacologic activity reported |
| Cyclohexanone, 2-(1-methyl-2-nitroethyl)- | Acetone/14/24.525 | 5.56 | Leaves | | 185.22 | No pharmacologic activity reported |
| Olean-12-en-3-ol, acetate, (3.beta.)- | Ethanol/21/28.899 | 6.77 | Leaves | | 468.8 | Antiviral, antibacterial [21]. |
|  | Ethanol/18/28.901 | 3.74 | Roots | |  |  |
| Z-(13,14-Epoxy)tetradec-11-en-1-ol acetate | Ethanol/15/22.246 | 0.34 | Roots | | 268.39 | Anti-inflammatory[22]. |
| 2-methyloctacosane | Acetone/16/28.791 | 2.62 | Leaves | | 408.8 | No pharmacologic activity reported. |
| Ergost-5-en-3.beta.-ol | Ethanol/20/37.810 | 0.38 | Roots | | 400.7 | Antioxidant, anticancer, [23]. |
| Tetratetracontane | Acetone/17/33.285 | 10.79 | Leaves | | 619.2 | Antibacterial, and antioxidant [24]. |
|  | Acetone/15/33.298 | 11.98 | Roots | |  |  |
| 2-Hexyldecyl acetate | Acetone/14/21.166 | 0.53 | Roots | | 284.5 | No pharmacologic activity reported. |
| beta.-Amyrone | Ethanol/14/18.515 | 0.68 | Leaves | | 424.7 | Anti-inflammatory, antifungal, antiobesity, antiviral [25]. |
| Squalene | Acetone/16/26.884 | 12.28 | Leaves | | 410.7 | Antiobesity, anticancerous, antioxidant [26]. |
|  | Acetone/17/26.890 | 2.83 | Roots | |  |  |
| 2H-Pyran-2-one, tetrahydro-6-tridecyl- | Ethanol/15/26.093 | 0.05 | Leaves | | 282.5 | Antimicrobial, antioxidant, anticancer [27]. |
| Androst-2-en-17-one, 4,4-dimethyl-, (5.alpha.)- | Acetone/12/19.624 | 2.41 | Leaves | | 300 | Androgenic steroid |
| Diisooctyl phthalate | Ethanol/16/21.346 | 11.71 | Leaves | | 390.6 | Antifungal |
|  | Ethanol/13/21.370 | 12.77 | Roots | |  |  |
| alpha.-Amyrin | Ethanol/18/22.909 | 2.38 | Leaves | | 426.7 | Anti-inflammatory, antinociceptive, antioxidant [28]. |
| beta.-Amyrin | Acetone/19/28.644 | 16.29 | Roots | | 426.7 | Anti-inflammatory, antinociceptive, antioxidant [28]. |
| Lup-20(29)-en-3-one | Ethanol/19/23.359 | 4.50 | Leaves | | 424.7 | Antioxidant, anticancer and anti-inflammatory [29]. |
|  | Ethanol/16/23.347 | 3.45 | Roots | |  |  |
| Lupeol | Ethanol/20/26.731 | 46.37 | Leaves | | 426.7 | Anti-inflammatory, antioxidant, and anticancer [29] |
|  | Acetone/18/26.730 | 3.57 | Roots | |  |  |
|  | Ethanol/17/26.739 | 3.45 | Roots | |  |  |
| Hexadecanoic acid, 2-hydroxy-1-(hydroxymethyl)ethyl ester | Acetone/13/21.162 | 2.21 | Leaves | | 330.5 | Anthelmintic, antioxidant, and anti-inflammatory activities [30] |
| Eicosanal | Ethanol/10/15.993 | 0.24 | Leaves | | 296.5 | Antioxidant, antidepressant, and anxiolytic effects [31]. |
| Estra-1,3,5(10)-trien-16-one, 3-[(trimethylsilyl)oxy]-  Or 16-Estrone, TMS derivative | Acetone/11/19.001 | 1.42 | Leaves | | 342 | Reproductive hormone replacement therapies |
| Phytol | Ethanol/11/16.786 | 0.26 | Leaves | | 296.5 | Antioxidant, anti-hyperalgesic, anti-inflammatory, anti-arthritic [32] |
| 1-Ethylsulfanylmethyl-2,8,9-trioxa-5-aza-1-sila-bicyclo[3.3.3]undecane | Ethanol/12/16.985 | 0.20 | Leaves | | 249.40 | No pharmacologic activity reported |
| 6-Undecyl-5,6-dihydro-2H-pyran-2-one | Ethanol/12/19.101 | 0.24 | Roots | | 252 | No pharmacologic activity reported |
|  | Ethanol/17/19.101 | 0.07 | Leaves | |  |  |
| gamma.-Sitosterol | Acetone/13/17.850 | 1.40 | Leaves | | 414.7 | Antidiabetic, anticancer [33]. |
| Hexadecanoic acid, methyl ester | Ethanol/6/15.009 | 0.22 | Roots | | 270.5 | Antimicrobial, anti-inflammatory antioxidant, , [34]. |
| Neophytadiene | Acetone/11/14.033 | 1.04 | Roots | | 278.5 | Antimicrobial, and anti-inflammatory properties [35]. |
|  | Acetone/7/14.036 | 0.45 | Leaves | |  |  |
| Methyl 3,4-ethylidene-.alpha.-D-galactopyranoside | Acetone/6/13.194 | 4.30 | Leaves | | 220.22 | No reported pharmacologic activity |
|  | Acetone/9/13.204 | 7.48 | Roots | |  |  |

**References**

[1]	A. A. K. Darmadi, S. K. Sudirga, N. L. Suriani, and I. G. A. S. Wahyuni, “Antifungal Activities Of Cinnamon Leaf Extracts Against Sigatoka Fungus (Pseudocercospora Fijiensis),” *IOP Conf. Ser.: Earth Environ. Sci.*, vol. 347, no. 1, p. 012051, Nov. 2019, doi: 10.1088/1755-1315/347/1/012051.

[2]	S. Javid *et al.*, “Semisynthesis of Myristic Acid Derivatives and their Biological Activities: A Critical Insight,” *Journal of Biologically Active Products from Nature*, vol. 10, no. 6, pp. 455–472, Nov. 2020, doi: 10.1080/22311866.2020.1865836.

[3]	L. Chen *et al.*, “Acetyl-CoA carboxylase (ACC) as a therapeutic target for metabolic syndrome and recent developments in ACC1/2 inhibitors,” *Expert Opinion on Investigational Drugs*, vol. 28, no. 10, pp. 917–930, Oct. 2019, doi: 10.1080/13543784.2019.1657825.

[4]	PubChem, “9-Oxononanoic acid.” Accessed: Jun. 01, 2024. [Online]. Available: https://pubchem.ncbi.nlm.nih.gov/compound/75704

[5]	M. A. A. Mangoba and D. D. G. Alvindia, “Fungicidal activities of Cymbopogon winterianus against anthracnose of banana caused by Colletotrichum musae,” *Sci Rep*, vol. 13, no. 1, p. 6629, Apr. 2023, doi: 10.1038/s41598-023-33396-5.

[6]	S. A. Mary *et al.*, “Investigation on Centrifugally Spun Fibrous PCL/3-Methyl Mannoside Mats for Wound Healing Application,” *Polymers*, vol. 15, no. 5, p. 1293, Mar. 2023, doi: 10.3390/polym15051293.

[7]	T. Baishya *et al.*, “Antioxidant activity, cytotoxicity assay, and evaluation of bioactive compounds using GC-MS and HPTLC-based bioassay in the extracts of Osbeckia stellata var. crinita (Benth. ex Naudin) grown in Manipur, India,” *Kuwait Journal of Science*, vol. 51, no. 3, p. 100229, Jul. 2024, doi: 10.1016/j.kjs.2024.100229.

[8]	O. D. Nurhayat *et al.*, “First identification of potential bioactive compounds from ethanol extracts of Lepista sordida from Indonesia,” *IOP Conf. Ser.: Earth Environ. Sci.*, vol. 1271, no. 1, p. 012058, Dec. 2023, doi: 10.1088/1755-1315/1271/1/012058.

[9]	N. U. Rehman, M. N. Ansari, W. Ahmad, and M. Amir, “GC–MS Analysis and In Vivo and Ex Vivo Antidiarrheal and Antispasmodic Effects of the Methanolic Extract of Acacia nilotica,” *Molecules*, vol. 27, no. 7, p. 2107, Mar. 2022, doi: 10.3390/molecules27072107.

[10]	D. A.O and T. Temikotan, “Fatty acid profile, antioxidant and antibacterial effect of the ethyl acatate extract of cleistopholis patens,” *Bull. Sci. Res.*, pp. 21–31, Feb. 2021, doi: 10.34256/bsr2113.

[11]	N. B. To, Y. T.-K. Nguyen, J. Y. Moon, M. K. Ediriweera, and S. K. Cho, “Pentadecanoic Acid, an Odd-Chain Fatty Acid, Suppresses the Stemness of MCF-7/SC Human Breast Cancer Stem-Like Cells through JAK2/STAT3 Signaling,” *Nutrients*, vol. 12, no. 6, p. 1663, Jun. 2020, doi: 10.3390/nu12061663.

[12]	S. Venn-Watson and N. J. Schork, “Pentadecanoic Acid (C15:0), an Essential Fatty Acid, Shares Clinically Relevant Cell-Based Activities with Leading Longevity-Enhancing Compounds,” *Nutrients*, vol. 15, no. 21, p. 4607, Oct. 2023, doi: 10.3390/nu15214607.

[13]	G. Carta, E. Murru, S. Banni, and C. Manca, “Palmitic Acid: Physiological Role, Metabolism and Nutritional Implications,” *Front. Physiol.*, vol. 8, p. 902, Nov. 2017, doi: 10.3389/fphys.2017.00902.

[14]	S. Hatami, A. Mohamadi Sani, and M. Yavarmanesh, “Chemical composition and antibacterial activity of organic extra virgin olive oil from Iran,” *NFS*, vol. 46, no. 3, pp. 388–395, May 2016, doi: 10.1108/NFS-01-2016-0010.

[15]	V. P.R. and J. P.A., “CHEMICAL PROFILING AND ANTIOXIDANT POTENTIAL OF DYSOXYLUM MALABARICUM SEEDS,” *PLANT ARCHIVES*, vol. 23, no. 2, Oct. 2023, doi: 10.51470/PLANTARCHIVES.2023.v23.no2.037.

[16]	S. N. Al-Otaibi, G. M. Alshammari, F. H. AlMohanna, A. S. Al-Khalifa, and M. A. Yahya, “Antihyperlipidemic and hepatic antioxidant effects of Leek leaf methanol extract in high fat diet-fed rats,” *All Life*, vol. 13, no. 1, pp. 373–385, Jan. 2020, doi: 10.1080/26895293.2020.1792355.

[17]	Y. Raji *et al.*, “Impact of α-tocopherol on Metronidazole and Tetracycline-induced Alterations in Reproductive Activities of Male Albino Rats,” *J. of Biological Sciences*, vol. 7, no. 1, pp. 41–46, Dec. 2006, doi: 10.3923/jbs.2007.41.46.

[18]	N. N. Okoye, D. L. Ajaghaku, H. N. Okeke, E. E. Ilodigwe, C. S. Nworu, and F. B. C. Okoye, “beta-Amyrin and alpha-amyrin acetate isolated from the stem bark of *Alstonia boonei* display profound anti-inflammatory activity,” *Pharmaceutical Biology*, vol. 52, no. 11, pp. 1478–1486, Nov. 2014, doi: 10.3109/13880209.2014.898078.

[19]	T. Ponrasu, R. E. Charles, R. Sivakumar, and S. Divakar, “Syntheses of α-tocopheryl glycosides by glucosidases,” *Biotechnol Lett*, vol. 30, no. 8, pp. 1431–1439, Aug. 2008, doi: 10.1007/s10529-008-9702-9.

[20]	H. Ullah and M. Daglia, “Phytonutrients in the management of glucose metabolism,” in *The Role of Phytonutrients in Metabolic Disorders*, Elsevier, 2022, pp. 163–193. doi: 10.1016/B978-0-12-824356-5.00011-4.

[21]	M. Dutta *et al.*, “Appraisals of the Bangladeshi Medicinal Plant Calotropis gigantea Used by Folk Medicine Practitioners in the Management of COVID-19: A Biochemical and Computational Approach,” *Front. Mol. Biosci.*, vol. 8, p. 625391, May 2021, doi: 10.3389/fmolb.2021.625391.

[22]	M. Musa *et al.*, “Pharmacological activities and gas chromatography–mass spectrometry analysis for the identification of bioactive compounds from Justicia adhatoda L.,” *Front. Pharmacol.*, vol. 13, p. 922388, Sep. 2022, doi: 10.3389/fphar.2022.922388.

[23]	O. A. Ojo *et al.*, “Phytochemical properties and pharmacological activities of the genus Pennisetum: A review,” *Scientific African*, vol. 16, p. e01132, Jul. 2022, doi: 10.1016/j.sciaf.2022.e01132.

[24]	T. Rhetso, R. Shubharani, M. S. Roopa, and V. Sivaram, “Chemical constituents, antioxidant, and antimicrobial activity of Allium chinense G. Don,” *Futur J Pharm Sci*, vol. 6, no. 1, p. 102, Dec. 2020, doi: 10.1186/s43094-020-00100-7.

[25]	P. D. O. De Almeida, A. P. D. A. Boleti, A. L. Rüdiger, G. A. Lourenço, V. F. Da Veiga Junior, and E. S. Lima, “Anti-Inflammatory Activity of Triterpenes Isolated from *Protium paniculatum* Oil-Resins,” *Evidence-Based Complementary and Alternative Medicine*, vol. 2015, pp. 1–10, 2015, doi: 10.1155/2015/293768.

[26]	M. A. Lozano-Grande, S. Gorinstein, E. Espitia-Rangel, G. Dávila-Ortiz, and A. L. Martínez-Ayala, “Plant Sources, Extraction Methods, and Uses of Squalene,” *International Journal of Agronomy*, vol. 2018, pp. 1–13, Aug. 2018, doi: 10.1155/2018/1829160.

[27]	M. Rajput, N. Bithel, and S. Vijayakumar, “Antimicrobial, antibiofilm, antioxidant, anticancer, and phytochemical composition of the seed extract of Pongamia pinnata,” *Arch Microbiol*, vol. 203, no. 7, pp. 4005–4024, Sep. 2021, doi: 10.1007/s00203-021-02365-9.

[28]	H. Singh, S. Bhushan, R. Arora, H. Singh Buttar, S. Arora, and B. Singh, “Alternative treatment strategies for neuropathic pain: Role of Indian medicinal plants and compounds of plant origin-A review,” *Biomedicine & Pharmacotherapy*, vol. 92, pp. 634–650, Aug. 2017, doi: 10.1016/j.biopha.2017.05.079.

[29]	K. Liu *et al.*, “Lupeol and its derivatives as anticancer and anti-inflammatory agents: Molecular mechanisms and therapeutic efficacy,” *Pharmacological Research*, vol. 164, p. 105373, Feb. 2021, doi: 10.1016/j.phrs.2020.105373.

[30]	H. A. M. Ali, H. H. Imad, and A. I. Salah, “Analysis of bioactive chemical components of two medicinal plants (Coriandrum sativum and Melia azedarach) leaves using gas chromatography-mass spectrometry (GC-MS),” *Afr. J. Biotechnol.*, vol. 14, no. 40, pp. 2812–2830, Oct. 2015, doi: 10.5897/AJB2015.14956.

[31]	Md. Z. Uddin *et al.*, “Chemical Profiles and Pharmacological Properties with in Silico Studies on Elatostema papillosum Wedd,” *Molecules*, vol. 26, no. 4, p. 809, Feb. 2021, doi: 10.3390/molecules26040809.

[32]	A. M. S. Carvalho *et al.*, “Phytol, a Chlorophyll Component, Produces Antihyperalgesic, Anti-inflammatory, and Antiarthritic Effects: Possible NFκB Pathway Involvement and Reduced Levels of the Proinflammatory Cytokines TNF-α and IL-6,” *J. Nat. Prod.*, vol. 83, no. 4, pp. 1107–1117, Apr. 2020, doi: 10.1021/acs.jnatprod.9b01116.

[33]	S. Sundarraj *et al.*, “γ-Sitosterol from Acacia nilotica L. induces G2/M cell cycle arrest and apoptosis through c-Myc suppression in MCF-7 and A549 cells,” *Journal of Ethnopharmacology*, vol. 141, no. 3, pp. 803–809, Jun. 2012, doi: 10.1016/j.jep.2012.03.014.

[34]	V. Gupta, S. Tyagi, and R. Tripathi, “Hexadecanoic acid methyl ester, a potent hepatoprotective compound in leaves of Pistia stratiotes L.,” *TABCJ*, pp. 118–120, Dec. 2023, doi: 10.52679/tabcj.2023.0012.

[35]	M. L. Gonzalez-Rivera, J. C. Barragan-Galvez, D. Gasca-Martínez, S. Hidalgo-Figueroa, M. Isiordia-Espinoza, and A. J. Alonso-Castro, “In Vivo Neuropharmacological Effects of Neophytadiene,” *Molecules*, vol. 28, no. 8, p. 3457, Apr. 2023, doi: 10.3390/molecules28083457.
