# Supplementary material for: Carissa spinarum L. aqueous root extracts ameliorate oxidative stress-induced infertility in adult male Wistar rats
Source: PLoS One. 2026 Aug 11;21(8):e0355380. doi: 10.1371/journal.pone.0355380 (PMC13460572; doi:10.1371/journal.pone.0355380)
Supplement: Supplementary file 5 [file pone.0355380.s003.docx]

**S3 Table.** Correlation coefficients of TPC, TFC, TTC, TRAC, and FRAP, HPRS

|  | Roots | | Leaves | |
| --- | --- | --- | --- | --- |
| Polyphenol (Concentration) | Correlation Coefficient (r) for FRAP | Correlation Coefficient (r) for HPRS | Correlation Coefficient (r)for FRAP | Correlation Coefficient(r) for HPRS |
| Phenolics | 0.713 | 0.735 | 0.780 | 0.563 |
| Flavonoids | -0.254^*^ | 0.852 | 0.723 | 0.614 |
| Tannins | 0.531 | 0.751 | 0.392^*^ | 0.673 |
| Alkaloids | 0.042^*^ | 0.613 | 0.214^*^ | 0.932 |

*The strength and direction of the relationships between the three polyphenolic groups, alkaloids across all solvents and FRAP, HPRS activity based on Pearson’s correlation analysis. Correlations that were not statistically significant (p > 0.05) are noted with an asterisk.
