# Supplementary material for: Carissa spinarum L. aqueous root extracts ameliorate oxidative stress-induced infertility in adult male Wistar rats
Source: PLoS One. 2026 Aug 11;21(8):e0355380. doi: 10.1371/journal.pone.0355380 (PMC13460572; doi:10.1371/journal.pone.0355380)
Supplement: Supplementary file 6 [file pone.0355380.s018.docx]

Manuscript Number: PONE-D-25-63093

*Carissa spinarum* L. aqueous root extracts ameliorate oxidative stress-induced infertility in adult male Wistar rats

**Response to Editor’s and Reviewers’ comments and suggestions**

**Academic Editor’s Comments**

1. Please ensure that your manuscript meets PLOS ONE's style requirements, including those for file naming. The PLOS ONE style templates can be found at <https://journals.plos.org/plosone/s/file?id=wjVg/PLOSOne_formatting_sample_main_body.pdf> and [https://journals.plos.org/plosone/s/file?id=ba62/PLOSOne_formatting_sample_title_authors_affiliations.pdf](https://track.editorialmanager.com/CL0/https:%2F%2Fjournals.plos.org%2Fplosone%2Fs%2Ffile%3Fid=ba62%2FPLOSOne_formatting_sample_title_authors_affiliations.pdf/1/010f019e2bb7ae06-1b69f93f-41ae-473f-a4eb-943c49f86b1d-000000/SOuERAXoGPksfVM0gRuvkjy3w2WEHyWC_EL2m1etE7M=258)

Response: We have revised the manuscript and aligned it with PLOS ONE style requirements including the file naming.

2. We note that your Data Availability Statement is currently as follows: All relevant data are within the manuscript and in Supporting Information files.

Please confirm at this time whether or not your submission contains all raw data required to replicate the results of your study. Authors must share the “minimal data set” for their submission. PLOS defines the minimal data set to consist of the data required to replicate all study findings reported in the article, as well as related metadata and methods (<https://journals.plos.org/plosone/s/data-availability#loc-minimal-data-set-definition>).

For example, authors should submit the following data:

- The values behind the means, standard deviations and other measures reported;

- The values used to build graphs;

- The points extracted from images for analysis.

Authors do not need to submit their entire data set if only a portion of the data was used in the reported study.

If your submission does not contain these data, please either upload them as Supporting Information files or deposit them to a stable, public repository and provide us with the relevant URLs, DOIs, or accession numbers. For a list of recommended repositories, please see <https://journals.plos.org/plosone/s/recommended-repositories>.

If there are ethical or legal restrictions on sharing a de-identified data set, please explain them in detail (e.g., data contain potentially sensitive information, data are owned by a third-party organization, etc.) and who has imposed them (e.g., an ethics committee). Please also provide contact information for a data access committee, ethics committee, or other institutional body to which data requests may be sent. If data are owned by a third party, please indicate how others may request data access.

**Response:** We have provided all the raw data sets (images and values used to generate the mean) used to produce all the results in this study in the supplementary materials as a zip folder.

3. Your ethics statement should only appear in the Methods section of your manuscript. If your ethics statement is written in any section besides the Methods, please delete it from any other section.

**Response:** We have revised the manuscript with the ethical statement only appearing in the methodology section of the manuscript.

4. Please include captions for your Supporting Information files at the end of your manuscript, and update any in-text citations to match accordingly. Please see our Supporting Information guidelines for more information: [http://journals.plos.org/plosone/s/supporting-information](http://track.editorialmanager.com/CL0/http:%2F%2Fjournals.plos.org%2Fplosone%2Fs%2Fsupporting-information/1/010f019e2bb7ae06-1b69f93f-41ae-473f-a4eb-943c49f86b1d-000000/C-5lrJzHzGNlq957K1Lv4P5qCW-Ityx5IeUmnzVf3xs=258).

**Response:** We have revised and included all captions of the supporting materials at the end of the manuscript.

5. PLOS ONE now requires that authors provide the original uncropped and unadjusted images underlying all blot or gel results reported in a submission’s figures or Supporting Information files. This policy and the journal’s other requirements for blot/gel reporting and figure preparation are described in detail at <https://journals.plos.org/plosone/s/figures#loc-blot-and-gel-reporting-requirements> and <https://journals.plos.org/plosone/s/figures#loc-preparing-figures-from-image-files>. When you submit your revised manuscript, please ensure that your figures adhere fully to these guidelines and provide the original underlying images for all blot or gel data reported in your submission. See the following link for instructions on providing the original image data: <https://journals.plos.org/plosone/s/figures#loc-original-images-for-blots-and-gels>.

In your cover letter, please note whether your blot/gel image data are in Supporting Information or posted at a public data repository, provide the repository URL if relevant, and provide specific details as to which raw blot/gel images, if any, are not available. Email us at [plosone@plos.org](mailto:plosone@plos.org) if you have any questions.

**Response:** We have provided all the raw gel and histological images used in this study in the supplementary material in zip folder.

6. If the reviewer comments include a recommendation to cite specific previously published works, please review and evaluate these publications to determine whether they are relevant and should be cited. There is no requirement to cite these works unless the editor has indicated otherwise.

**Response:** With regards to citations, the reviewers did not recommend any additional references for citation; however, we have ensured that the citations and references meet the PLOS ONE requirements.

7. Please review your reference list to ensure that it is complete and correct. If you have cited papers that have been retracted, please include the rationale for doing so in the manuscript text, or remove these references and replace them with relevant current references. Any changes to the reference list should be mentioned in the rebuttal letter that accompanies your revised manuscript. If you need to cite a retracted article, indicate the article’s retracted status in the References list and also include a citation and full reference for the retraction notice.

**Response:** We also revised the reference list and added reference numbers [6, 7, 8, 27, 28, 36, 41] to provide updated and more conclusive information supporting this study in various aspects. The following references in the originally submitted manuscripts were replaced: [6, 33, 38]. These additions have been incorporated into the relevant sections of the manuscript and are highlighted in the revised version. We have not cited any retracted paper in the current manuscript.

**Reviewer #1:**

The article entitled "*Carissa spinarum* L. aqueous root extracts ameliorate oxidative stress-induced infertility in adult male wistar rats" is interesting and shall be revised before considering in this journal for publication.

1. In the abstract, state which solvent extract is used for the in vivo study.

**Response:** Thank you for the observation comment. We have revised the abstract and provided the solvent extract (aqueous root extracts) used for the *in vivo* study. Please see page 2, lines 35, 36 and 46 of the abstract of the revised manuscript.

2. In the methodology, clearly state in *in vivo* study, about which solvent's extract have been studied,

**Response:** Thank you for this observation. We have revised this by including the solvent used in the in vivo study (aqueous root extract) in the methodology section. Please see page 10, lines 290 – 294 of the revised manuscript.

3. The number of animals used is 5, justify the sample calculation method or how the number is selected.

**Response:** The sample size (n=5) was selected based on previously published study evaluating comparable biological outcomes and, on the need, to statistically detect observable treatment-related effects, while minimizing unnecessary laboratory animal use, as guided by ethical requirements. The relevant article has been cited as citation number 27 in the study design section (please see page 10, line 284 of the revised manuscript).

4. State the study followed ARRIVE guidelines along with EU guidelines

**Response:** Thank you for this comment. The manuscript has now been revised to indicate that the study followed the Animal Research: Reporting of *in vivo* Experiments (ARRIVE), along with European Community guidelines (EEC Directive of 1986; 86/609/EEC). Please page 10 of the manuscript, lines 278 – 279 of the revised manuscript.

5. Sketch some of the major phytochemical compounds profiled by GCMS

**Response:** Thank you for the valuable suggestion. We have now added sketches of major GC-MS-identified compounds (Fig 2) that have been reported to exhibit antioxidant activity and to improve male reproductive parameters. Please see page 20 of the manuscript.

6. Discuss the rationale of selecting this plant and extract for the specific study.

**Response:** Thank you for this valuable observation. We have indicated in the Introduction section, page 1 of the manuscript (Lines 74–91), the rationale for selecting this plant and extract for the study. Specifically, we highlighted that the plant has extensive ethnobotanical reports supporting its traditional use in managing male infertility and other ailments. However, despite its widespread traditional use, there is limited scientific evidence explaining the mechanisms underlying these reported therapeutic effects. Based on these traditional claims and the plant’s rich phytochemical profile, we hypothesized that the aqueous root extract, prepared in a manner consistent with traditional use, could improve male fertility parameters, including sperm count, motility, and vitality.

7. Conclusion shall be specific and mention the predicted hormonal influence and the steroidal activation related to the plant.

**Response:** Thank you for this important observation. The Conclusion section has now been revised to present the predicted hormonal influence and the possible activation of the steroidogenic pathway associated with *C. spinarum* aqueous root extracts. These interpretations are based on observed improvements in sperm characteristics and the restoration of normal testicular histology post-treatment. Please see lines 858 – 861 of the revised manuscript.

**Reviewer #2:**

*Carissa spinarum* L. aqueous root extracts ameliorate oxidative stress-induced infertility in adult male Wistar rats’’ is a well-organized study. All the objectives of research are clear and well-designed. Minor corrections need to be considered.

- Some typing and grammatical errors were observed. Please correct it.

**Response**: Dear reviewer, thank you for this important observation. The manuscript has now been revised and corrected for grammatical and spelling errors as suggested. Please see the revised manuscript with track changes as well as the manuscript.

- Drugs and chemicals should be written separately.

**Response:** Thank you for this valuable input. The manuscript has now been revised to include separate sections on chemicals and drugs under the methodology section, page 5 of the revised manuscript.

- The statistical symbols (for comparison) within tables and figures are not defined in legends. Kindly include its details for clarity.

**Response:** Thank you for the valuable observation. The manuscript has now been revised, and all statistical symbols used in comparisons are defined in each figure/table caption. The changes were made to table 1 (page 16), table 2 (page 18), table 3 (page 21), and table 4 (page 23) of the revised manuscripts. Additionally, we made the changes in figure captions of the supplementary material, including figure S3, S4, S5, S6, S8, 10 and S11.

- How the doses were selected, is there any previous study?

**Response:** Thank you for this important observation. The doses used in this study were selected based on the findings from the acute oral toxicity study, which established the safe range of the extracts. By way of explanation, based on this, a starting lower dose of 250 mg/kg bw was escalated by a factor of 2 to give 500 mg/kg bw and 1000 mg/kg bw to investigate the efficacy of different doses. We also considered previously published reports of safe, pharmacologically relevant doses of *C. spinarum* in rat models (Citation 28). This clarification is presented in lines 284-287 of the revised manuscript.

**Reviewer #3:**

This is a well-designed and highly valuable pharmacological study. Validating the traditional medicinal use of *Carissa spinarum* for managing male infertility is a great contribution to reproductive biology and natural product research.

I appreciate your comprehensive methodology. Using metronidazole to induce oxidative stress in the Wistar rat model provided a robust baseline, and your evaluation of both the phytochemical profiles and *in vivo* parameters (sperm characteristics, biochemical assays, and histology) gives a very complete picture. The clear efficacy demonstrated at the 500 mg/kg dose for improving sperm count and reducing lipid peroxidation is particularly compelling.

I have no changes to request. Congratulations on a rigorous and well-executed paper.

**Response:** We sincerely thank you for the positive evaluation, comments, and recognition of the scientific significance of our study investigating the therapeutic potential of *Carissa spinarum* in male reproductive health.

Yours sincerely

Martin Ouma (corresponding Author)	Signature Date 26/05/2026
