# Supplementary material for: Carissa spinarum L. aqueous root extracts ameliorate oxidative stress-induced infertility in adult male Wistar rats
Source: PLoS One. 2026 Aug 11;21(8):e0355380. doi: 10.1371/journal.pone.0355380 (PMC13460572; doi:10.1371/journal.pone.0355380)
Supplement: Peer review history

# Peer review: Decision Letter 0

**DOI:** 10.1371/journal.pone.0355380.r001

**Authors:** Saira Saeed Khan

15 May 2026

PONE-D-25-63093

Carissa spinarum L. aqueous root extracts ameliorate oxidative stress-induced infertility in adult male wistar rats

PLOS One

Dear Dr. Ouma,

Thank you for submitting your manuscript to PLOS ONE. After careful consideration, we feel that it has merit but does not fully meet PLOS ONE’s publication criteria as it currently stands. Therefore, we invite you to submit a revised version of the manuscript that addresses the points raised during the review process.

Please submit your revised manuscript by Jun 29 2026 11:59PM. If you will need more time than this to complete your revisions, please reply to this message or contact the journal office at plosone@plos.org. When you're ready to submit your revision, log on to https://www.editorialmanager.com/pone/ and select the 'Submissions Needing Revision' folder to locate your manuscript file.

Please include the following items when submitting your revised manuscript:

- A letter that responds to each point raised by the academic editor and reviewer(s). You should upload this letter as a separate file labeled 'Response to Reviewers'.
- A marked-up copy of your manuscript that highlights changes made to the original version. You should upload this as a separate file labeled 'Revised Manuscript with Track Changes'.
- An unmarked version of your revised paper without tracked changes. You should upload this as a separate file labeled 'Manuscript'.

If you would like to make changes to your financial disclosure, please include your updated statement in your cover letter. Guidelines for resubmitting your figure files are available below the reviewer comments at the end of this letter.

If applicable, we recommend that you deposit your laboratory protocols in protocols.io to enhance the reproducibility of your results. Protocols.io assigns your protocol its own identifier (DOI) so that it can be cited independently in the future. For instructions see: https://journals.plos.org/plosone/s/submission-guidelines#loc-laboratory-protocols. Additionally, PLOS ONE offers an option for publishing peer-reviewed Lab Protocol articles, which describe protocols hosted on protocols.io. Read more information on sharing protocols at https://plos.org/protocols?utm_medium=editorial-email&utm_source=authorletters&utm_campaign=protocols.

As the corresponding author, your ORCID iD is verified in the submission system and will appear in the published article. PLOS supports the use of ORCID, and we encourage all coauthors to register for an ORCID iD and use it as well. Please encourage your coauthors to verify their ORCID iD within the submission system before final acceptance, as unverified ORCID iDs will not appear in the published article. *Only*  the individual author can complete the verification step; PLOS staff *cannot*  verify ORCID iDs on behalf of authors.

We look forward to receiving your revised manuscript.

Kind regards,

Saira Saeed Khan, Ph.D.

Academic Editor

PLOS One

Journal Requirements:

When submitting your revision, we need you to address these additional requirements.

1.Please ensure that your manuscript meets PLOS ONE's style requirements, including those for file naming. The PLOS ONE style templates can be found at

https://journals.plos.org/plosone/s/file?id=wjVg/PLOSOne_formatting_sample_main_body.pdf and

https://journals.plos.org/plosone/s/file?id=ba62/PLOSOne_formatting_sample_title_authors_affiliations.pdf

2. We note that your Data Availability Statement is currently as follows: All relevant data are within the manuscript and in Supporting Information files.

Please confirm at this time whether or not your submission contains all raw data required to replicate the results of your study. Authors must share the “minimal data set” for their submission. PLOS defines the minimal data set to consist of the data required to replicate all study findings reported in the article, as well as related metadata and methods (https://journals.plos.org/plosone/s/data-availability#loc-minimal-data-set-definition).

For example, authors should submit the following data:

- The values behind the means, standard deviations and other measures reported;

- The values used to build graphs;

- The points extracted from images for analysis.

Authors do not need to submit their entire data set if only a portion of the data was used in the reported study.

If your submission does not contain these data, please either upload them as Supporting Information files or deposit them to a stable, public repository and provide us with the relevant URLs, DOIs, or accession numbers. For a list of recommended repositories, please see https://journals.plos.org/plosone/s/recommended-repositories.

If there are ethical or legal restrictions on sharing a de-identified data set, please explain them in detail (e.g., data contain potentially sensitive information, data are owned by a third-party organization, etc.) and who has imposed them (e.g., an ethics committee). Please also provide contact information for a data access committee, ethics committee, or other institutional body to which data requests may be sent. If data are owned by a third party, please indicate how others may request data access.

3. Your ethics statement should only appear in the Methods section of your manuscript. If your ethics statement is written in any section besides the Methods, please delete it from any other section.

4. Please include captions for your Supporting Information files at the end of your manuscript, and update any in-text citations to match accordingly. Please see our Supporting Information guidelines for more information: http://journals.plos.org/plosone/s/supporting-information.

5. PLOS ONE now requires that authors provide the original uncropped and unadjusted images underlying all blot or gel results reported in a submission’s figures or Supporting Information files. This policy and the journal’s other requirements for blot/gel reporting and figure preparation are described in detail at https://journals.plos.org/plosone/s/figures#loc-blot-and-gel-reporting-requirements and https://journals.plos.org/plosone/s/figures#loc-preparing-figures-from-image-files. When you submit your revised manuscript, please ensure that your figures adhere fully to these guidelines and provide the original underlying images for all blot or gel data reported in your submission. See the following link for instructions on providing the original image data: https://journals.plos.org/plosone/s/figures#loc-original-images-for-blots-and-gels.

In your cover letter, please note whether your blot/gel image data are in Supporting Information or posted at a public data repository, provide the repository URL if relevant, and provide specific details as to which raw blot/gel images, if any, are not available. Email us at plosone@plos.org if you have any questions.

6. If the reviewer comments include a recommendation to cite specific previously published works, please review and evaluate these publications to determine whether they are relevant and should be cited. There is no requirement to cite these works unless the editor has indicated otherwise.

7. Please review your reference list to ensure that it is complete and correct. If you have cited papers that have been retracted, please include the rationale for doing so in the manuscript text, or remove these references and replace them with relevant current references. Any changes to the reference list should be mentioned in the rebuttal letter that accompanies your revised manuscript. If you need to cite a retracted article, indicate the article’s retracted status in the References list and also include a citation and full reference for the retraction notice.

[Note: HTML markup is below. Please do not edit.]

Reviewers' comments:

Reviewer's Responses to Questions

**Comments to the Author**

1. Is the manuscript technically sound, and do the data support the conclusions?

The manuscript must describe a technically sound piece of scientific research with data that supports the conclusions. Experiments must have been conducted rigorously, with appropriate controls, replication, and sample sizes. The conclusions must be drawn appropriately based on the data presented.

Reviewer #1: Yes

Reviewer #2: Yes

Reviewer #3: Yes

**********

2. Has the statistical analysis been performed appropriately and rigorously?

Reviewer #1: Yes

Reviewer #2: Yes

Reviewer #3: Yes

**********

3. Have the authors made all data underlying the findings in their manuscript fully available?

The PLOS Data policy requires authors to make all data underlying the findings described in their manuscript fully available without restriction, with rare exception (please refer to the Data Availability Statement in the manuscript PDF file). The data should be provided as part of the manuscript or its supporting information, or deposited to a public repository. For example, in addition to summary statistics, the data points behind means, medians and variance measures should be available. If there are restrictions on publicly sharing data—e.g. participant privacy or use of data from a third party—those must be specified.

Reviewer #1: Yes

Reviewer #2: Yes

Reviewer #3: Yes

**********

4. Is the manuscript presented in an intelligible fashion and written in standard English?

PLOS ONE does not copyedit accepted manuscripts, so the language in submitted articles must be clear, correct, and unambiguous. Any typographical or grammatical errors should be corrected at revision, so please note any specific errors here.

Reviewer #1: Yes

Reviewer #2: Yes

Reviewer #3: Yes

**********

5. Review Comments to the Author

Please use the space provided to explain your answers to the questions above. You may also include additional comments for the author, including concerns about dual publication, research ethics, or publication ethics. (Please upload your review as an attachment if it exceeds 20,000 characters)

Reviewer #1: The article entitled " Carissa spinarum L. aqueous root extracts ameliorate oxidative stress-induced

infertility in adult male wistar rats" is interesting and shall be revised before considering in this journlal for publication.

1. In the abstract, state which solvent extract is used for the invivo study.

2. In the methodology, clearly state in invivo study, about which solvent's extract have been studied,

3. The number of animals used is 5, justtify the sample calculation method or how the number is selected.

4. State the study followed ARRIVE guidelines along with EU guidelines

5. Skecth some of the major phytochemical compounds profiled by GCMS

6. Discuss the rationale of selecting this plant and extract for the specific study.

7. Conclusion shall be specific and mention the predicted hormonal influence and the steroidal activation related to the plant.

Reviewer #2: Carissa spinarum L. aqueous root extracts ameliorate oxidative stress-induced infertility in adult male Wistar rats’’ is a well-organized study. All the objectives of research are clear and well-designed. Minor corrections need to be considered.

•Some typing and grammatical errors were observed. Please correct it.

•Drugs and chemicals should be written separately.

•The statistical symbols (for comparison) within tables and figures are not defined in legends. Kindly include its details for clarity.

•How the doses were selected, is there any previous study?

Reviewer #3: This is a well-designed and highly valuable pharmacological study. Validating the traditional medicinal use of Carissa spinarum for managing male infertility is a great contribution to reproductive biology and natural product research.

I appreciate your comprehensive methodology. Using metronidazole to induce oxidative stress in the Wistar rat model provided a robust baseline, and your evaluation of both the phytochemical profiles and in vivo parameters (sperm characteristics, biochemical assays, and histology) gives a very complete picture. The clear efficacy demonstrated at the 500 mg/kg dose for improving sperm count and reducing lipid peroxidation is particularly compelling.

I have no changes to request. Congratulations on a rigorous and well-executed paper.

**********

6. PLOS authors have the option to publish the peer review history of their article (what does this mean?). If published, this will include your full peer review and any attached files.

If you choose “no”, your identity will remain anonymous but your review may still be made public.

**Do you want your identity to be public for this peer review?**  For information about this choice, including consent withdrawal, please see our Privacy Policy.

Reviewer #1: No

Reviewer #2: No

Reviewer #3: **Yes:**  Ganesh Praneeth Roy Avula

**********

[NOTE: If reviewer comments were submitted as an attachment file, they will be attached to this email and accessible via the submission site. Please log into your account, locate the manuscript record, and check for the action link "View Attachments". If this link does not appear, there are no attachment files.]

To ensure your figures meet our technical requirements, please review our figure guidelines: https://journals.plos.org/plosone/s/figures

You may also use PLOS’s free figure tool, NAAS, to help you prepare publication quality figures: https://journals.plos.org/plosone/s/figures#loc-tools-for-figure-preparation.

NAAS will assess whether your figures meet our technical requirements by comparing each figure against our figure specifications.

Attachment Submitted filename: PONE-D-25-63093_reviewer.pdf

# Author comment: Author response to Decision Letter 1

**DOI:** 10.1371/journal.pone.0355380.r002

16 Jun 2026

Response to Editor’s and Reviewers’ comments and suggestions

Academic Editor’s Comments

1. Please ensure that your manuscript meets PLOS ONE's style requirements, including those for file naming. The PLOS ONE style templates can be found at https://journals.plos.org/plosone/s/file?id=wjVg/PLOSOne_formatting_sample_main_body.pdf and https://journals.plos.org/plosone/s/file?id=ba62/PLOSOne_formatting_sample_title_authors_affiliations.pdf

Response: We have revised the manuscript and aligned it with PLOS ONE style requirements including the file naming.

2. We note that your Data Availability Statement is currently as follows: All relevant data are within the manuscript and in Supporting Information files.

Please confirm at this time whether or not your submission contains all raw data required to replicate the results of your study. Authors must share the “minimal data set” for their submission. PLOS defines the minimal data set to consist of the data required to replicate all study findings reported in the article, as well as related metadata and methods (https://journals.plos.org/plosone/s/data-availability#loc-minimal-data-set-definition).

For example, authors should submit the following data:

- The values behind the means, standard deviations and other measures reported;

- The values used to build graphs;

- The points extracted from images for analysis.

Authors do not need to submit their entire data set if only a portion of the data was used in the reported study.

If your submission does not contain these data, please either upload them as Supporting Information files or deposit them to a stable, public repository and provide us with the relevant URLs, DOIs, or accession numbers. For a list of recommended repositories, please see https://journals.plos.org/plosone/s/recommended-repositories.

If there are ethical or legal restrictions on sharing a de-identified data set, please explain them in detail (e.g., data contain potentially sensitive information, data are owned by a third-party organization, etc.) and who has imposed them (e.g., an ethics committee). Please also provide contact information for a data access committee, ethics committee, or other institutional body to which data requests may be sent. If data are owned by a third party, please indicate how others may request data access.

Response: We have provided all the raw data sets (images and values used to generate the mean) used to produce all the results in this study in the supplementary materials as a zip folder.

3. Your ethics statement should only appear in the Methods section of your manuscript. If your ethics statement is written in any section besides the Methods, please delete it from any other section.

Response: We have revised the manuscript with the ethical statement only appearing in the methodology section of the manuscript.

4. Please include captions for your Supporting Information files at the end of your manuscript, and update any in-text citations to match accordingly. Please see our Supporting Information guidelines for more information: http://journals.plos.org/plosone/s/supporting-information.

Response: We have revised and included all captions of the supporting materials at the end of the manuscript.

5. PLOS ONE now requires that authors provide the original uncropped and unadjusted images underlying all blot or gel results reported in a submission’s figures or Supporting Information files. This policy and the journal’s other requirements for blot/gel reporting and figure preparation are described in detail at https://journals.plos.org/plosone/s/figures#loc-blot-and-gel-reporting-requirements and https://journals.plos.org/plosone/s/figures#loc-preparing-figures-from-image-files. When you submit your revised manuscript, please ensure that your figures adhere fully to these guidelines and provide the original underlying images for all blot or gel data reported in your submission. See the following link for instructions on providing the original image data: https://journals.plos.org/plosone/s/figures#loc-original-images-for-blots-and-gels.

In your cover letter, please note whether your blot/gel image data are in Supporting Information or posted at a public data repository, provide the repository URL if relevant, and provide specific details as to which raw blot/gel images, if any, are not available. Email us at plosone@plos.org if you have any questions.

Response: We have provided all the raw gel and histological images used in this study in the supplementary material in zip folder.

6. If the reviewer comments include a recommendation to cite specific previously published works, please review and evaluate these publications to determine whether they are relevant and should be cited. There is no requirement to cite these works unless the editor has indicated otherwise.

Response: With regards to citations, the reviewers did not recommend any additional references for citation; however, we have ensured that the citations and references meet the PLOS ONE requirements.

7. Please review your reference list to ensure that it is complete and correct. If you have cited papers that have been retracted, please include the rationale for doing so in the manuscript text, or remove these references and replace them with relevant current references. Any changes to the reference list should be mentioned in the rebuttal letter that accompanies your revised manuscript. If you need to cite a retracted article, indicate the article’s retracted status in the References list and also include a citation and full reference for the retraction notice.

Response: We also revised the reference list and added reference numbers [6, 7, 8, 27, 28, 36, 41] to provide updated and more conclusive information supporting this study in various aspects. The following references in the originally submitted manuscripts were replaced: [6, 33, 38]. These additions have been incorporated into the relevant sections of the manuscript and are highlighted in the revised version. We have not cited any retracted paper in the current manuscript.

Reviewer #1:

The article entitled "Carissa spinarum L. aqueous root extracts ameliorate oxidative stress-induced infertility in adult male wistar rats" is interesting and shall be revised before considering in this journal for publication.

1. In the abstract, state which solvent extract is used for the in vivo study.

Response: Thank you for the observation comment. We have revised the abstract and provided the solvent extract (aqueous root extracts) used for the in vivo study. Please see page 2, lines 35, 36 and 46 of the abstract of the revised manuscript.

2. In the methodology, clearly state in in vivo study, about which solvent's extract have been studied,

Response: Thank you for this observation. We have revised this by including the solvent used in the in vivo study (aqueous root extract) in the methodology section. Please see page 10, lines 290 – 294 of the revised manuscript.

3. The number of animals used is 5, justify the sample calculation method or how the number is selected.

Response: The sample size (n=5) was selected based on previously published study evaluating comparable biological outcomes and, on the need, to statistically detect observable treatment-related effects, while minimizing unnecessary laboratory animal use, as guided by ethical requirements. The relevant article has been cited as citation number 27 in the study design section (please see page 10, line 284 of the revised manuscript).

4. State the study followed ARRIVE guidelines along with EU guidelines

Response: Thank you for this comment. The manuscript has now been revised to indicate that the study followed the Animal Research: Reporting of in vivo Experiments (ARRIVE), along with European Community guidelines (EEC Directive of 1986; 86/609/EEC). Please page 10 of the manuscript, lines 278 – 279 of the revised manuscript.

5. Sketch some of the major phytochemical compounds profiled by GCMS

Response: Thank you for the valuable suggestion. We have now added sketches of major GC-MS-identified compounds (Fig 2) that have been reported to exhibit antioxidant activity and to improve male reproductive parameters. Please see page 20 of the manuscript.

6. Discuss the rationale of selecting this plant and extract for the specific study.

Response: Thank you for this valuable observation. We have indicated in the Introduction section, page 1 of the manuscript (Lines 74–91), the rationale for selecting this plant and extract for the study. Specifically, we highlighted that the plant has extensive ethnobotanical reports supporting its traditional use in managing male infertility and other ailments. However, despite its widespread traditional use, there is limited scientific evidence explaining the mechanisms underlying these reported therapeutic effects. Based on these traditional claims and the plant’s rich phytochemical profile, we hypothesized that the aqueous root extract, prepared in a manner consistent with traditional use, could improve male fertility parameters, including sperm count, motility, and vitality.

7. Conclusion shall be specific and mention the predicted hormonal influence and the steroidal activation related to the plant.

Response: Thank you for this important observation. The Conclusion section has now been revised to present the predicted hormonal influence and the possible activation of the steroidogenic pathway associated with C. spinarum aqueous root extracts. These interpretations are based on observed improvements in sperm characteristics and the restoration of normal testicular histology post-treatment. Please see lines 858 – 861 of the revised manuscript.

Reviewer #2:

Carissa spinarum L. aqueous root extracts ameliorate oxidative stress-induced infertility in adult male Wistar rats’’ is a well-organized study. All the objectives of research are clear and well-designed. Minor corrections need to be considered.

• Some typing and grammatical errors were observed. Please correct it.

Response: Dear reviewer, thank you for this important observation. The manuscript has now been revised and corrected for grammatical and spelling errors as suggested. Please see the revised manuscript with track changes as well as the manuscript.

• Drugs and chemicals should be written separately.

Response: Thank you for this valuable input. The manuscript has now been revised to include separate sections on chemicals and drugs under the methodology section, page 5 of the revised manuscript.

• The statistical symbols (for comparison) within tables and figures are not defined in legends. Kindly include its details for clarity.

Response: Thank you for the valuable observation. The manuscript has now been revised, and all statistical symbols used in comparisons are defined in each figure/table caption. The changes were made to table 1 (page 16), table 2 (page 18), table 3 (page 21), and table 4 (page 23) of the revised manuscripts. Additionally, we made the changes in figure captions of the supplementary material, including figure S3, S4, S5, S6, S8, 10 and S11.

• How the doses were selected, is there any previous study?

Response: Thank you for this important observation. The doses used in this study were selected based on the findings from the acute oral toxicity study, which established the safe range of the extracts. By way of explanation, based on this, a starting lower dose of 250 mg/kg bw was escalated by a factor of 2 to give 500 mg/kg bw and 1000 mg/kg bw to investigate the efficacy of different doses. We also considered previously published reports of safe, pharmacologically relevant doses of C. spinarum in rat models (Citation 28). This clarification is presented in lines 284-287 of the revised manuscript.

Reviewer #3:

This is a well-designed and highly valuable pharmacological study. Validating the traditional medicinal use of Carissa spinarum for managing male infertility is a great contribution to reproductive biology and natural product research.

I appreciate your comprehensive methodology. Using metronidazole to induce oxidative stress in the Wistar rat model provided a robust baseline, and your evaluation of both the phytochemical profiles and in vivo parameters (sperm characteristics, biochemical assays, and histology) gives a very complete picture. The clear efficacy demonstrated at the 500 mg/kg dose for improving sperm count and reducing lipid peroxidation is particularly compelling.

I have no changes to request. Congratulations on a rigorous and well-executed paper.

Response: We sincerely thank you for the positive evaluation, comments, and recognition of the scientific significance of our study investigating the therapeutic potential of Carissa spinarum in male reproductive health.

Yours sincerely

Martin Ouma (corresponding Author)

Attachment Submitted filename: Response to Editor and Reviewers Comments.docx

# Peer review: Decision Letter 1

**DOI:** 10.1371/journal.pone.0355380.r003

**Authors:** Saira Saeed Khan

22 Jul 2026

Carissa spinarum L. aqueous root extracts ameliorate oxidative stress-induced infertility in adult male wistar rats

PONE-D-25-63093R1

Dear Dr. Martin,

We’re pleased to inform you that your manuscript has been judged scientifically suitable for publication and will be formally accepted for publication once it meets all outstanding technical requirements.

Within one week, you’ll receive an e-mail detailing the required amendments. When these have been addressed, you’ll receive a formal acceptance letter and your manuscript will be scheduled for publication.

An invoice will be generated when your article is formally accepted. Please note, if your institution has a publishing partnership with PLOS and your article meets the relevant criteria, all or part of your publication costs will be covered. Please make sure your user information is up-to-date by logging into Editorial Manager at Editorial Manager® and clicking the ‘Update My Information' link at the top of the page. For questions related to billing, please contact billing support.

If your institution or institutions have a press office, please notify them about your upcoming paper to help maximize its impact. If they’ll be preparing press materials, please inform our press team as soon as possible -- no later than 48 hours after receiving the formal acceptance. Your manuscript will remain under strict press embargo until 2 pm Eastern Time on the date of publication. For more information, please contact onepress@plos.org.

Kind regards,

Saira Saeed Khan, Ph.D.

Academic Editor

PLOS One

Additional Editor Comments (optional):

Reviewers' comments:

Reviewer's Responses to Questions

**Comments to the Author**

1. If the authors have adequately addressed your comments raised in a previous round of review and you feel that this manuscript is now acceptable for publication, you may indicate that here to bypass the “Comments to the Author” section, enter your conflict of interest statement in the “Confidential to Editor” section, and submit your "Accept" recommendation.

Reviewer #1: All comments have been addressed

Reviewer #2: All comments have been addressed

**********

2. Is the manuscript technically sound, and do the data support the conclusions?

The manuscript must describe a technically sound piece of scientific research with data that supports the conclusions. Experiments must have been conducted rigorously, with appropriate controls, replication, and sample sizes. The conclusions must be drawn appropriately based on the data presented.

Reviewer #1: Yes

Reviewer #2: Yes

**********

3. Has the statistical analysis been performed appropriately and rigorously?

Reviewer #1: Yes

Reviewer #2: Yes

**********

4. Have the authors made all data underlying the findings in their manuscript fully available?

The PLOS Data policy requires authors to make all data underlying the findings described in their manuscript fully available without restriction, with rare exception (please refer to the Data Availability Statement in the manuscript PDF file). The data should be provided as part of the manuscript or its supporting information, or deposited to a public repository. For example, in addition to summary statistics, the data points behind means, medians and variance measures should be available. If there are restrictions on publicly sharing data—e.g. participant privacy or use of data from a third party—those must be specified.

Reviewer #1: Yes

Reviewer #2: Yes

**********

5. Is the manuscript presented in an intelligible fashion and written in standard English?

PLOS ONE does not copyedit accepted manuscripts, so the language in submitted articles must be clear, correct, and unambiguous. Any typographical or grammatical errors should be corrected at revision, so please note any specific errors here.

Reviewer #1: Yes

Reviewer #2: Yes

**********

6. Review Comments to the Author

Please use the space provided to explain your answers to the questions above. You may also include additional comments for the author, including concerns about dual publication, research ethics, or publication ethics. (Please upload your review as an attachment if it exceeds 20,000 characters)

Reviewer #1: The authors addressed the quries raised and amended in the manuscript. The manuscript shall be accepted as per the editorial board decision.

Reviewer #2: (No Response)

**********

7. PLOS authors have the option to publish the peer review history of their article (what does this mean?). If published, this will include your full peer review and any attached files.

If you choose “no”, your identity will remain anonymous but your review may still be made public.

**Do you want your identity to be public for this peer review?**  For information about this choice, including consent withdrawal, please see our Privacy Policy.

Reviewer #1: No

Reviewer #2: **Yes:**  Sadia K.

**********

# Editor report: Acceptance letter

**DOI:** 10.1371/journal.pone.0355380.r004

**Authors:** Saira Saeed Khan

PONE-D-25-63093R1

PLOS One

Dear Dr. Ouma,

I'm pleased to inform you that your manuscript has been deemed suitable for publication in PLOS One. Congratulations! Your manuscript is now being handed over to our production team.

At this stage, our production department will prepare your paper for publication. This includes ensuring the following:

* All references, tables, and figures are properly cited

* All relevant supporting information is included in the manuscript submission,

* There are no issues that prevent the paper from being properly typeset

You will receive further instructions from the production team, including instructions on how to review your proof when it is ready. Please keep in mind that we are working through a large volume of accepted articles, so please give us a few days to review your paper and let you know the next and final steps.

Lastly, if your institution or institutions have a press office, please let them know about your upcoming paper now to help maximize its impact. If they'll be preparing press materials, please inform our press team within the next 48 hours. Your manuscript will remain under strict press embargo until 2 pm Eastern Time on the date of publication. For more information, please contact onepress@plos.org.

You will receive an invoice from PLOS for your publication fee after your manuscript has reached the completed accept phase. If you receive an email requesting payment before acceptance or for any other service, this may be a phishing scheme. Learn how to identify phishing emails and protect your accounts at https://explore.plos.org/phishing.

If we can help with anything else, please email us at customercare@plos.org.

Thank you for submitting your work to PLOS ONE and supporting open access.

Kind regards,

PLOS ONE Editorial Office Staff

on behalf of

Dr. Saira Saeed Khan

Academic Editor

PLOS One
